# Iodine-mediated synthesis of 3-acylbenzothiadiazine 1,1-dioxides

**DOI:** 10.3762/bjoc.12.101

**Published:** 2016-05-24

**Authors:** Long-Yi Xi, Ruo-Yi Zhang, Lei Shi, Shan-Yong Chen, Xiao-Qi Yu

**Affiliations:** 1 Key Laboratory of Green Chemistry & Technology, Ministry of Education, College of Chemistry, Sichuan University, Chengdu 610064, PR China

**Keywords:** 3-acylbenzothiadiazine 1,1-dioxides, cyclization, metal-free, oxidation

## Abstract

An iodine-mediated synthesis of 3-acylbenzothiadizine 1,1-dioxides is described. A range of electronically diverse acetophenones reacted well with several 2-aminobenzenesulfonamides, affording 3-acylbenzothiadiazine 1,1-dioxides in good yields.

## Introduction

**Scheme 1:**
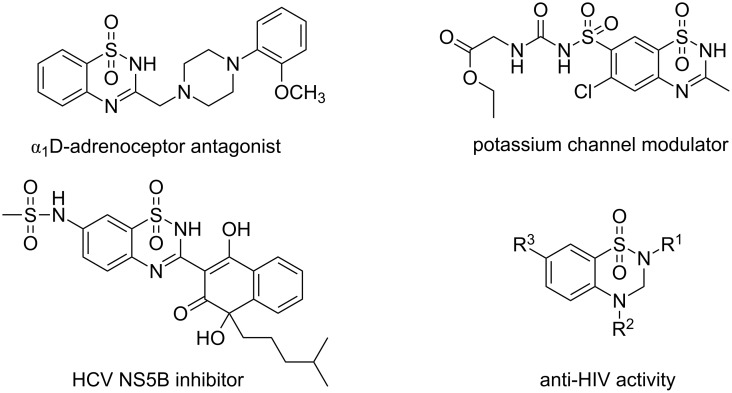
Selected benzothiadaiazine 1,1-dioxides with potent biological activities.

Benzothiadiazine 1,1-dioxide moieties have attracted remarkable attention in the pharmacological area because of their broad spectrum of activities [1–4], such as antihypertensive [5–6] or antiviral [7–8] and they are also used as cardiovascular agents [9–11] (Scheme 1). In this context, several synthetic methods have been developed to synthesize benzothiadiazine 1,1-dioxides and their analogues. The condensations of 2-aminobenzenesulfonamides with urea, isocyanates, carboxylic acid derivatives or other carbonyl reagents are the most used methods [12–15]. These reactions were usually carried out under harsh reaction conditions, causing the formation of byproducts. An alternative route to the benzothiadiazine 1,1-dioxide ring are transition metal-catalyzed reactions. Various benzothiadiazine 1,1-dioxides were successfully prepared using this approach [16–19]. However, the separation of transitional metal catalysts from pharmaceutical chemicals was cumbersome. Therefore, the development of efficient metal-free routes to benzothiadiazine 1,1-dioxides is necessary. On the other hand, despite that so many benzothiadiazine 1,1-dioxide derivatives have been studied extensively, 3-acylbenzothiadiazine 1,1-dioxides were ignored, because of the lack of efficient methods to prepare them. Readily available acetophenones have been shown to be good starting materials for the synthesis of various heterocyclic compounds [20–24]. The Wu group has reported an efficient protocol for the synthesis of luotonin F and derivatives from aromatic ketones and 2-aminobenzamides via iodination/Kornblum oxidation/annulation [25]. We envisioned that 2-aminobenzenesulfonamides would undergo a similar reaction to afford 3-acylbenzothiadiazine 1,1-dioxides. Herein, we report the first synthesis of 3-acylbenzothiadiazine 1,1-dioxides from 2-aminobenzenesulfonamides and acetophenones via iodine-mediated sp^3^ C–H functionalization.

## Results and Discussion

We commenced our studies by heating acetophenone (**1a**), 2-aminobenzenesulfonamide (**2a**) and I_2_ in DMSO for 12 h. To our delight, the desired product was isolated in 60% yield. To improve the yield of this product, a series of additives including acids, metal salt and bases were tested. However, to our disappointment, all of them could not promote this transformation (Table 1, entries 2–7). After optimizing the ratios of **1a** and **2a**, we found that increasing the amount of **1a** or **2a** resulted in lower yields (Table 1, entries 8 and 9). Iodinated compound **4a** was isolated as a major byproduct, therefore we set out to optimize the amount of I_2_ (Table 1, entry 1 and entries 10–14). 0.75 equiv of iodine gave the best result, and the yield increased to 73% (Table 1, entry 11). This result was different from the synthesis of quinazolin-4-ones reported by Wu group, in which reducing the amount of iodine leaded to low yield [25]. Raising or reducing temperature both resulted in decreased yields (Table 1, entries 15 and 16). Under argon atmosphere, the yield decreased slightly (Table 1, entry 17 vs entry 11), while it decreased obviously when the amount of I_2_ was reduced to 0.4 equiv (Table 1, entry 18 vs entry 14) [26]. We further found that the yield increased slightly to 80% by doubling the reaction time (Table 1, entry 19). Consequently, we decided to set the conditions described in entry 19 as the standard conditions.

**Table 1:** Optimization of reaction conditions.^a^

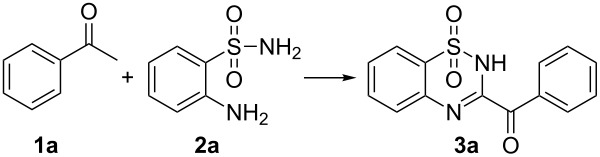				

entry	I**_2_** (equiv)	additives (equiv)	time (h)	yield (%)^b^

1	1.1	none	12	60
2	1.1	HI (0.1)	12	58
3	1.1	AcOH (0.1)	12	46
4	1.1	TsOH·H_2_O (0.1)	12	39
5	1.1	CuI (0.1)	12	51
6	1.1	KOH (0.1)	12	46
7	1.1	NaO*t*-Bu (0.1)	12	53
8**^c^**	1.1	none	12	55
9**^d^** 10	1.1 0.9	none none	12 12	41 59
11 12	0.75 0.6	none none	12 12	73 62
13	0.5	none	12	56
14 15^e^ 16^f^ 17^g^	0.4 0.75 0.75 0.75	none none none none	12 12 12 12	42 21 65 66
18^g^	0.4	none	12	25
**19**	**0.75**	**none**	**24**	**80**

^a^Reaction conditions: **1a** (0.33 mmol), **2a** (0.3 mmol), DMSO (2 mL). ^b^Isolated yields. ^c^1.5 equiv of acetophenone. ^d^1.5 equiv of 2-aminobenzenesulfonamide. ^e^80 °C. ^f^130 °C. ^g^Ar atmosphere.

**Scheme 2:**
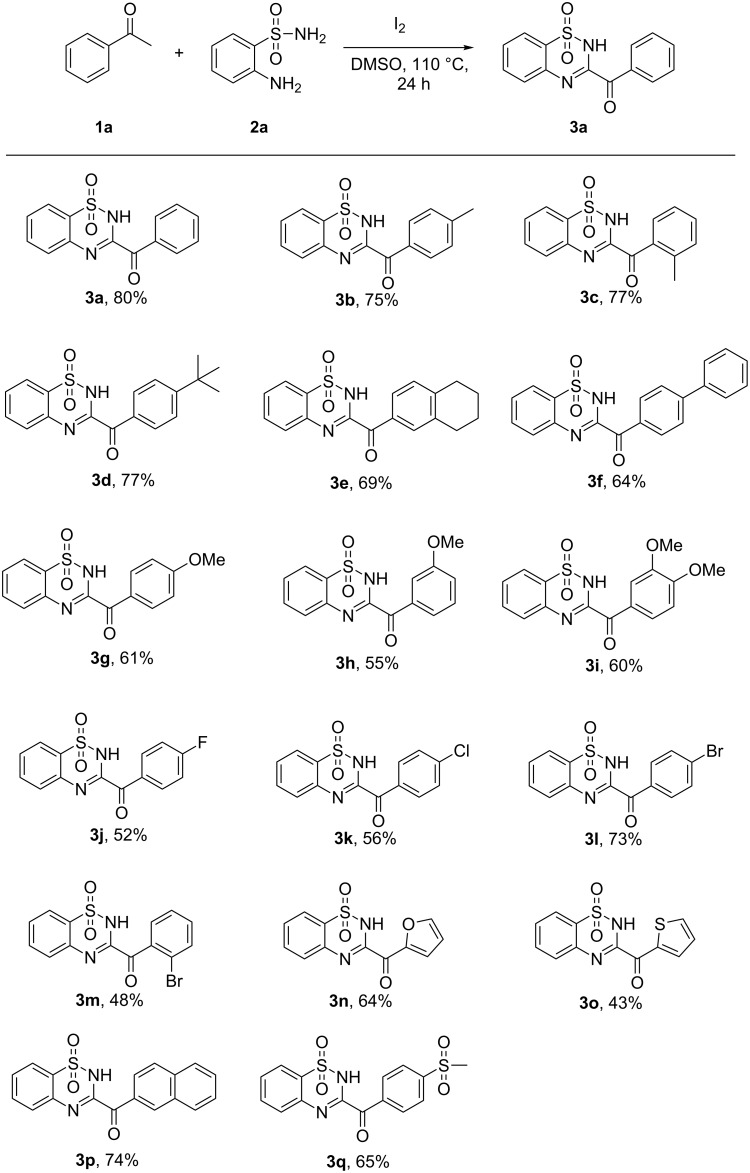
Scope of acetophenones (reaction conditions: **1** (0.33 mmol), **2a** (0.3 mmol), DMSO (2 mL), I_2_ (0.75 equiv), 110 °C, 24 h, isolated yields).

With optimized conditions in hand, we next explored the substrate scope of acetophenones. As shown in Scheme 2, a variety of acetophenones were compatible with this transformation, both electron-rich and electron-poor functional groups at the *para*-position of the benzene ring of acetophenones were well tolerated in this reaction. Acetophenones bearing halides (**1j–l**) such as fluoro, chloro and bromo substituents proceeded smoothly to give the corresponding products in good yields, which provided opportunities for further syntheses of more complex benzothiadiazine 1,1-dioxides. Besides acetophenones, heteroaryl methyl ketones also underwent this transformation, affording the corresponding products in moderate yields (**3n** and **3o**). When 1-(naphthalen-2-yl)ethanone was subjected to this reaction, a good yield of the desired product **3p** was isolated.

**Scheme 3:**
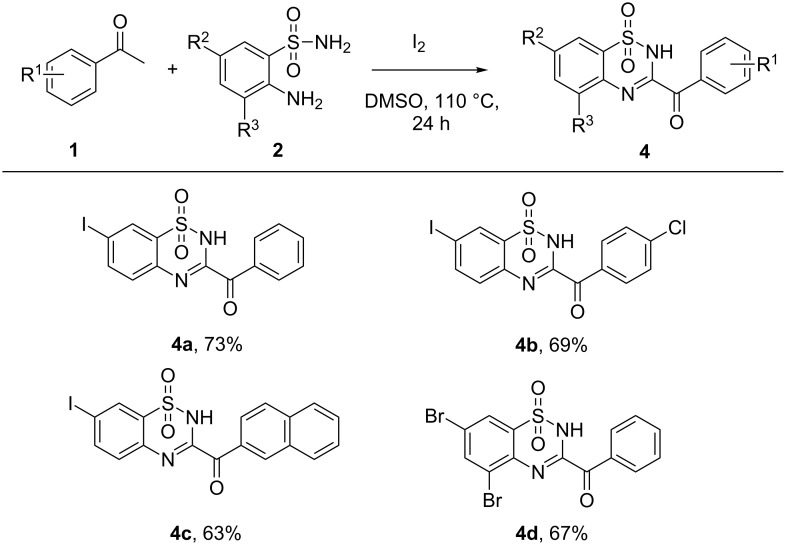
Scope of 2-aminobenzenesulfonamides (reaction conditions: **1** (0.33 mmol), **2a** (0.3 mmol), DMSO (2 mL), I_2_ (0.75 equiv), 110 °C, 24 h, isolated yields).

**Scheme 4:**
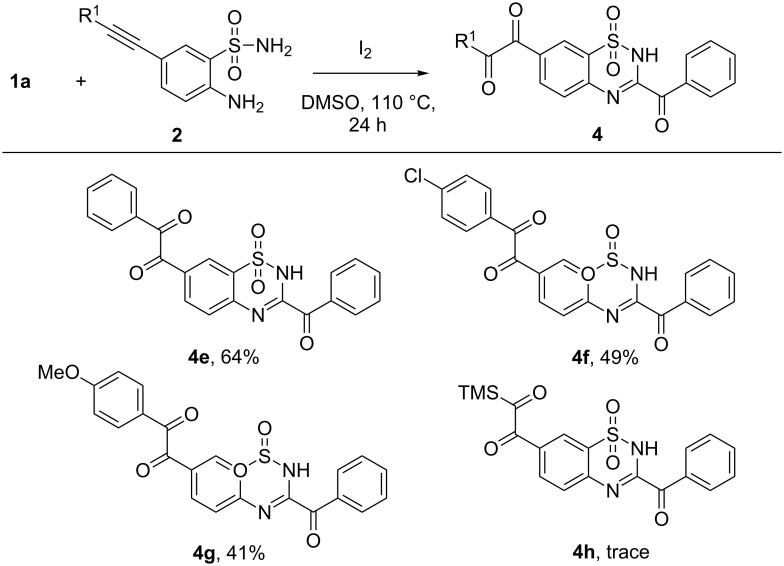
Reactions of 2-aminobenzenesulfonamides bearing an alknyl group (reaction conditions: **1** (0.33 mmol), **2** (0.3 mmol), DMSO (2 mL), I_2_ (0.75 equiv), 110 °C, 24 h, isolated yields.)

To widen the scope of substrates, we further explored several 2-aminobenzenesulfonamides bearing substituents on the benzene ring. 2-Aminobenzenesulfonamides bearing halides proceeded well, providing the corresponding products in good yields (Scheme 3). Interestingly, when 2-aminobenzenesulfonamides bearing an alknyl group were subjected to this reaction, besides the formation of 3-acylbenzothiadiazine 1,1,-dioxide skeletons, the triple bond was further transformed into an *ortho*-diketone functionality (Scheme 4) [27–29]. 1,2-Dicarbonyl functionalities are one of the most important skeletons found in biologically active molecules and versatile building blocks for chemical transformations [30–32].

**Scheme 5:**
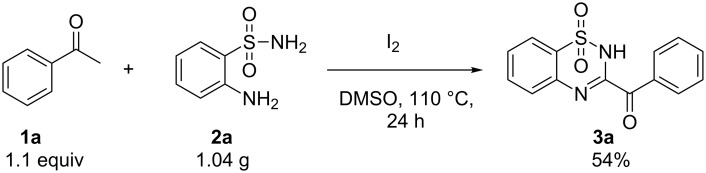
Gram scale reaction between **1a** and **2a**.

**Figure 1:**
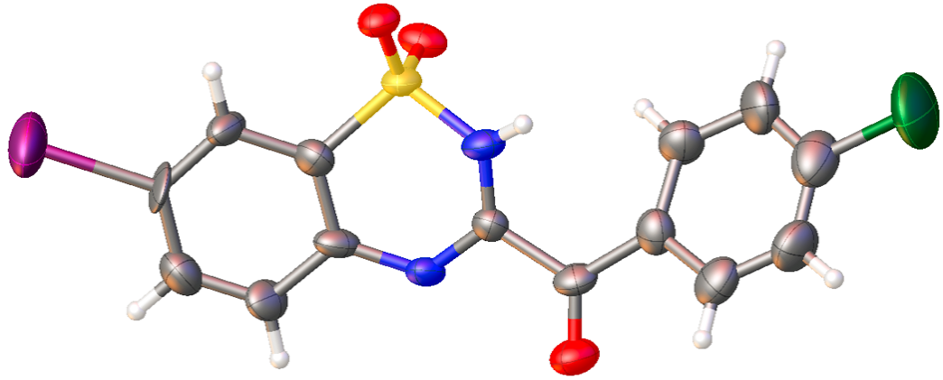
X-ray crystal structure of **4b** (CCDC 1444753).

In support of the application of this method, we conducted the reaction on a gram scale, and it also showed good performance (Scheme 5). The structure of **4b** was characterized by X-ray diffraction (Figure 1).

**Scheme 6:**
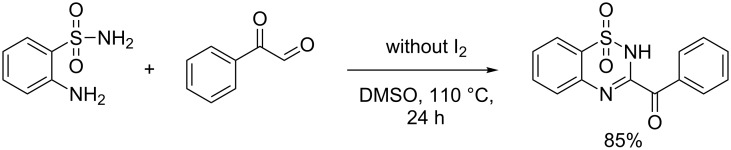
Control experiment.

To shed light on the mechanism of this reaction, a control experiment was conducted. It is known that acetophenones can undergo halogenation and further Kornblum oxidation to give phenylglyoxal in a I_2_/DMSO system [33–34]. We isolated the product in 85% yield by heating phenylglyoxal and **2a** in DMSO at 110 °C for 24 h without I_2_ (Scheme 6). To further probe the reaction process, we monitored the reaction system at 80 °C by ^1^H NMR spectroscopic studies. A signal appeared at 9.63 ppm, which was assigned to phenylglyoxal [35] (see Supporting Information File 1). Moreover, intermediate **D** was isolated after stirring 12 h at 80 °C.

**Scheme 7:**
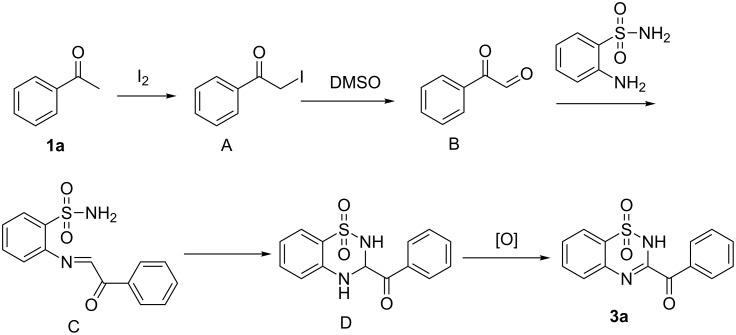
Proposed mechanism.

On the basis of this experiment described above and literature [13,35–36], a possible mechanism was proposed in Scheme 7. The halogenation of **1a** with iodine results in the formation of compound **A**, which, via Kornblum oxidation, provides phenylglyoxal **B**. The condensation of phenylglyoxal with 2-aminobenzenesulfonamide affords intermediate **C**, followed by intramolecular addition giving intermediate **D**. Intermediate **D** undergoes autoxidation leading to the desired product **3a**.

## Conclusion

In conclusion, we have developed a novel one-pot protocol for the synthesis of 3-acylbenzothiadiazine 1,1-dioxides from readily available aromatic ketones and 2-aminobenzenesulfonamides. Various aryl and heteroaryl ketones reacted well with 2-aminobenzenesulfonamides, affording the corresponding products in moderate to good yields. This metal-free method would provide opportunities to develop new biologically active benzothiadiazine 1,1-dioxides.

## Experimental

**Typical procedure for the synthesis of 3-acylbenzothiadiazine 1,1-dioxides:** A mixture of acetophenone (0.040 mL, 0.33 mmol), I_2_ (0.057 g, 0.225 mmol) and 2-aminobenzenesulfonamide (0.051 g, 0.3 mmol) in DMSO (2 mL) was stirred at 110 °C under air atmosphere in a sealed 50 mL Schlenk tube for 24 h. After the reaction was finished, the reaction mixture was cooled to room temperature. The resulting mixture was taken up by dichloromethane (60 mL) and washed with saturated Na_2_S_2_O_3_ solution until the brown color disappeared. The organic phase was dried over Na_2_SO_4_ (anhydrous), concentrated in vacuum, and the resulting residue was purified by column chromatography on silica gel with EtOAc/petroleum (1:4) to afford the product.

## Supporting Information

File 1 Experimental part and copies of NMR spectra.
