# Supplementary material for: Iodine-mediated synthesis of 3-acylbenzothiadiazine 1,1-dioxides
Source: Beilstein J Org Chem. 2016 May 24;12:1072–8. doi: 10.3762/bjoc.12.101 (PMC4902049; doi:10.3762/bjoc.12.101)

Supporting Information

for

Iodine-mediated synthesis of 3-acylbenzothiadiazine 1,1-dioxides

Long-Yi Xi, Ruo-Yi Zhang, Lei Shi, Shan-Yong Chen,* and Xiao-Qi Yu*[,§]

Address: Key Laboratory of Green Chemistry & Technology, Ministry of Education, College of Chemistry, Sichuan University, Chengdu 610064, PR China.

Email: Shan-Yong Chen* - chensy@scu.edu.cn; Xiao-Qi Yu* - xqyu@scu.edu.cn

*Corresponding author

[§]Tel.: (86)-28-85415886; fax: (86)-28-85415886

Experimental part and copies of NMR spectra

Table of contents

General……...…………………………………….……………………..……………….s2

Experimental procedures for substrates ……………..…………………………………..…s2–s3

Spectral data of substrates…….………....………………………………..………………...s2–s5

Typical procedure for the synthesis of 3-acyl benzothiadiazine 1,1-dioxides……………..……..s5

Molecular structure and crystallographic data of **4b**…………………………………….…s5–s6

Spectral data of intermediate D……………………………………………………………..…..s6

Monitoring the reaction using ^{1}H NMR and control experiments…………………………….s7–s8

Spectral data of compounds……………………………………………………………….s9–s16

Copies of ^{1}H and ^{13}C NMR spectra………...…………………………………………..…..s17–s47

General

Chemicals were purchased and used as received unless otherwise noted. All reactions were carried out in sealed Schlenk tubes and monitored by TLC. ^{1}H and ^{13}C NMR spectra were recorded on a Bruker Avance-III 400 instrument (400 MHz for ^{1}H and 100 MHz for ^{13}C NMR spectroscopy) d_6-DMSO was used as the solvent. Chemical shift values for ^{1}H and ^{13}C NMR were referred to internal Me_4Si (0 ppm). Mass spectra were recorded on a Thermo Scientific LCQ Fleet spectrometer with ESI mode.

Experimental procedures for substrates

Synthesis of 2-amino-5-iodobenzenesulfonamide

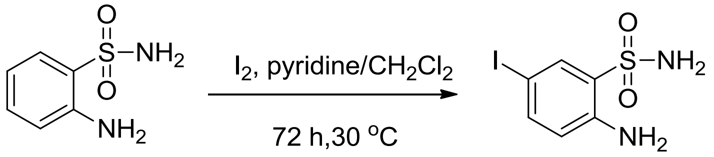

2-Aminobenzenesulfonamide (1.72 g, 10 mmol) was dissolved in CH_2Cl_2 (25 mL) and pyridine (25 mL). Iodine (10.32 g, 40 mmol) was slowly added into the mixture. The solution was stirred for 72 hour at 30 ^{o}C. A saturated $Na_2S_2O_3$ solution was then added until the brown color disappeared. The mixture was extracted with CH_2Cl_2 (50 mL) and washed with saturated brine solution (3 × 30 mL), followed by the evaporation of the solvent under reduced pressure. The residue was purified by column chromatography on silica gel affording 2-amino-5-iodobenzenesulfonamide as a white solid in 57% yield.

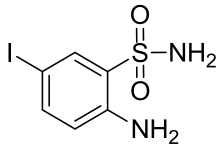

(**2b**)

White solid (1.70g, 57%)

^{1}H NMR (400 MHz, d_6-DMSO) δ 7.76 (s, 1H), 7.48 (d, J = 8.6 Hz, 1H), 7.35 (s, 2H), 6.65 (d, J = 8.6 Hz, 1H), 6.00 (s, 2H)

^{13}C NMR (100 MHz, d_6-DMSO) δ 145.07, 140.70, 135.43, 126.24, 119.32, 74.50

HRMS m/z (ESI) : calcd. for $[C_6H_7IN_2O_2S+Na]^+$: 320.9165 Found: 320.9151.

Synthesis of 2-amino-3,5-dibromobenzenesulfonamide

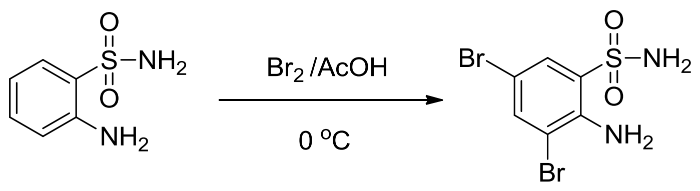

2-Aminobenzenesulfonamide (516 mg, 3 mmol) was dissolved in 7.5 mL AcOH, the solution was cooled to 0 °C. Br_2 (0.31 mL, 12 mmol) dissolved in 1.5 mL AcOH was added dropwise to a solution of 2-aminobenzenesulfonamide. The reaction mixture was slowly warmed to room temperature and stirred for a further 2 h. The resulting mixture was concentrated and taken up by dichloromethane (2 × 30 mL). The combined organic phases were dried over Na_2SO_4 (anhydrous) concentrated in vacuum and the residue recrystallized from EtOH/H_2O (1:3) to give the product 595.6 mg (44%).

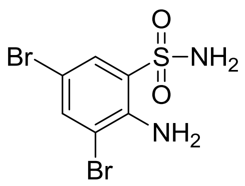

(**2c**)

Pink solid (595.6 mg, 44%)

^{1}H NMR (400 MHz, d_6-DMSO) δ 7.86 (d, J = 2.0 Hz, 1H), 7.72 – 7.66 (m, 3H), 6.03 (s, 2H)

^{13}C NMR (100 MHz, d_6-DMSO) δ 141.59, 137.69, 129.78, 127.01, 110.68, 105.14

HRMS m/z (ESI): calcd. for $[C_6H_6Br_2N_2O_2S\text{-}H]^-$: 326.8444 Found: 326.8468.

Synthesis of 2-aminobenzenesulfonamides bearing an alkyl group

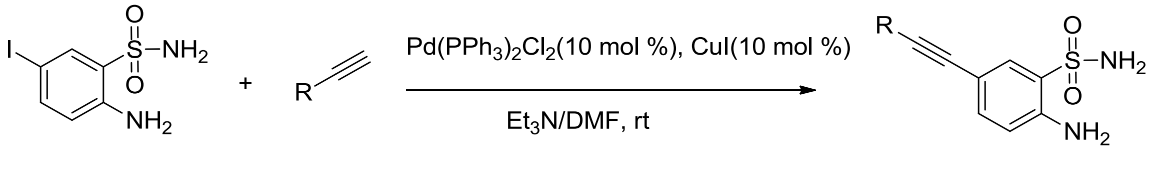

2-Amino-5-iodobenzenesulfonamide (298 mg, 1 mmol), $Pd(PPh_3)_2Cl_2$ (70.2 mg, 0.1 mmol), alkyne (1.5 mmol) and CuI (19 mg, 0.1 mmol) were taken in a 25 mL reaction tube capped with a septum. The reaction tube was evacuated and back-filled with argon. DMF (3 mL) and Et_3N (5 mL) were gas-filled with argon for 30 min, and then pressed into the tube under argon atmosphere. The resulting reaction mixture was stirred at room temperature for 12 h. After disappearance of the reactant (monitored by TLC), then added 50 mL water to the mixture, extracted with CH_2Cl_2 (3 × 30 mL). The combined organic layers were washed with brine (30 mL), dried over anhydrous

Na_2SO_4 and concentrated under reduced pressure. The residue was purified by column chromatography on silica gel to yield the desired products **2d–g.**

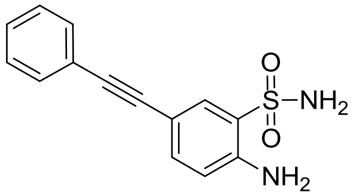

2-Amino-5-(phenylethynyl)benzenesulfonamide (**2d**)

Orange solid (192.1mg, 71%)

^{1}H NMR (400 MHz, d_6-DMSO) δ 7.71 (s, 1H), 7.50 (d, J = 7.7 Hz, 2H), 7.39 (m, 6H), 6.83 (d, J = 8.5 Hz, 1H), 6.28 (s, 2H)

^{13}C NMR (100 MHz, d_6-DMSO) δ 145.79, 135.46, 131.47, 131.07, 128.71, 128.22, 124.13, 122.80, 117.04, 107.93, 89.43, 87.31

HRMS m/z (ESI) : calcd. for $[C_{14}H_{12}N_2O_2S+Na]^+$: 295.0512 Found: 295.0511.

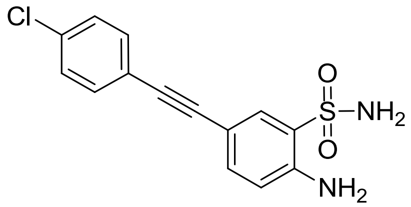

(**2e**)

Yellow solid (275.1mg, 90%)

^{1}H NMR (400 MHz, d_6-DMSO) δ 7.72 (s, 1H), 7.52 (d, J = 8.3 Hz, 2H), 7.45 (d, J = 8.2 Hz, 2H), 7.39 (d, J = 11.9 Hz, 3H), 6.82 (d, J = 8.5 Hz, 1H), 6.30 (s, 2H)

^{13}C NMR (100 MHz, d_6-DMSO) δ 145.95, 135.53, 132.87, 132.73, 131.60, 128.86, 124.15, 121.73, 117.05, 107.62, 90.59, 86.26

HRMS m/z (ESI) : calcd. for $[C_{14}H_{11}ClN_2O_2S+Na]^+$: 329.0122 Found: 329.0119.

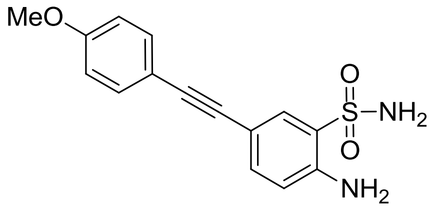

(**2f**)

white solid (250.7mg, 83%)

^{1}H NMR (400 MHz, d_6-DMSO) δ 7.68 (d, J = 1.7 Hz, 1H), 7.44 (d, J = 8.7 Hz, 2H), 7.41 – 7.33

(m, 3H), 6.95 (d, J = 8.7 Hz, 2H), 6.81 (d, J = 8.5 Hz, 1H), 6.23 (s, 2H), 3.78 (s, 3H)

^{13}C NMR (100 MHz, d_6-DMSO) δ 159.18, 145.51, 135.34, 132.61, 131.21, 124.13, 117.04, 114.76, 114.37, 108.48, 87.87, 87.33, 55.27

HRMS m/z (ESI) : calcd. for $[C_{15}H_{14}N_2O_2S+Na]^+$: 325.0617   Found: 325.0638.

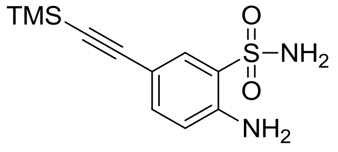

(**2g**)

Brown solid (223.0mg, 82%)

^{1}H NMR (400 MHz, d_6-DMSO) δ 7.58 (s, 1H), 7.30 (s, 2H), 7.25 (d, J = 8.5 Hz, 1H), 6.72 (d, J = 8.5 Hz, 1H), 6.22 (s, 2H), 0.17 (s, 9H)

^{13}C NMR (100 MHz, d_6-DMSO) δ 145.88, 135.52, 131.96, 123.95, 116.86, 107.95, 105.39, 91.49, 0.07

HRMS m/z (ESI) : calcd. for $[C_{11}H_{15}N_2O_2SSi-H]^-$: 267.0629   Found: 267.0629.

Typical procedure for the synthesis of 3-acylbenzothiadiazine 1,1-dioxides

A mixture of acetophenones (0.040 mL, 0.33 mmol), I_2 (0.057 g, 0.225 mmol) and 2-aminobenzenesulfonamides (0.051 g, 0.3 mmol) in DMSO (2 mL) was stirred at 110 °C under air atmosphere in a sealed 50 mL Schlenk tube for 24 h. After the reaction was finished, the reaction mixture was cooled to room temperature. The resulting mixture was taken up by dichloromethane 60 mL and washed with saturated $Na_2S_2O_3$ solution until the brown color disappeared. The organic phases were dried over Na_2SO_4 (anhydrous), concentrated in vacuum, and the resulting residue was purified by column chromatography on silica gel with EtOAc/petroleum (1/4) to afford the product.

Molecular structure and crystallographic data

Crystal data and structure refinement for compound **4b**.

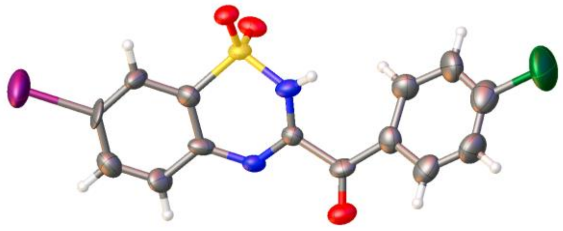

Empirical formula	$C_{14}H_8ClIN_2O_3S$
Formula weight	446.63
Temperature/K	294.39(10)
Crystal system	monoclinic
Space group	Cc
a/Å	7.7317(7)
b/Å	31.7483(14)
c/Å	6.4030(4)
α/°	90
β/°	94.119(6)
γ/°	90
Volume/Å^3	1567.67(18)
Z	4
ρ_{calc}g/cm^3	1.892
μ/mm^{-1}	18.992
F(000)	864.0
Crystal size/mm^3	0.7 × 0.3 × 0.1
Radiation	CuKα (λ = 1.54184)
2Θ range for data collection/°	11.148 to 134.042
Index ranges	$-9 \leq h \leq 9, -37 \leq k \leq 32, -7 \leq l \leq 5$
Reflections collected	8106
Independent reflections	2316 [R_{int} = 0.1527, R_{sigma} = 0.0935]
Data/restraints/parameters	2316/2/199
Goodness-of-fit on F^2	1.046
Final R indexes [I>=2σ (I)]	R_1 = 0.0898, wR_2 = 0.2425
Final R indexes [all data]	R_1 = 0.0953, wR_2 = 0.2599
Largest diff. peak/hole / e Å^{-3}	1.58/-0.87

Spectral data of intermediate D

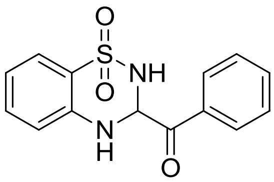

(**D**)

^{1}H NMR (400 MHz, d_6-DMSO) δ 8.18 (d, J = 11.2 Hz, 1H), 8.05 (d, J = 7.7 Hz, 2H), 7.69 (d, J = 7.3 Hz, 1H), 7.59 (t, J = 7.5 Hz, 2H), 7.49 (d, J = 7.9 Hz, 1H), 7.35 (dd, J = 15.2, 6.9 Hz, 2H), 7.16 (d, J = 8.4 Hz, 1H), 6.77 (d, J = 7.5 Hz, 1H), 6.32 (d, J = 11.2 Hz, 1H)

^{13}C NMR (100 MHz, d_6-DMSO) δ191.92, 142.99, 133.89, 133.50, 132.98, 129.25, 128.64, 123.61, 121.39, 116.81, 116.64, 67.03

HRMS m/z (ESI) : calcd. for $[C_{14}H_{12}N_2O_3S+Na]^+$: 311.0461 Found: 311.0456

Monitoring the reaction using ^1H NMR and control experiments

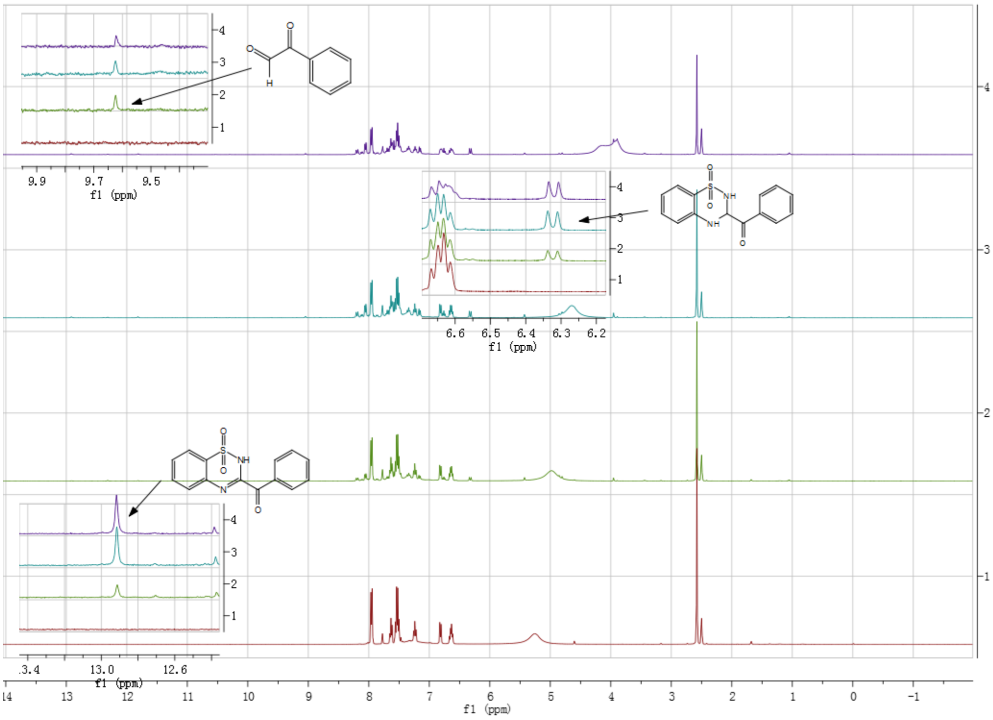

1: 0h   2: 1h   3: 3h   4: 5h

1a (0.33 mmol), **2a** (0.3 mmol), I_2 (0.225 mmol), d_6-DMSO (2 mL), 80°C.

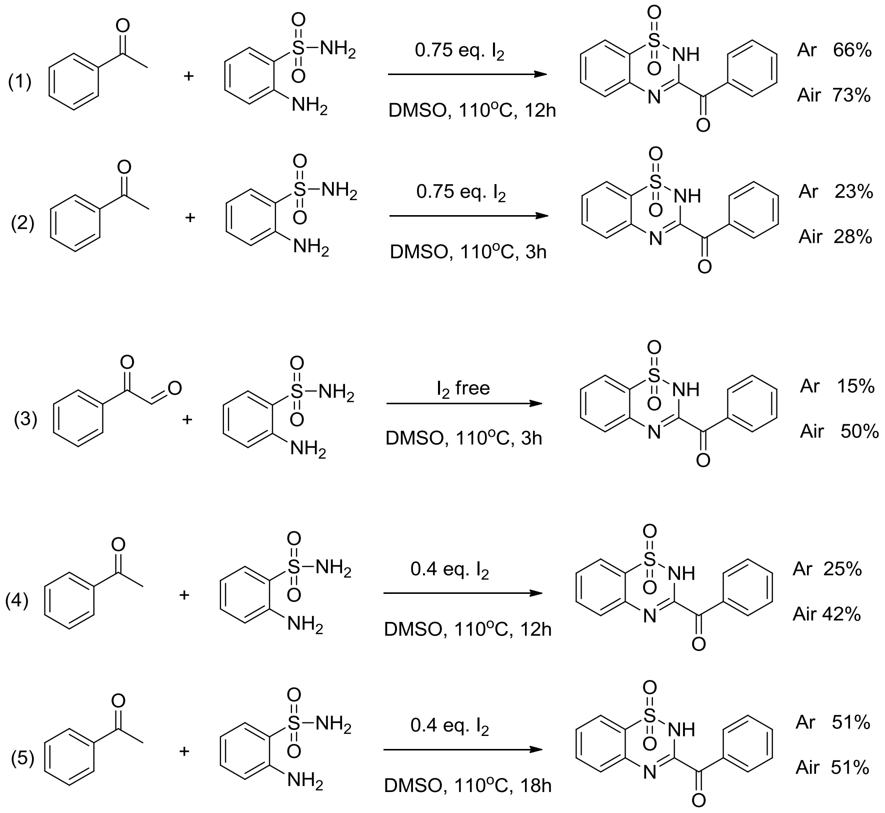

1a or **B** (0.33 mmol), **2a** (0.3 mmol).

A slightly decreased yield was obtained under argon atmosphere within 3h or 12h (eq 1 and 2). In the absence of iodine, an obvious difference was observed using intermediate B as the starting material (eq 3). This result shows oxygen can oxidize intermediate D to the product. We further carried out the reaction using 0.4 equiv of iodine in place of 0.75 equiv of iodine to study the effect of oxygen. After stirring 12h, the product was isolated in 25% (under Ar) and 42% (under air) respectively (eq 4). However, almost the same yields were obtained when the reaction time was extended to 18 hours (eq 5). Based on these experiments, we considered oxygen plays a co-oxidant in this reaction.

Spectral data of compounds

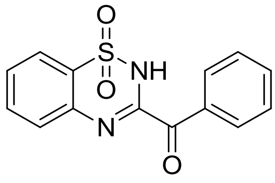

(**3a**)

White solid (68.6 mg, 80%)

^{1}H NMR (400 MHz, d_6-DMSO) δ12.91 (s, 1H), 8.12 (d, J = 7.9 Hz, 2H), 7.92 (d, J = 8.0 Hz, 1H), 7.78 (t, J = 7.0 Hz, 2H), 7.64 (dd, J = 15.2, 7.8 Hz, 3H), 7.57 (t, J = 7.6 Hz, 1H)

^{13}C NMR (100 MHz, d_6-DMSO) δ 186.09, 150.13, 135.03, 134.75, 133.66, 132.78, 130.83, 128.85, 127.51, 123.62, 121.79, 118.86

HRMS m/z (ESI) : calcd. for $[C_{14}H_{10}N_2O_3S\text{-}H]^-$: 285.0339   Found: 285.0334

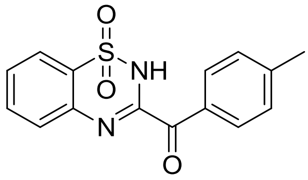

(**3b**)

Yellow solid (67.5 mg, 75%)

1H NMR (400 MHz, d6-DMSO) δ 12.90 (s, 1H), 8.03 (d, J = 8.0 Hz, 2H), 7.92 (d, J = 7.9 Hz, 1H), 7.77 (t, J = 7.7 Hz, 1H), 7.63 (d, J = 8.2 Hz, 1H), 7.56 (t, J = 7.6 Hz, 1H), 7.44 (d, J = 7.7 Hz, 2H), 2.43 (s, 3H)

13C NMR (100 MHz, d6-DMSO) δ 185.46, 150.42, 146.13, 134.69, 133.58, 130.89, 130.13, 129.47, 127.41, 123.57, 121.78, 118.73, 21.45

HRMS m/z (ESI) : calcd. for [C15H12N2O3S-H]-: 299.0496   Found: 299.0487

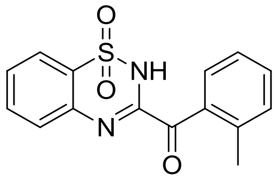

(**3c**)

White solid (69.2 mg, 77%)

^{1}H NMR (400 MHz, d_6-DMSO) δ 12.91 (s, 1H), 7.91 (d, J = 7.9 Hz, 1H), 7.79 (dd, J = 14.6, 7.5 Hz, 2H), 7.70 (d, J = 8.3 Hz, 1H), 7.57 (q, J = 8.2 Hz, 2H), 7.45 – 7.38 (m, 2H), 2.47 (d, J = 4.0 Hz, 3H)

^{13}C NMR (100 MHz, d_6-DMSO) δ 188.97, 150.14, 139.26, 134.72, 133.69, 133.20, 132.77, 131.87, 131.62, 127.55, 125.70, 123.63, 121.65, 118.96, 20.36

HRMS m/z (ESI) : calcd. for $[C_{15}H_{12}N_2O_3S\text{-}H]^-$: 299.0496   Found: 299.0491

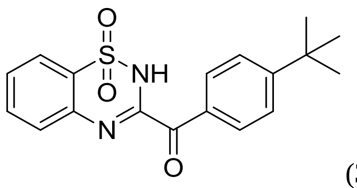

(**3d**)

White solid (79.0 mg, 77%)

^{1}H NMR (400 MHz, d_6-DMSO) δ 12.85 (s, 1H), 8.02 (d, J = 8.3 Hz, 2H), 7.88 (d, J = 7.9 Hz, 1H), 7.73 (d, J = 7.7 Hz, 1H), 7.62 (dd, J = 7.7, 5.0 Hz, 3H), 7.53 (t, J = 7.6 Hz, 1H), 1.29 (s, 9H)

^{13}C NMR (100 MHz, d_6-DMSO) δ 185.53, 158.51, 150.27, 134.69, 133.60, 130.81, 130.17, 127.44, 125.77, 123.58, 121.77, 118.76, 35.18, 30.67

HRMS m/z (ESI) : calcd. for $[C_{18}H_{18}N_2O_3S\text{-}H]^-$: 341.0965   Found: 341.0958

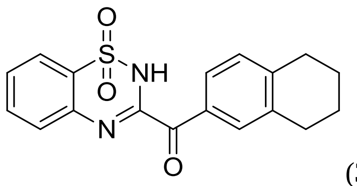

(**3e**)

White solid (70.4 mg, 69%)

^{1}H NMR (400 MHz, d_6-DMSO) δ 12.86 (s, 1H), 7.88 (d, J = 7.9 Hz, 1H), 7.76 (t, J = 12.2 Hz, 3H), 7.58 (d, J = 8.2 Hz, 1H), 7.52 (s, 1H), 7.27 (d, J = 7.5 Hz, 1H), 2.77 (d, J = 6.1 Hz, 4H), 1.73 (s, 4H)

^{13}C NMR (100 MHz, d_6-DMSO) δ 186.09, 151.07, 145.84, 137.90, 135.11, 134.00, 131.67, 130.51, 129.90, 128.25, 127.83, 123.99, 122.22, 119.11, 29.64, 29.11, 22.73, 22.55

HRMS m/z (ESI) : calcd. for $[C_{18}H_{16}N_2O_3S\text{-}H]^-$: 339.0809   Found: 339.0817

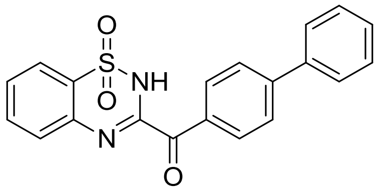

(**3f**)

White solid (69.5 mg, 64%)

^{1}H NMR (400 MHz, d_6-DMSO) δ 12.93 (s, 1H), 8.23 (d, J = 8.3 Hz, 2H), 7.94 (d, J = 8.2 Hz, 3H), 7.80 (t, J = 6.3 Hz, 3H), 7.69 (d, J = 8.2 Hz, 1H), 7.60 – 7.50 (m, 3H), 7.47 (q, J = 7.5 Hz, 1H)

^{13}C NMR (100 MHz, d_6-DMSO) δ 185.45, 150.11, 146.23, 138.53, 134.76, 133.63, 131.62, 131.57, 129.20, 128.87, 127.48, 127.20, 126.95, 123.60, 121.78, 118.86

HRMS m/z (ESI) : calcd. for $[C_{20}H_{14}N_2O_3S\text{-}H]^-$: 361.0652   Found: 361.0668

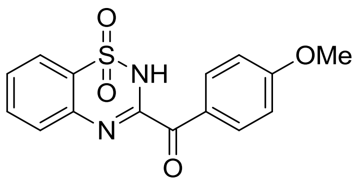

(**3g**)

Green solid (57.8 mg, 61%)

^{1}H NMR (400 MHz, d_6-DMSO) δ 12.88 (s, 1H), 8.13 (d, J = 8.8 Hz, 2H), 7.92 (d, J = 8.0 Hz, 1H), 7.77 (s, 1H), 7.61 (d, J = 8.2 Hz, 1H), 7.56 (s, 1H), 7.17 (d, J = 8.3 Hz, 2H), 3.90 (s, 3H)

^{13}C NMR (100 MHz, d_6-DMSO) δ 184.06, 164.87, 150.81, 134.72, 133.59, 133.42, 127.40, 125.25, 123.58, 121.79, 118.69, 114.45, 55.91

HRMS m/z (ESI) : calcd. for $[C_{15}H_{12}N_2O_4S-H]^-$: 315.0445 Found: 315.0426

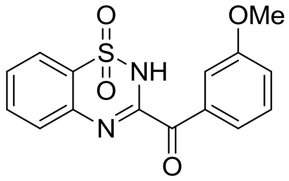

(**3h**)

Yellow solid (52.1 mg, 55%)

^{1}H NMR (400 MHz, d_6-DMSO) δ 12.91 (s, 1H), 7.92 (d, J = 7.9 Hz, 1H), 7.77 (t, J = 7.7 Hz, 1H), 7.66 (dd, J = 19.1, 10.8 Hz, 3H), 7.59 – 7.52 (m, 2H), 7.37 (d, J = 7.9 Hz, 1H), 3.84 (s, 3H)

^{13}C NMR (100 MHz, d_6-DMSO) δ 185.81, 159.18, 150.12, 134.74, 133.95, 133.61, 130.07, 127.46, 123.58, 123.40, 121.80, 120.98, 118.82, 115.19, 55.52

HRMS m/z (ESI) : calcd. for $[C_{15}H_{12}N_2O_4S-H]^-$: 315.0445 Found: 315.0451

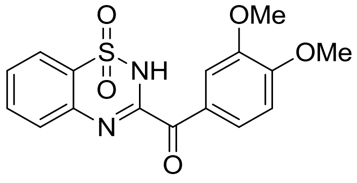

(**3i**)

Yellow solid (62.3 mg, 60%)

^{1}H NMR (400 MHz, d_6-DMSO) δ 12.91 (s, 1H), 7.92 (d, J = 7.4 Hz, 1H), 7.85 – 7.72 (m, 2H), 7.59 (dd, J = 18.2, 8.7 Hz, 3H), 7.20 (d, J = 7.8 Hz, 1H), 3.91 (s, 3H), 3.85 (s, 3H)

^{13}C NMR (100 MHz, d_6-DMSO) δ 184.07, 155.07, 151.06, 148.85, 134.74, 133.59, 127.40, 127.04, 125.13, 123.60, 121.86, 118.63, 111.74, 111.21, 56.11, 55.67

HRMS m/z (ESI) : calcd. for $[C_{16}H_{14}N_2O_5S-H]^-$: 345.0551 Found: 345.0551

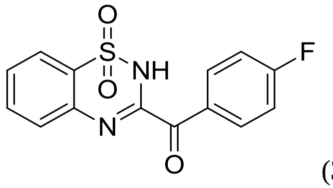

(**3j**)

White solid (47.4 mg, 52%)

^{1}H NMR (400 MHz, d_6-DMSO) δ 12.87 (s, 1H), 8.24 (dd, J = 8.7, 5.6 Hz, 2H), 7.92 (d, J = 7.9 Hz, 1H), 7.77 (t, J = 7.8 Hz, 1H), 7.69 (d, J = 8.2 Hz, 1H), 7.56 (t, J = 7.6 Hz, 1H), 7.47 (t, J = 8.8 Hz, 2H)

^{13}C NMR (100 MHz, d_6-DMSO) δ184.58, 165.95(J = 253 Hz), 149.67, 134.73, 134.18(J = 10 Hz)，133.62, 129.55 (J = 2.8 Hz), 127.47, 123.58, 121.70, 118.89, 116.02（J = 22Hz)

HRMS m/z (ESI) : calcd. for $[C_{14}H_9FN_2O_3S\text{-}H]^-$: 303.0245 Found: 303.0226

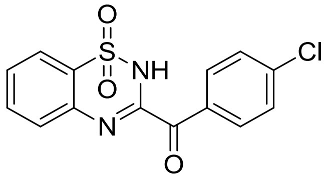

(**3k**)

Yellow solid (63.5 mg, 56%)

^{1}H NMR (400 MHz, d_6-DMSO) δ 12.86 (s, 1H), 8.15 (d, J = 8.5 Hz, 2H), 7.91 (d, J = 8.0 Hz, 1H), 7.77 (t, J = 7.5 Hz, 1H), 7.70 (dd, J = 8.1, 4.2 Hz, 3H), 7.56 (t, J = 7.5 Hz, 1H)

^{13}C NMR (100 MHz, d_6-DMSO) δ 185.04, 149.45, 139.79, 134.83, 133.59, 132.7, 131.65, 128.85, 127.42, 123.54, 121.69, 118.97

HRMS m/z (ESI) : calcd. for $[C_{14}H_9ClN_2O_3S\text{-}H]^-$: 318.9950 Found: 318.9964

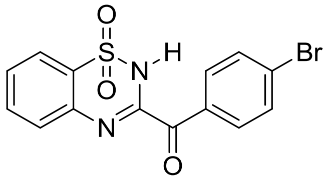

(**3l**)

Yellow solid (79.7 mg, 73%)

^{1}H NMR (400 MHz, d_6-DMSO) δ 12.85 (s, 1H), 8.07 (d, J = 8.2 Hz, 2H), 7.92 (d, J = 8.0 Hz, 1H), 7.85 (d, J = 8.1 Hz, 2H), 7.77 (t, J = 7.7 Hz, 1H), 7.70 (d, J = 8.3 Hz, 1H), 7.56 (t, J = 7.6 Hz, 1H)
^{13}C NMR (100 MHz, d_6-DMSO) δ 185.25, 149.35, 134.72, 133.65, 132.81, 131.98, 131.83, 129.21, 127.50, 123.58, 121.66, 118.94

HRMS m/z (ESI) : calcd. for $[C_{14}H_9BrN_2O_3S\text{-}H]^-$: 362.9444 Found: 362.9443

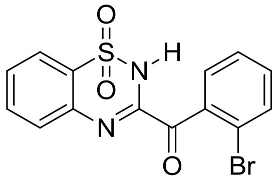

(**3m**)

Yellow Solid (52.4 mg, 48%)

^{1}H NMR (400 MHz, d_6-DMSO) δ 13.02 (s, 1H), 7.89 (d, J = 8.0 Hz, 1H), 7.83-7.74 (m, 4H),

7.63-7.53 (m, 3H)

^{13}C NMR (100 MHz, d_6-DMSO) δ 189.07, 148.16, 136.10, 134.52, 133.75, 133.59, 132.95, 131.24, 127.69, 123.61, 121.58, 119.71, 119.04

HRMS m/z (ESI) : calcd. for $[C_{14}H_9BrN_2O_3S-H]^-$: 362.9444   Found: 362.9433

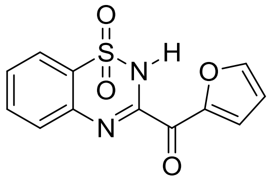

(**3n**)

Yellow Solid (53.0 mg, 64%)

^{1}H NMR (400 MHz, d_6-DMSO) δ 12.76 (s, 1H), 8.28 (s, 1H), 8.03 (d, J = 3.7 Hz, 1H), 7.91 (d, J = 7.9 Hz, 1H), 7.80 – 7.72 (m, 2H), 7.60 - 7.52 (m, 1H), 6.89 (s, 1H)

^{13}C NMR (100 MHz, d_6-DMSO) δ 171.77, 151.62, 148.82, 148.58, 134.93, 134.01, 127.95, 127.08, 123.92, 121.99, 119.50, 114.11

HRMS m/z (ESI) : calcd. for $[C_{12}H_8N_2O_4S+Na]^+$: 299.0097   Found: 299.0106

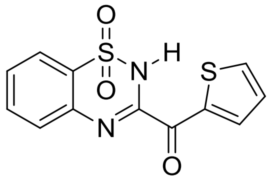

(**3o**)

Yellow Solid (37.7 mg, 43%)

^{1}H NMR (400 MHz, d_6-DMSO) δ 12.75 (s, 1H), 8.33 (d, J = 3.6 Hz, 1H), 8.26 (d, J = 4.4 Hz, 1H), 7.88 (d, J = 7.9 Hz, 1H), 7.73 (d, J = 3.6 Hz, 2H), 7.52 (dd, J = 7.7, 4.8 Hz, 1H), 7.33 (t, J = 4.0 Hz, 1H)

^{13}C NMR (100 MHz, d_6-DMSO) δ 176.69, 148.46, 140.04, 138.75, 136.97, 134.56, 133.62, 129.00, 127.55, 123.52, 121.63, 119.14

HRMS m/z (ESI) : calcd. for $[C_{12}H_8N_2O_3S_2+Na]^+$: 314.9869   Found: 314.9871

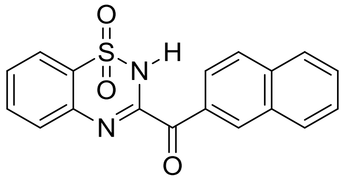

(**3p**)

Yellow solid (74.6 mg, 74%)

^{1}H NMR (400 MHz, d_6-DMSO) δ 13.00 (br s, 1H), 8.81 (s, 1H), 8.16 (d, J = 8.2 Hz, 1H), 8.11 (d, J = 9.6 Hz, 2H), 8.06 (d, J = 8.2 Hz, 1H), 7.96 (d, J = 7.9 Hz, 1H), 7.82 – 7.73 (m, 2H), 7.69 – 7.64 (m, 2H), 7.58 (t, J = 7.7 Hz, 1H)

^{13}C NMR (100 MHz, d_6-DMSO) δ 186.02, 150.52, 135.69, 134.81, 133.85, 133.61, 131.68,

130.14, 130.02, 129.86 128.57, 127.88, 127.45, 124.95, 123.62, 121.90, 118.82

HRMS m/z (ESI) : calcd. for $[C_{18}H_{12}N_2O_3S\text{-}H]^-$: 335.0496   Found: 335.0478

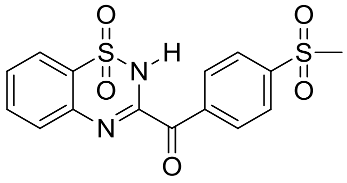

(**3q**)

White solid (71.0 mg, 65%)

^{1}H NMR (400 MHz, d_6-DMSO) δ 12.85 (s, 1H), 8.30 (t, J = 7.1 Hz, 2H), 8.15 (d, J = 8.2 Hz, 2H), 7.95 – 7.88 (m, 1H), 7.80 – 7.74 (m, 2H), 7.57 (t, J = 6.3 Hz, 1H), 3.34 (s, 3H)

^{13}C NMR (100 MHz, d_6-DMSO) δ 185.79, 148.71, 144.97, 137.30, 134.77, 133.76, 131.87, 127.62, 126.96, 123.64, 121.62, 119.10, 43.22

HRMS m/z (ESI) : calcd. for $[C_{15}H_{12}N_2O_5S_2\text{-}H]^-$: 363.0115   Found:363.0115

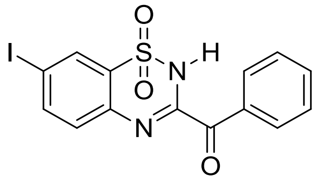

(**4a**)

White solid (90.2 mg, 73%)

^{1}H NMR (400 MHz, d_6-DMSO) δ 12.98 (s, 1H), 8.17 (s, 1H), 8.11 (t, J = 6.6 Hz, 3H), 7.79 (t, J = 7.3 Hz, 1H), 7.63 (t, J = 7.6 Hz, 2H), 7.47 (d, J = 8.7 Hz, 1H)

^{13}C NMR (100 MHz, d_6-DMSO) δ 185.87, 149.86, 142.04, 134.96, 134.28, 132.70, 131.18, 130.86, 128.76, 123.05, 121.04, 90.86

HRMS m/z (ESI): calcd. for $[C_{14}H_9IN_2O_3S\text{-}H]^-$: 410.9306   Found: 410.9322

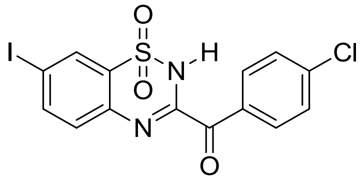

(**4b**)

White solid (92.3 mg, 69%)

^{1}H NMR (400 MHz, d_6-DMSO) δ 12.92 (s, 1H), 8.20 – 8.12 (m, 3H), 8.10 (d, J = 8.7 Hz, 1H), 7.70 (d, J = 8.3 Hz, 2H), 7.50 (d, J = 8.7 Hz, 1H)

^{13}C NMR (100 MHz, d_6-DMSO) δ 184.85, 149.27, 142.03, 139.78, 134.39, 132.81, 131.61, 131.16, 128.81, 122.99, 121.18, 90.84

HRMS m/z (ESI) : calcd. for $[C_{14}H_8ClIN_2O_3S\text{-}H]^-$: 444.8916   Found: 444.8910

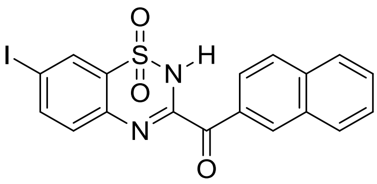

(**4c**)

Yellow solid (87.3 mg, 63%)

^{1}H NMR (400 MHz, d_6-DMSO) δ 13.07(br s, 1H), 8.80 (s, 1H), 8.19 (s, 1H), 8.12 (q, J = 8.4 Hz, 4H), 8.05 (d, J = 8.2 Hz, 1H), 7.74 (t, J = 7.5 Hz, 1H), 7.66 (t, J = 7.5 Hz, 1H), 7.47 (d, J = 8.7 Hz, 1H)

^{13}C NMR (100 MHz, d_6-DMSO) δ 186.02, 150.67, 141.94, 135.67, 134.79, 133.86, 131.66, 131.20, 130.16, 130.00, 129.84 128.52, 127.88, 127.44, 124.99, 123.32, 121.29, 90.66

HRMS m/z (ESI) : calcd. for $[C_{18}H_{11}IN_2O_3S\text{-}H]^-$: 460.9462 Found: 460.9436

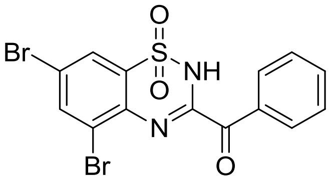

(**4d**)

Yellow solid (88.8 mg, 67%)

^{1}H NMR (400 MHz, d_6-DMSO) δ 11.88 (br s, 1H), 8.38 (d, J = 0.9 Hz, 1H), 8.16 (s, 1H), 8.03 (d, J = 7.7 Hz, 2H), 7.79 (t, J = 7.4 Hz, 1H), 7.64 (t, J = 7.7 Hz, 2H)

^{13}C NMR (100 MHz, d_6-DMSO) δ 185.07, 153.20, 139.10, 135.16, 132.84, 130.28, 129.11, 125.60, 124.37, 118.54, 113.40

HRMS m/z (ESI) : calcd. for $[C_{14}H_8Br_2N_2O_3S\text{-}H]^-$: 440.8550 Found: 440.8534

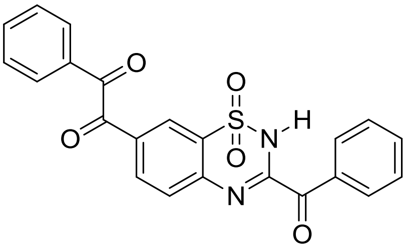

(**4e**)

Orange solid (80.2 mg, 64%)

^{1}H NMR (400 MHz, d_6-DMSO) δ 13.29 (s, 1H), 8.36 (s, 1H), 8.30 (dd, J = 8.7, 1.6 Hz, 1H), 8.17 – 8.11 (m, 2H), 8.02 (d, J = 7.4 Hz, 2H), 7.87 (d, J = 8.7 Hz, 1H), 7.81 (dd, J = 18.4, 7.9 Hz, 2H), 7.64 (dd, J = 13.2, 7.1 Hz, 4H)

^{13}C NMR (100 MHz, d_6-DMSO) δ 193.12, 191.10, 185.72, 135.62, 135.02, 133.83, 132.62, 132.12, 130.91, 130.32, 130.06, 129.37, 128.77, 126.34, 121.71, 120.25

HRMS m/z (ESI) : calcd. for $[C_{22}H_{14}N_2O_5S\text{-}H]^-$: 417.0540 Found: 417.0533

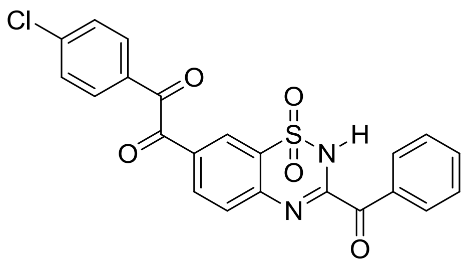

(**4f**)

Yellow solid (66.4 mg, 49%)

^{1}H NMR (400 MHz, d_6-DMSO) δ 13.27 (s, 1H), 8.41 (s, 1H), 8.31 (d, J = 7.5 Hz, 1H), 8.09 (dd, J = 40.2, 5.9 Hz, 4H), 7.86 (d, J = 7.6 Hz, 1H), 7.79 (s, 1H), 7.74 – 7.60 (m, 4H)

^{13}C NMR (100 MHz, d_6-DMSO) δ 191.40, 190.13, 185.73, 150.56, 140.46, 135.00, 133.94, 132.64, 132.00, 130.92, 130.28, 129.41, 128.76, 126.74, 121.62, 120.10

HRMS m/z (ESI) : calcd. for $[C_{22}H_{13}ClN_2O_5S\text{-}H]^-$:451.0150   Found: 451.0136

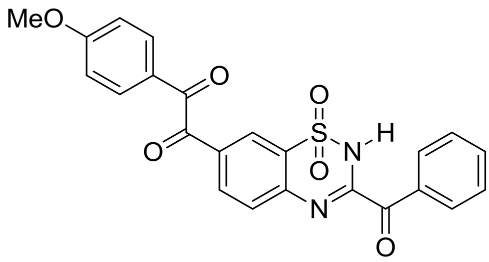

(**4g**)

Yellow solid (55.1 mg, 41%)

^{1}H NMR (400 MHz, d_6-DMSO) δ 13.24 (s, 1H), 8.25 (d, J = 13.7 Hz, 2H), 8.11 (d, J = 7.6 Hz, 2H), 7.95 (d, J = 8.4 Hz, 2H), 7.83 (d, J = 8.6 Hz, 1H), 7.76 (t, J = 7.3 Hz, 1H), 7.60 (t, J = 7.5 Hz, 2H), 7.13 (d, J = 8.4 Hz, 2H), 3.86 (s, 3H)

^{13}C NMR (100 MHz, d_6-DMSO) δ 191.78, 191.61, 185.82, 165.17, 150.67, 139.98, 135.02, 133.71, 132.66, 130.96, 130.49, 128.79, 126.14, 124.97, 121.73, 120.39, 114.90, 55.96

HRMS m/z (ESI) : calcd. for $[C_{22}H_{16}ClN_2O_6S\text{-}H]^-$:447.0645   Found: 447.0619

Copies of ^{1}H and ^{13}C NMR spectra

^{1}H NMR of compound 2b

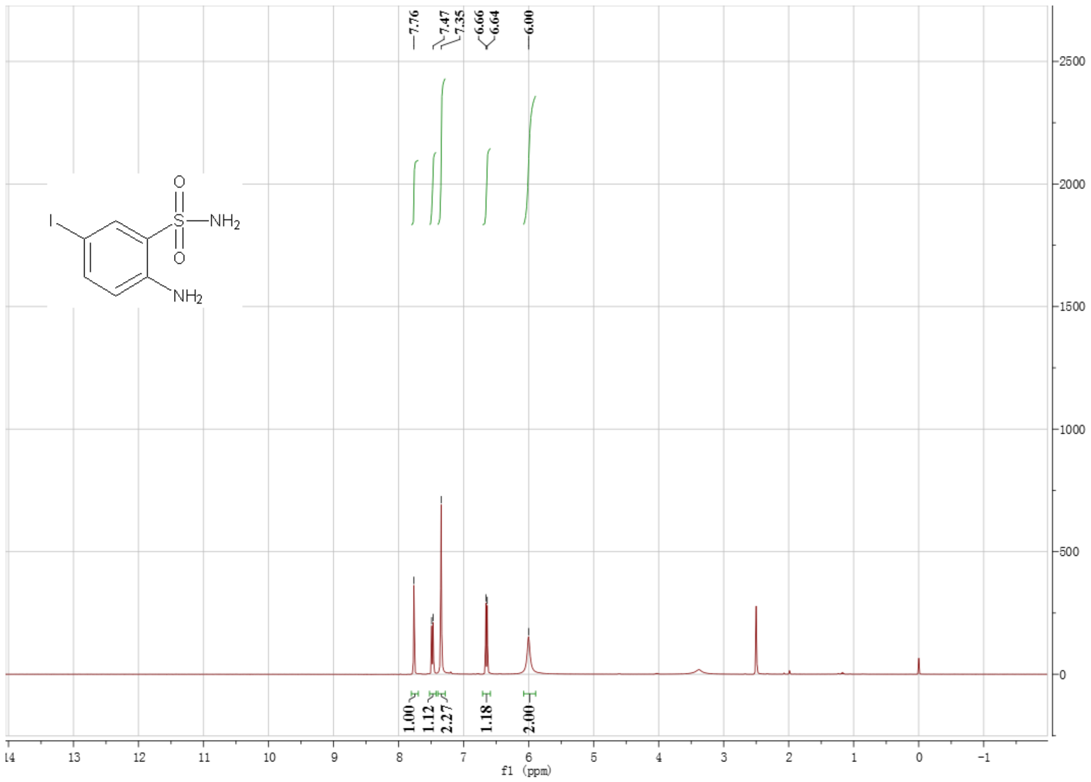

^{13}C NMR of compound 2b

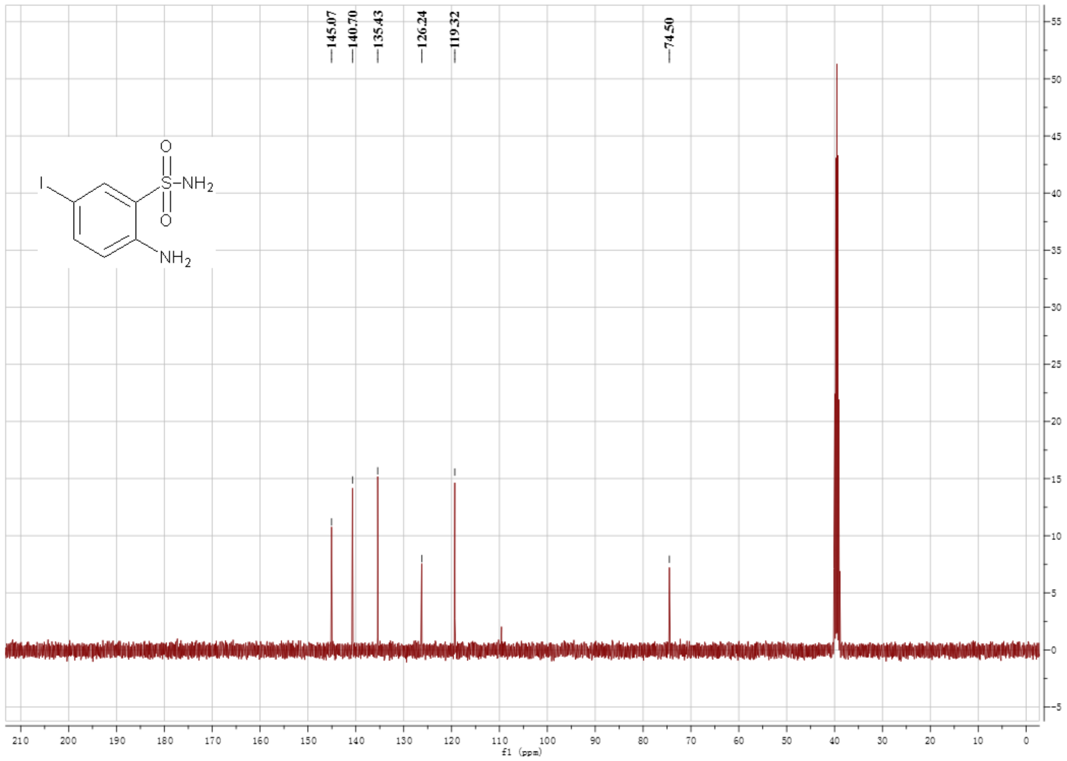

^{1}H NMR of compound 2c

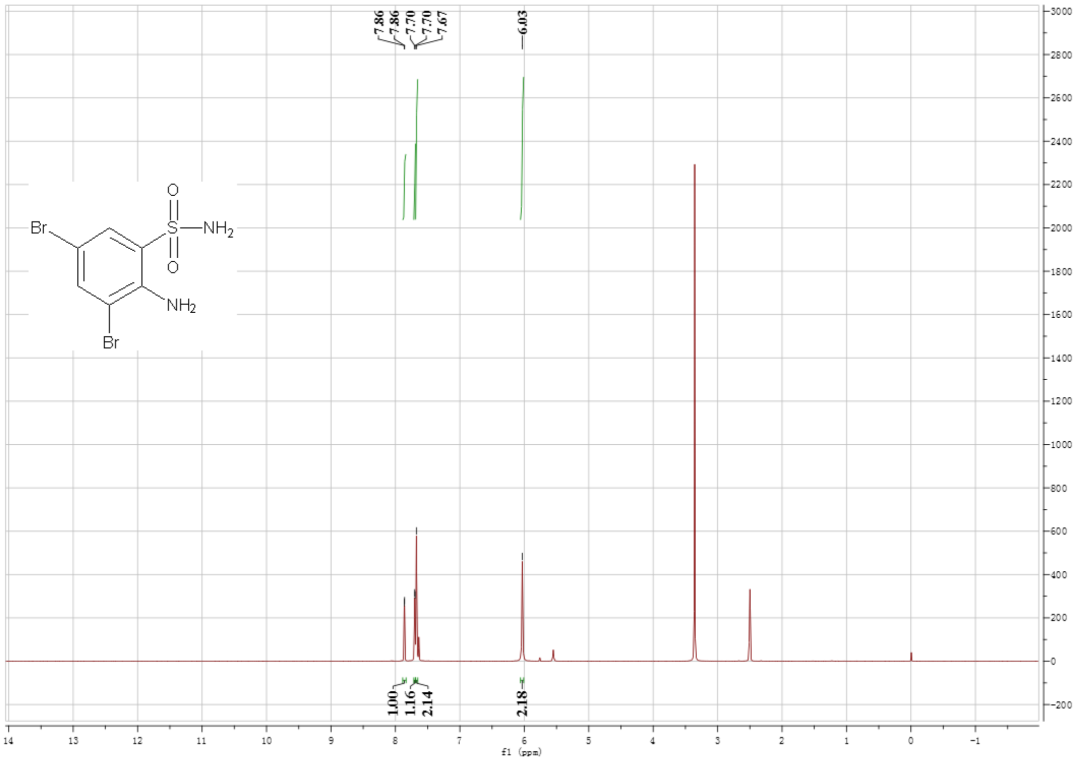

^{1}H NMR of compound 2c

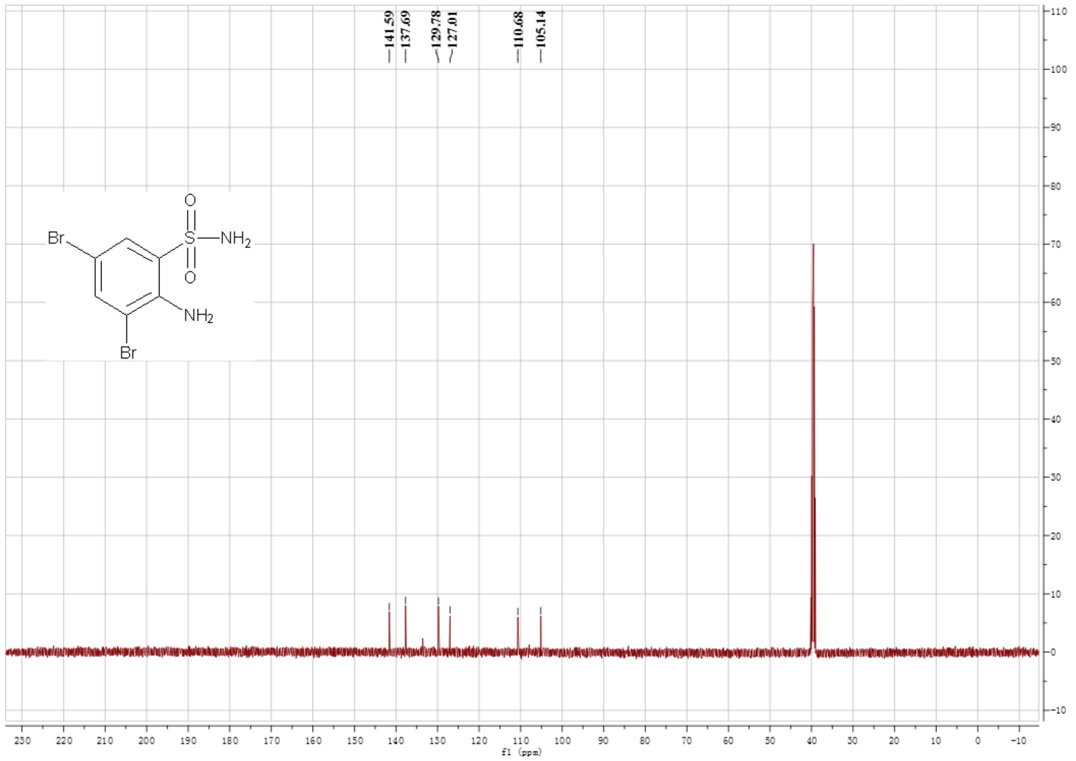

^{1}H NMR of compound 2d

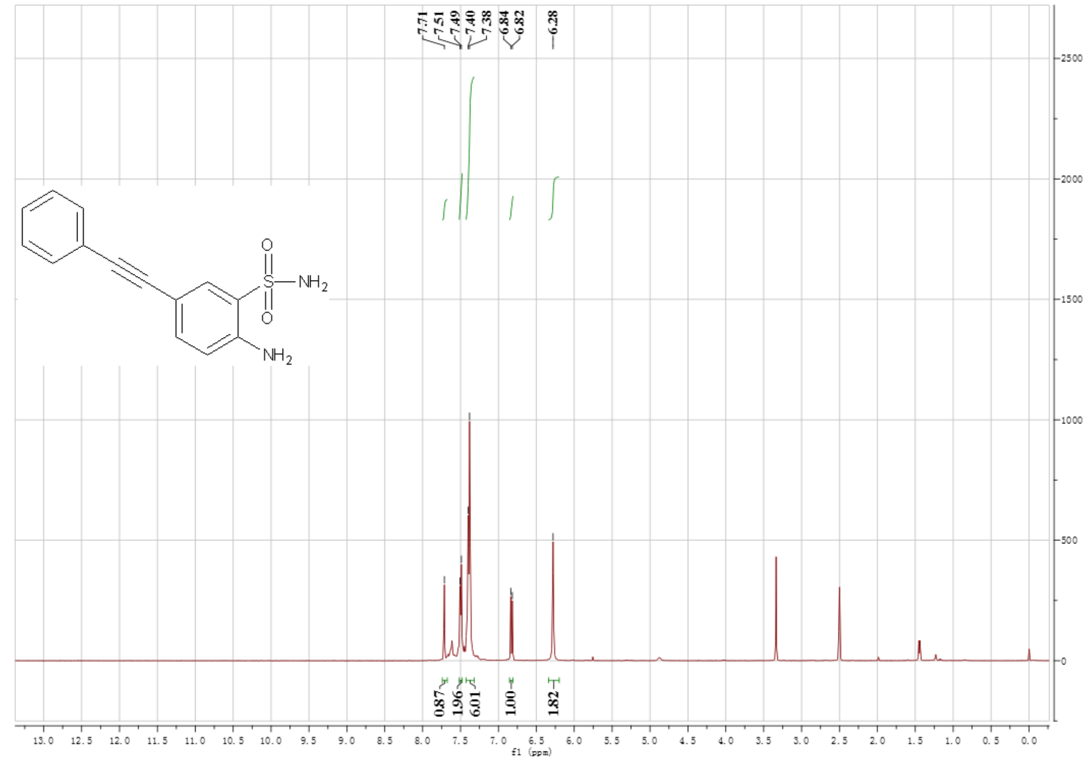

^{13}C NMR of compound 2d

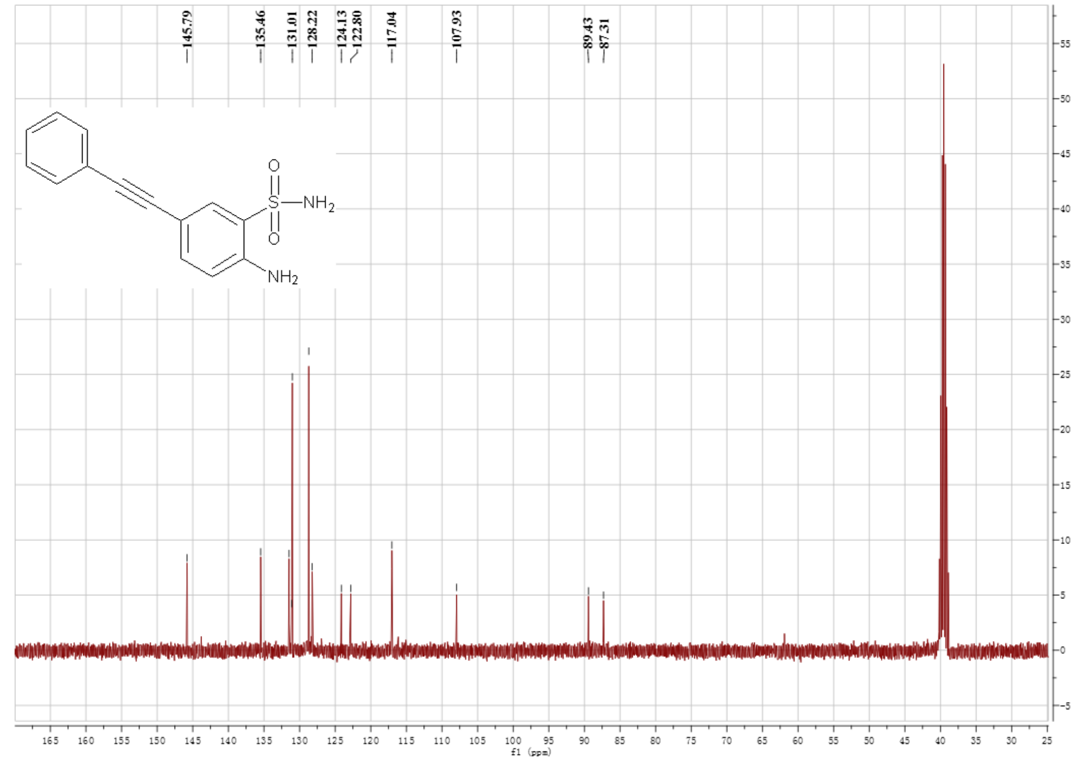

^{1}H NMR of compound 2e

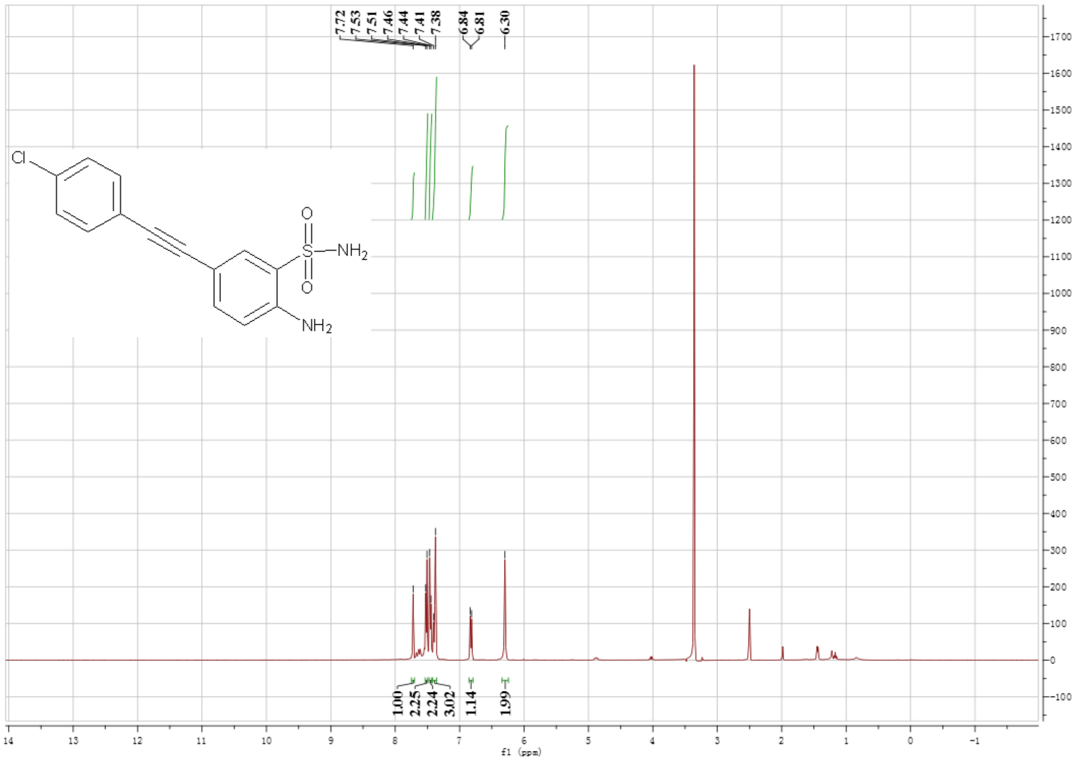

^{13}C NMR of compound 2e

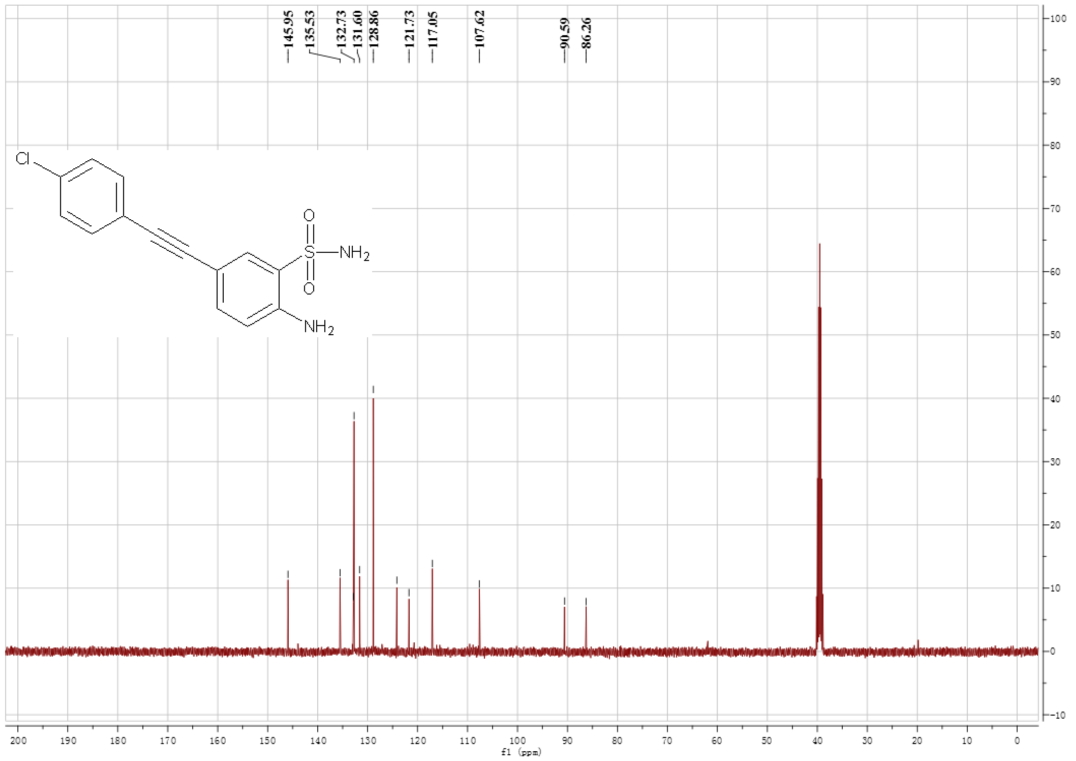

^{1}H NMR of compound 2f

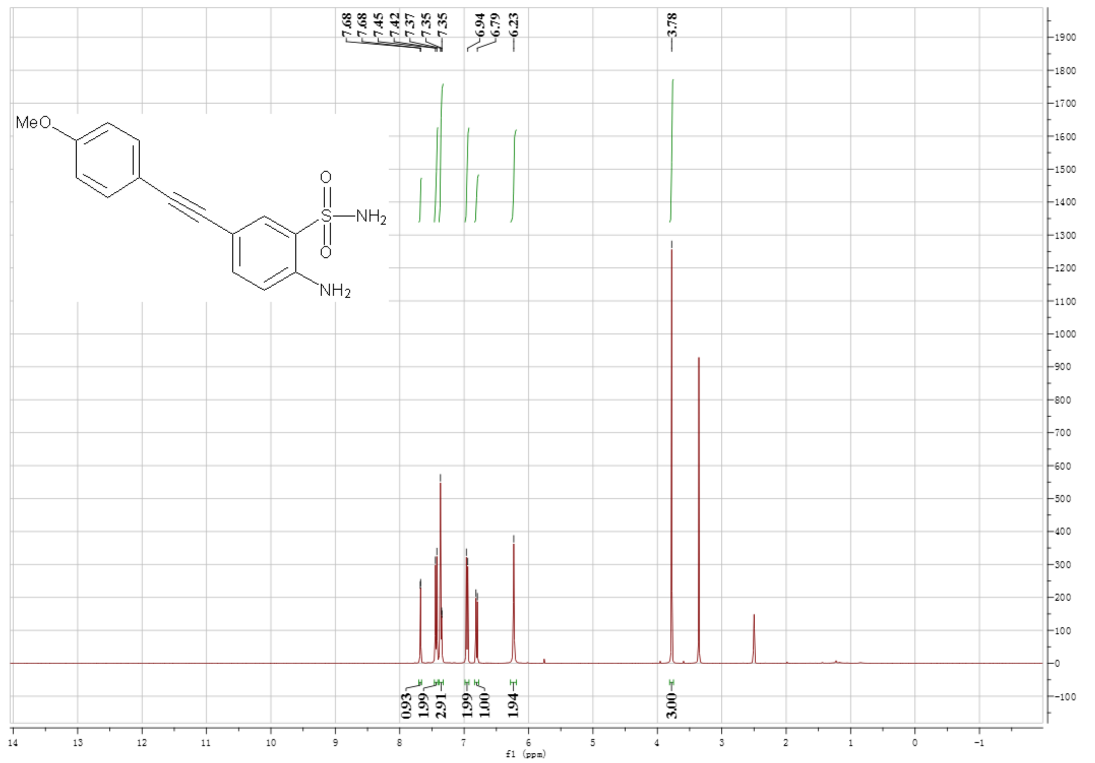

^{13}C NMR of compound 2f

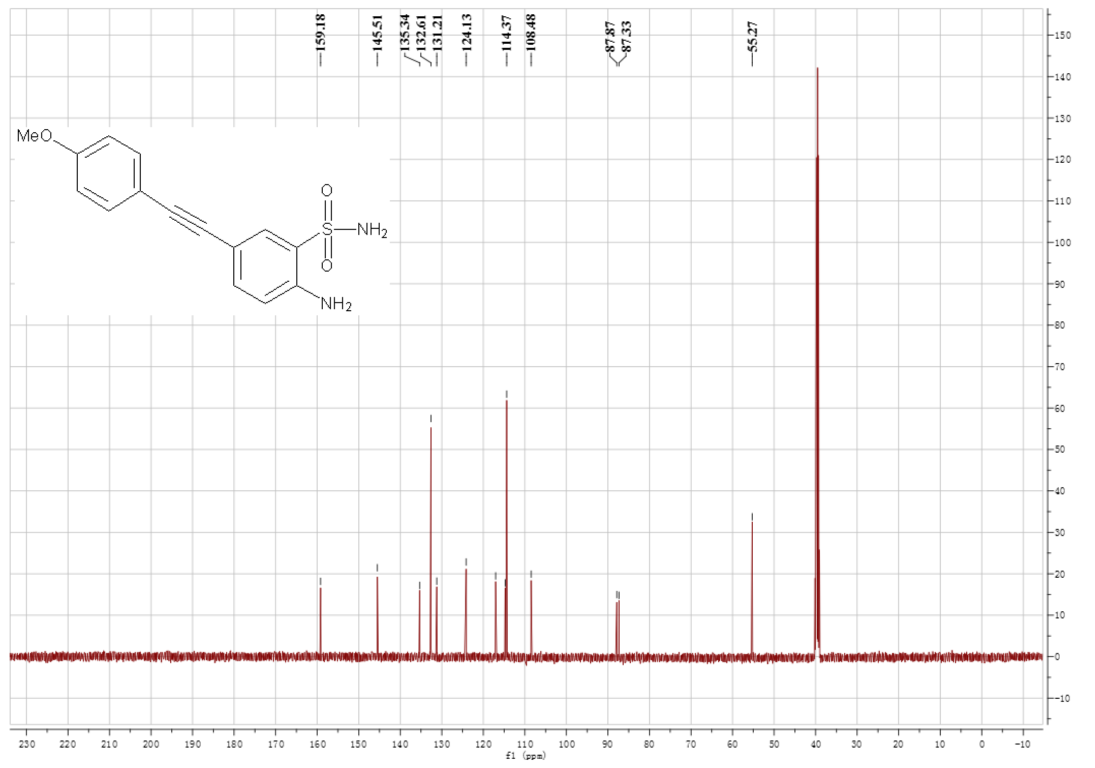

^{1}H NMR of compound 2g

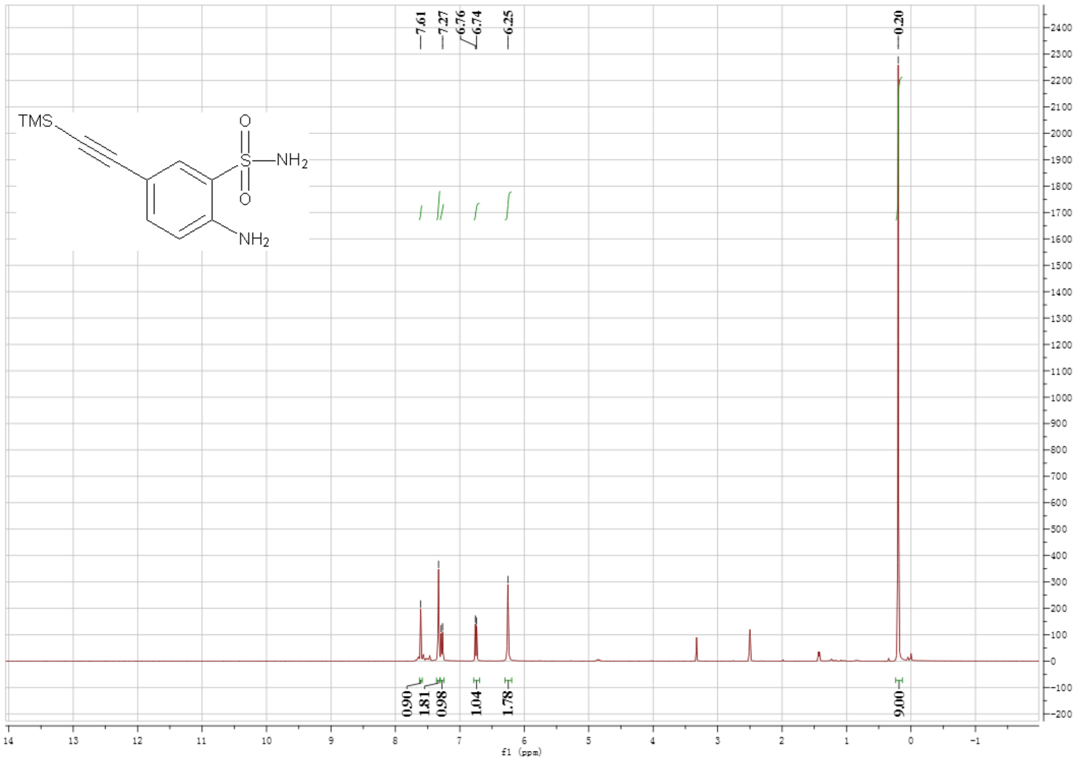

^{13}C NMR of compound 2g

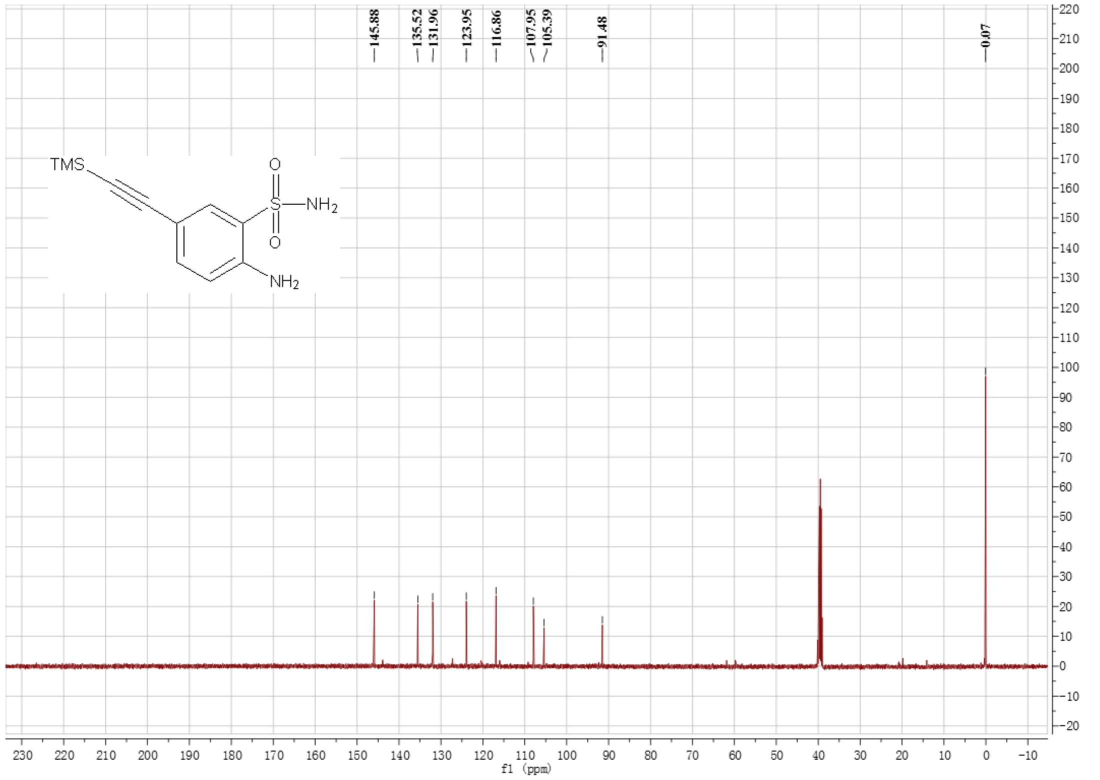

^{1}H NMR of intermediate D

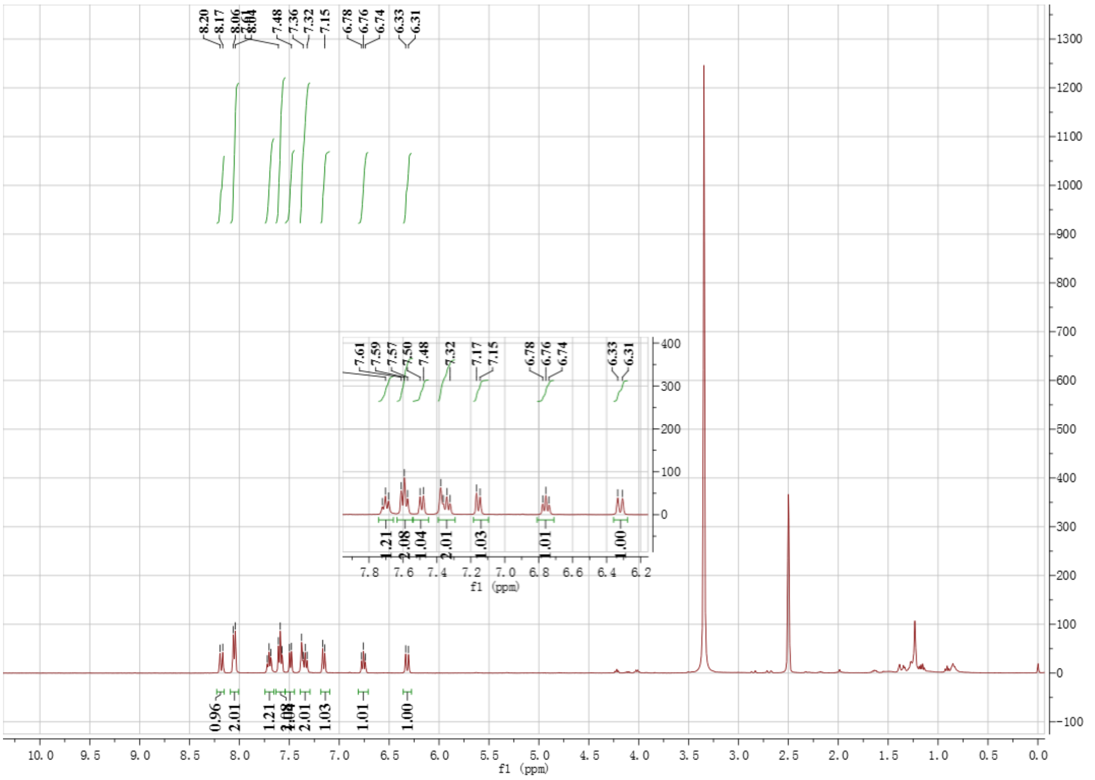

^{13}C NMR of intermediate D

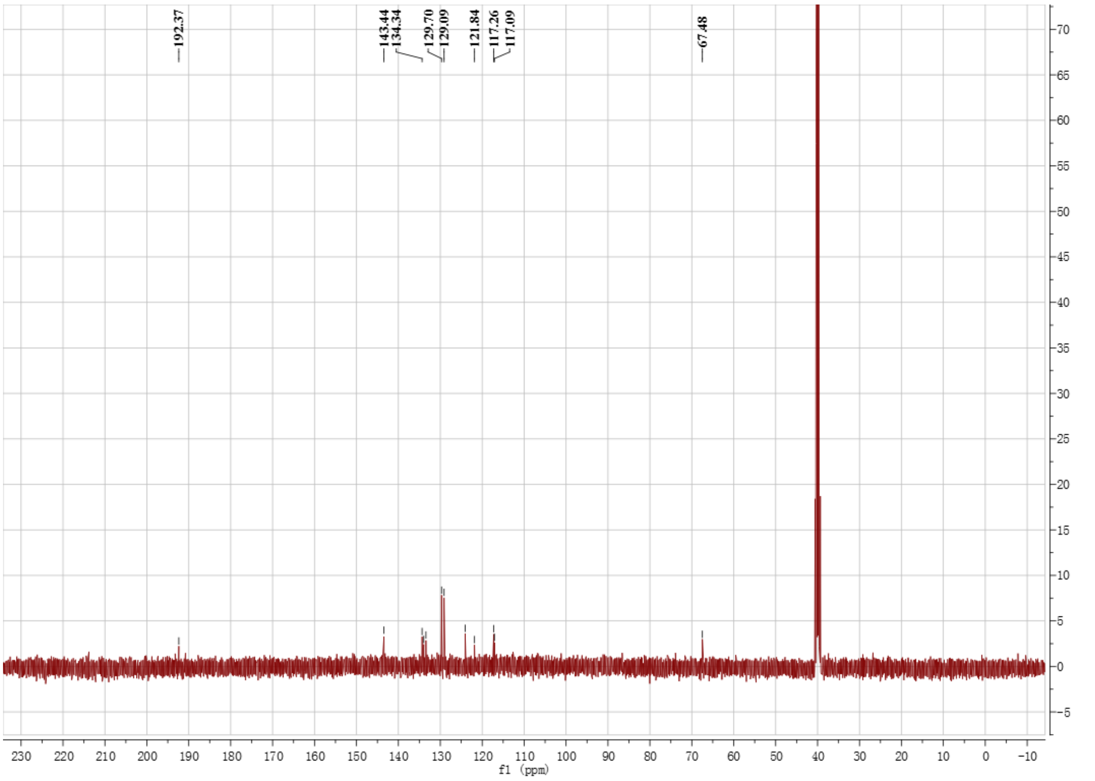

^{1}H NMR of compound 3a

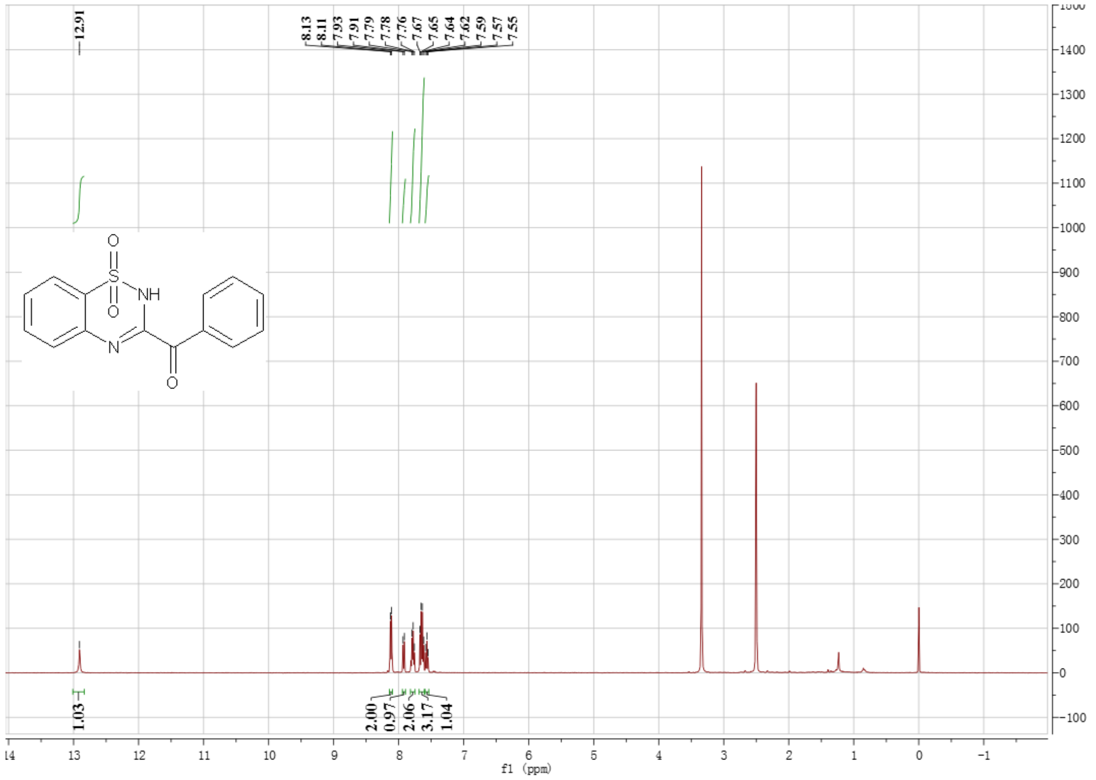

^{13}C NMR of compound 3a

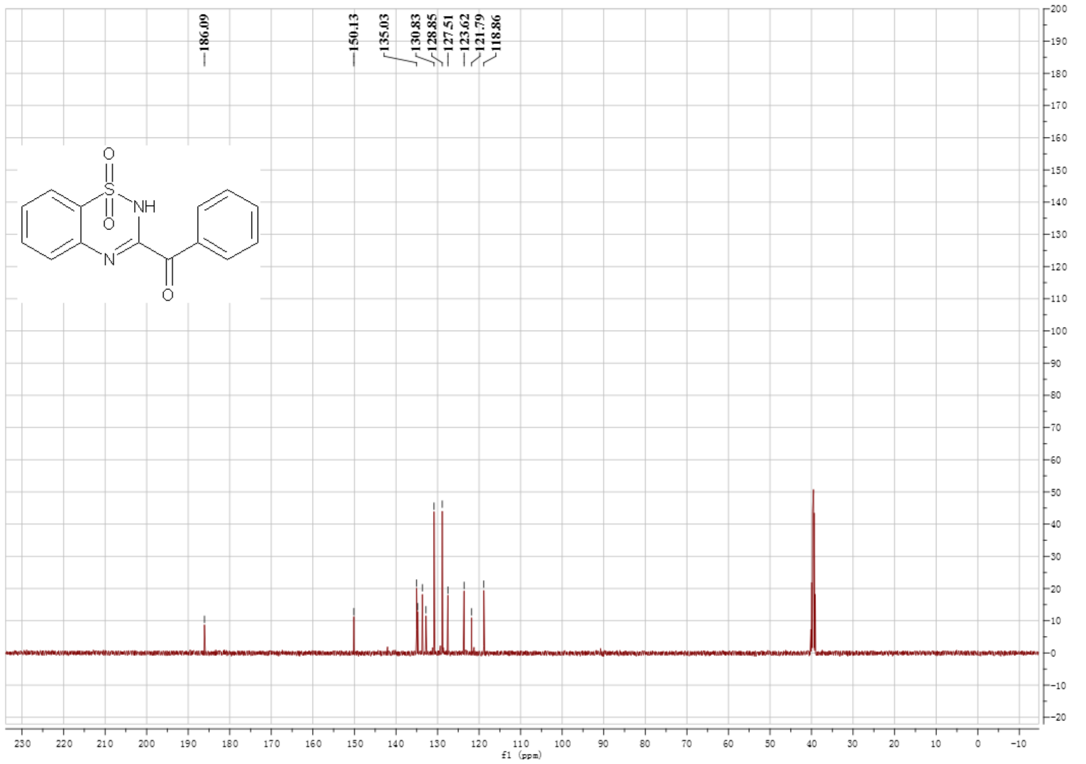

^{1}H NMR of compound 3b

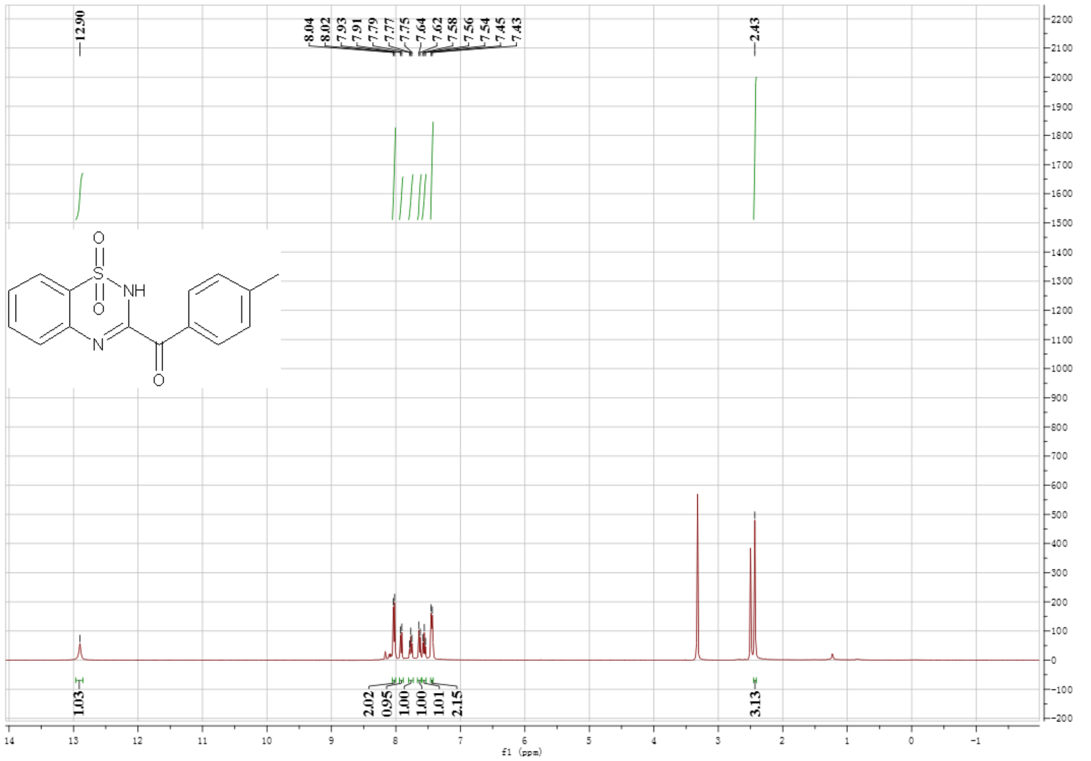

^{13}C NMR of compound 3b

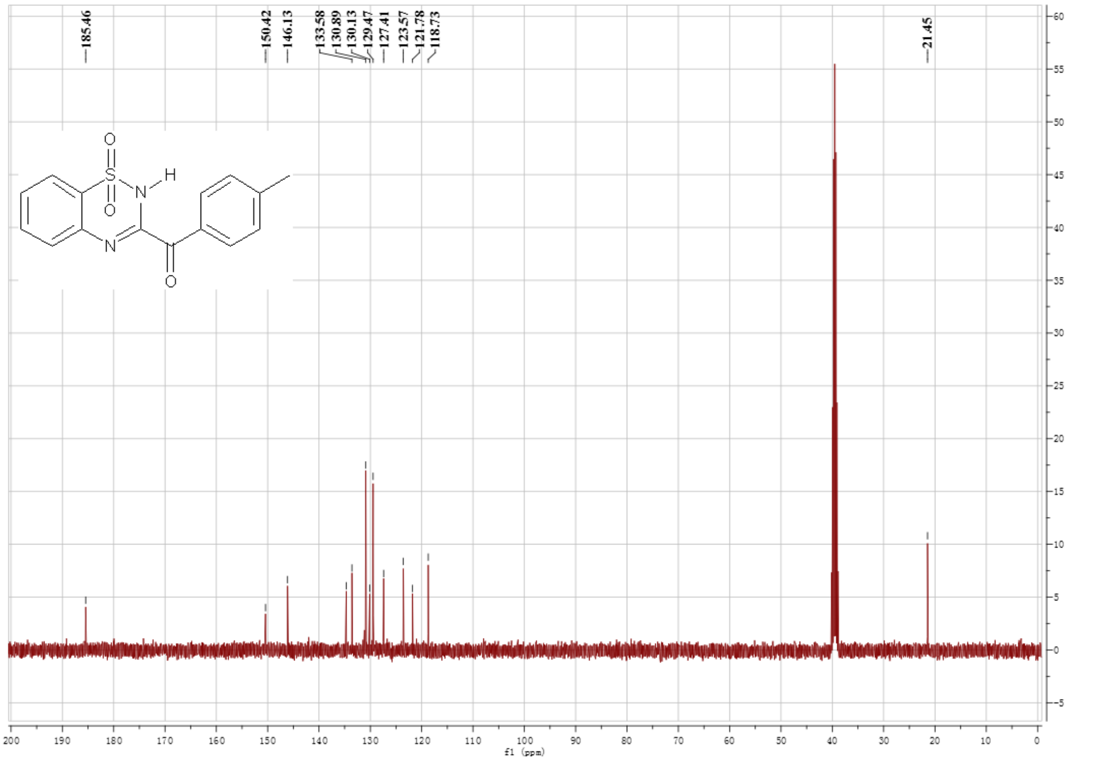

^{1}H NMR of compound 3c

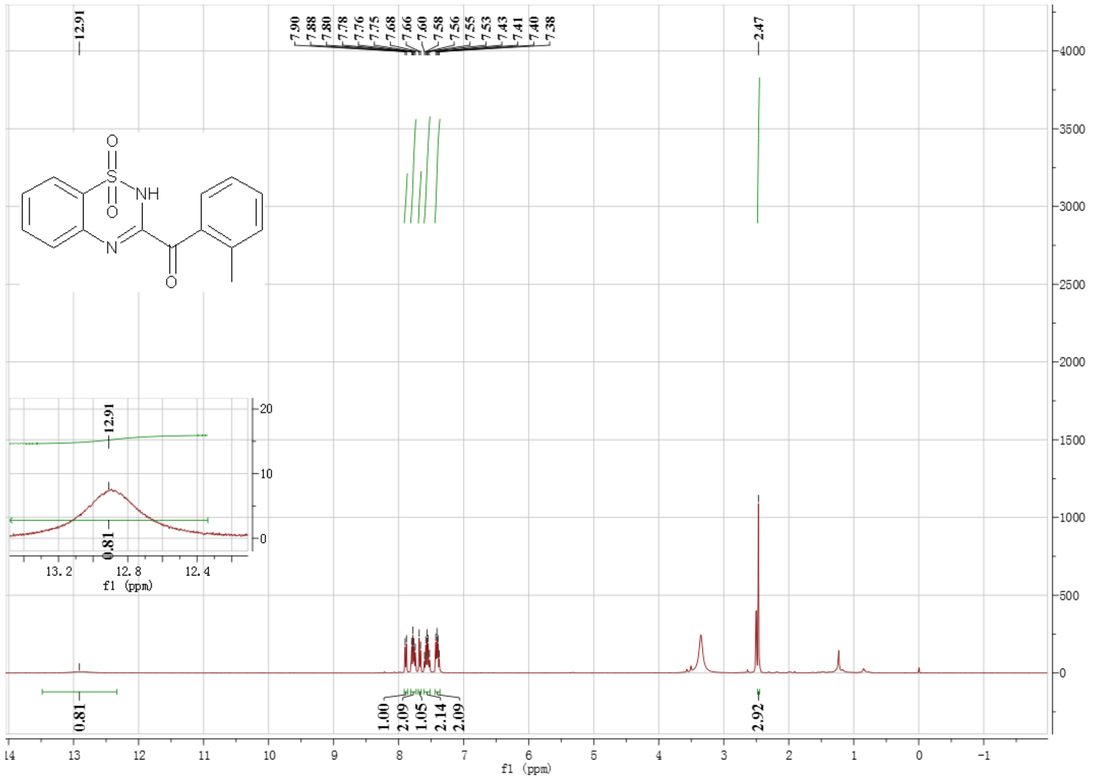

^{13}C NMR of compound 3c

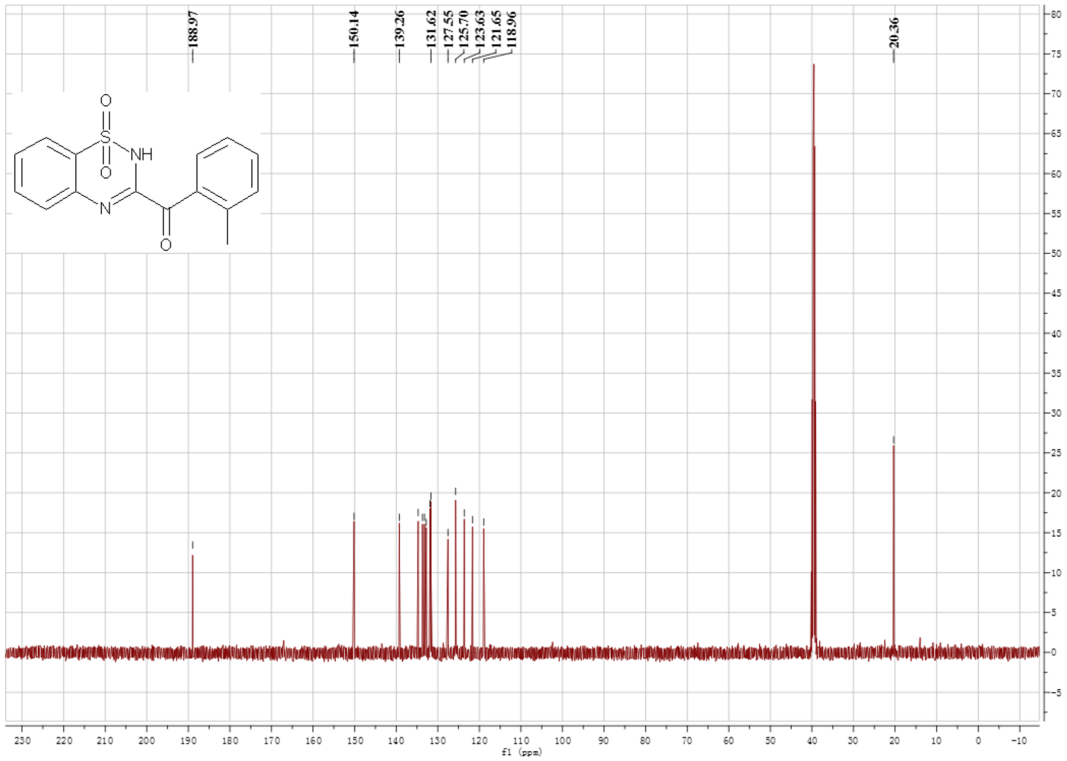

^{1}H NMR of compound 3d

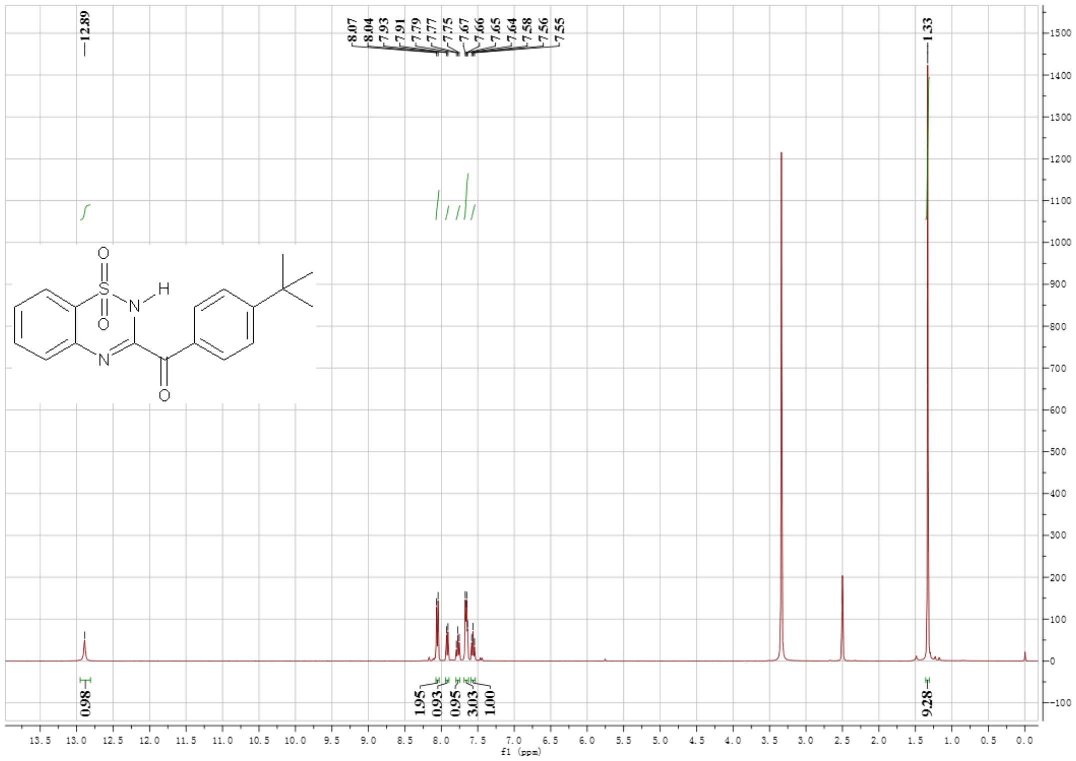

^{13}C NMR of compound 3d

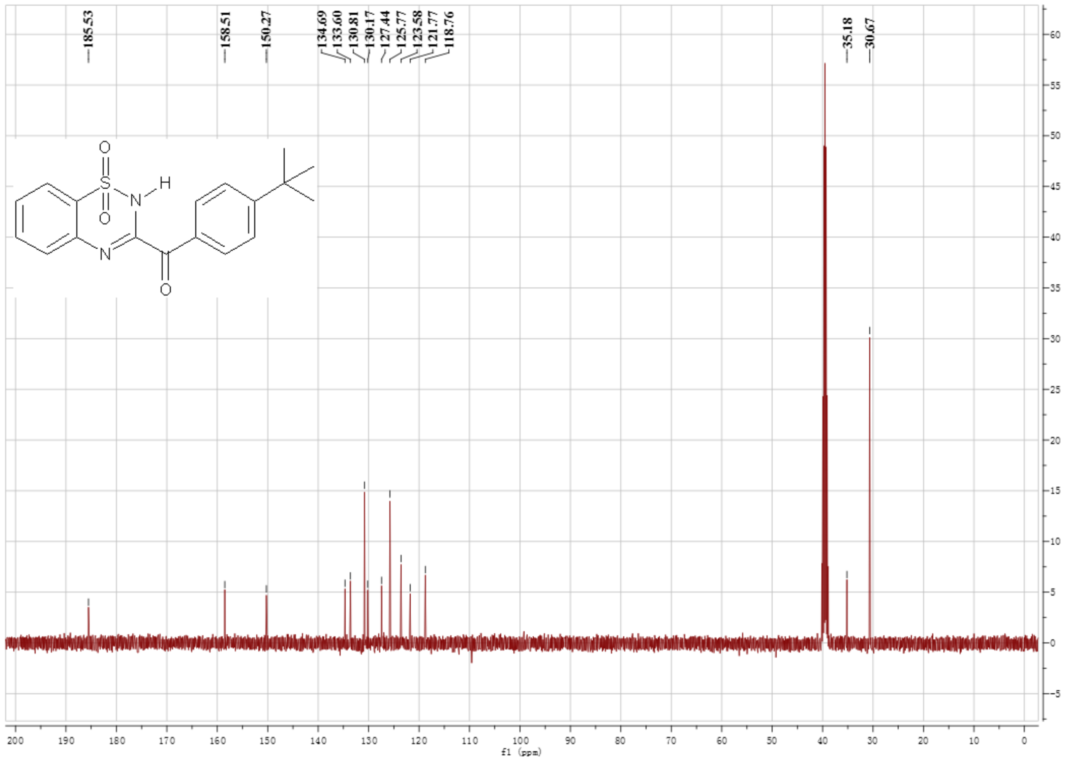

1H NMR of compound 3e

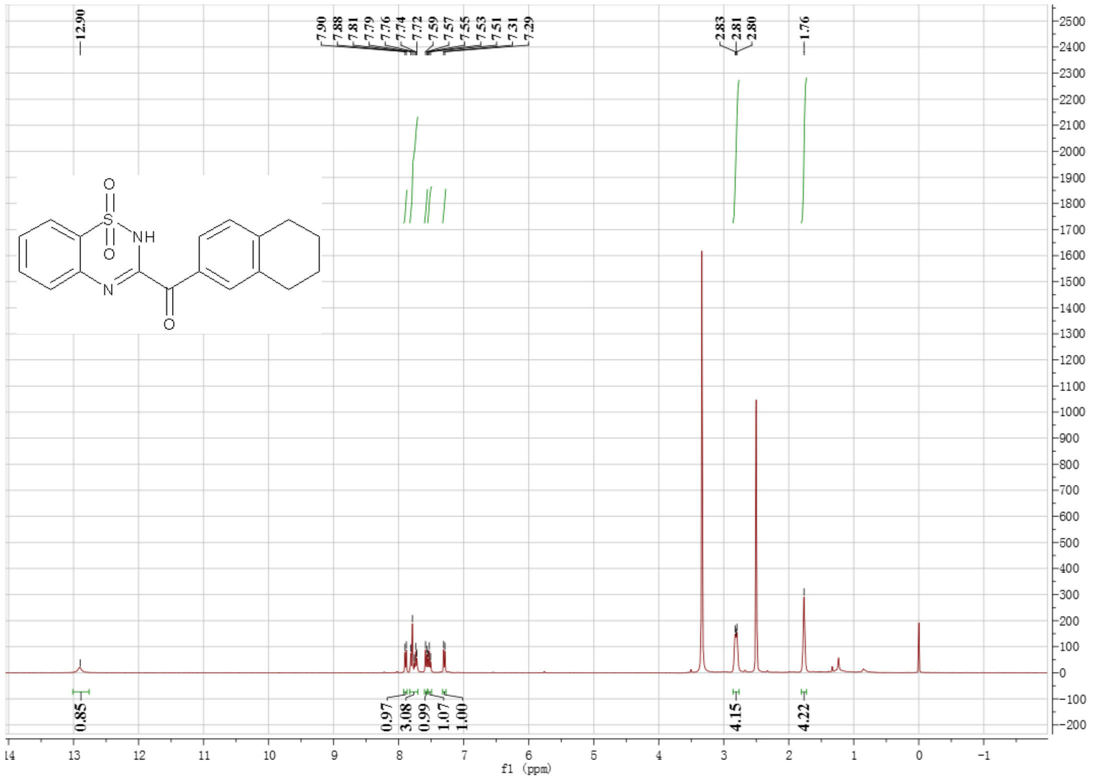

13C NMR of compound 3e

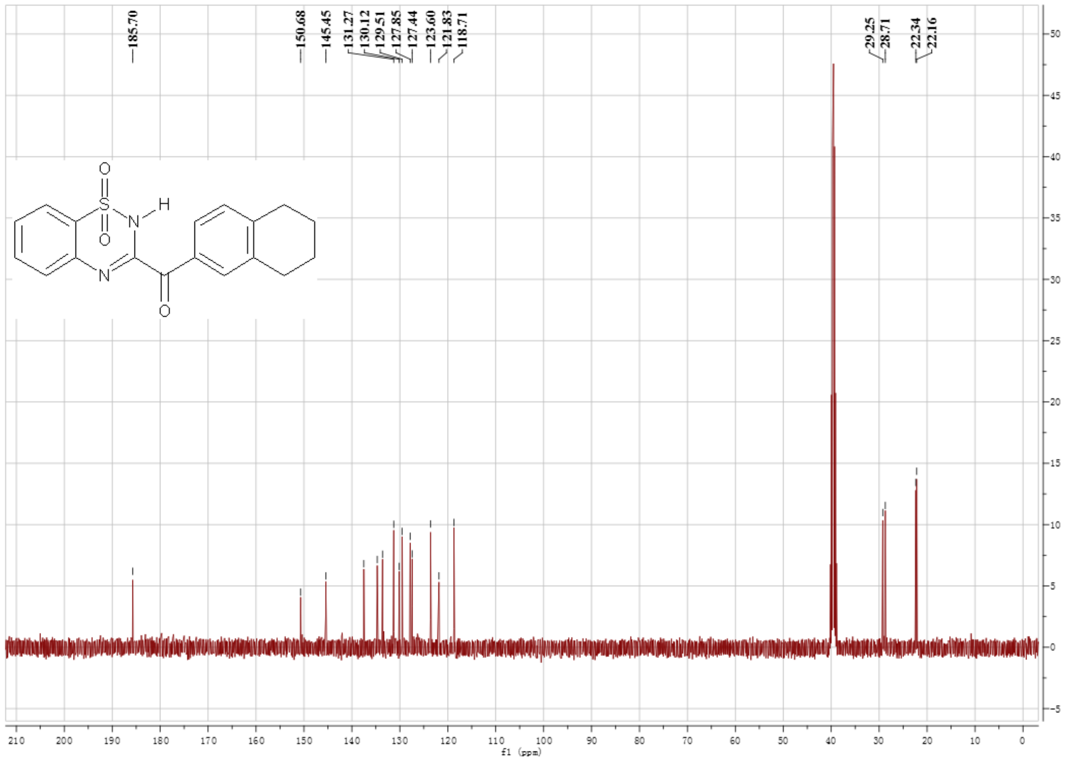

^{1}H NMR of compound 3f

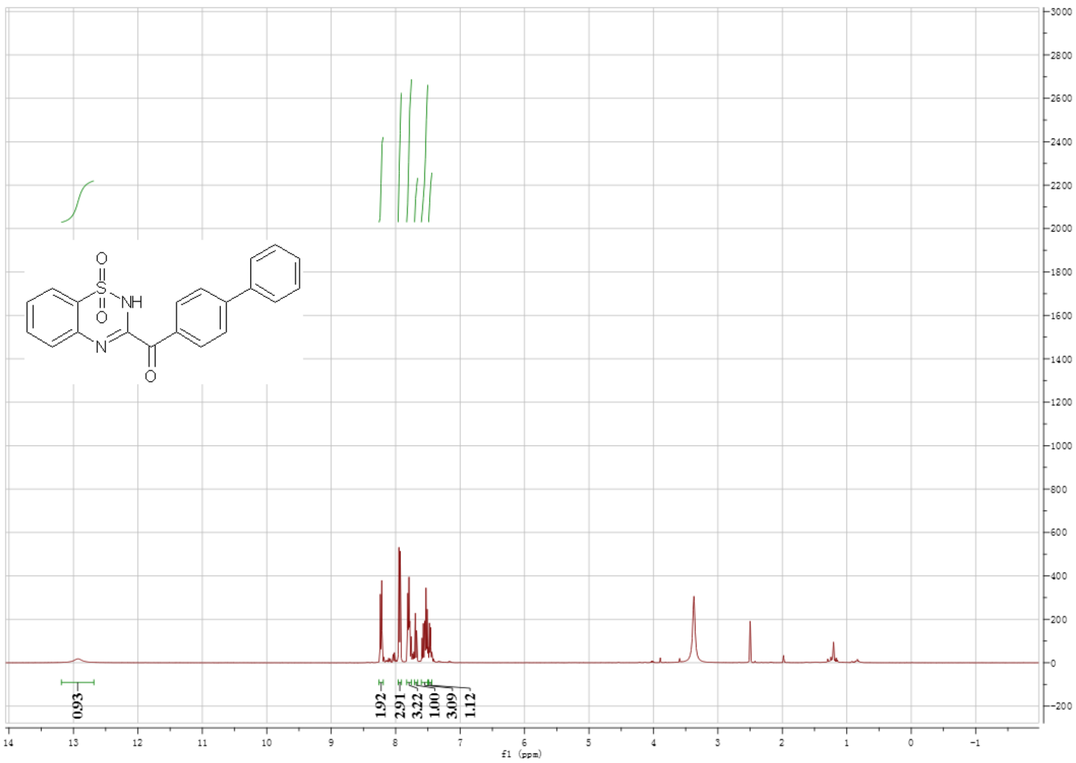

^{13}C NMR of compound 3f

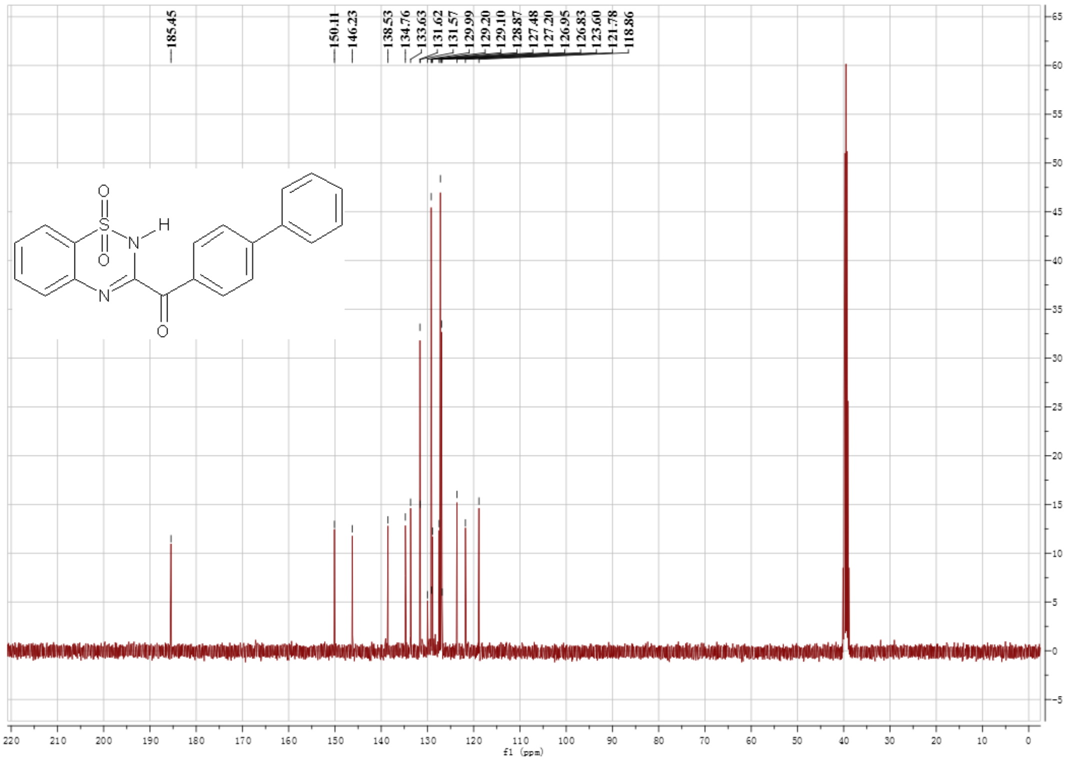

^{1}H NMR of compound 3g

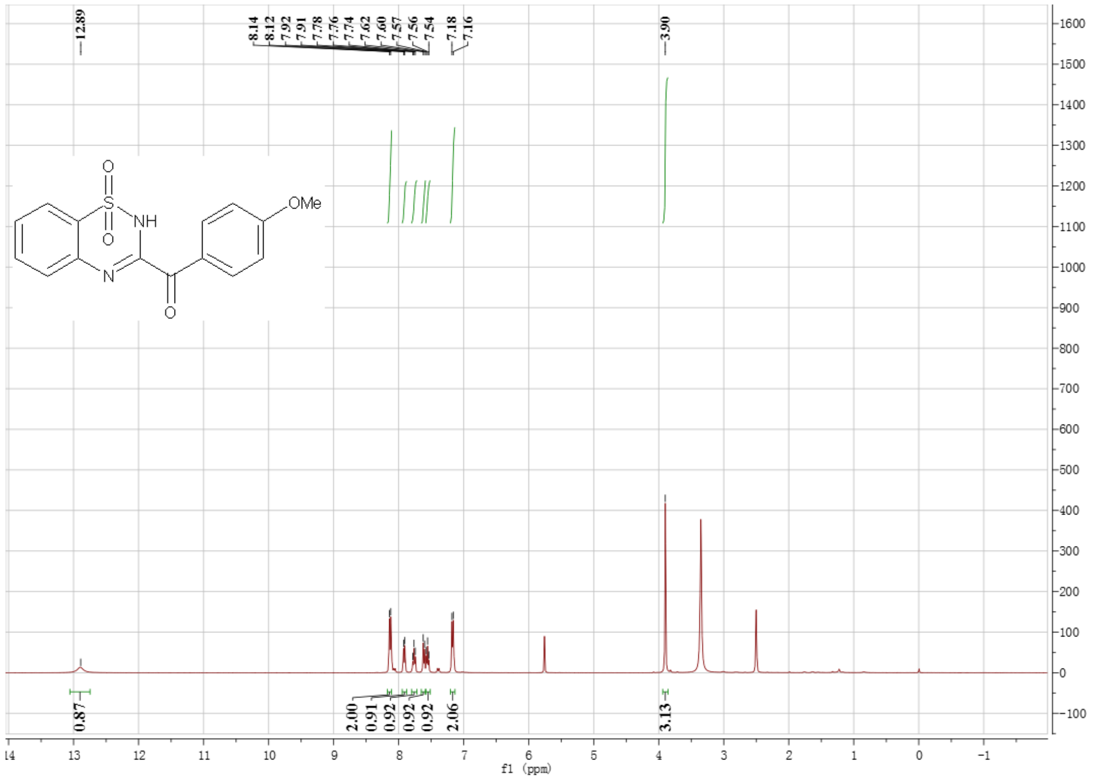

^{13}C NMR of compound 3g

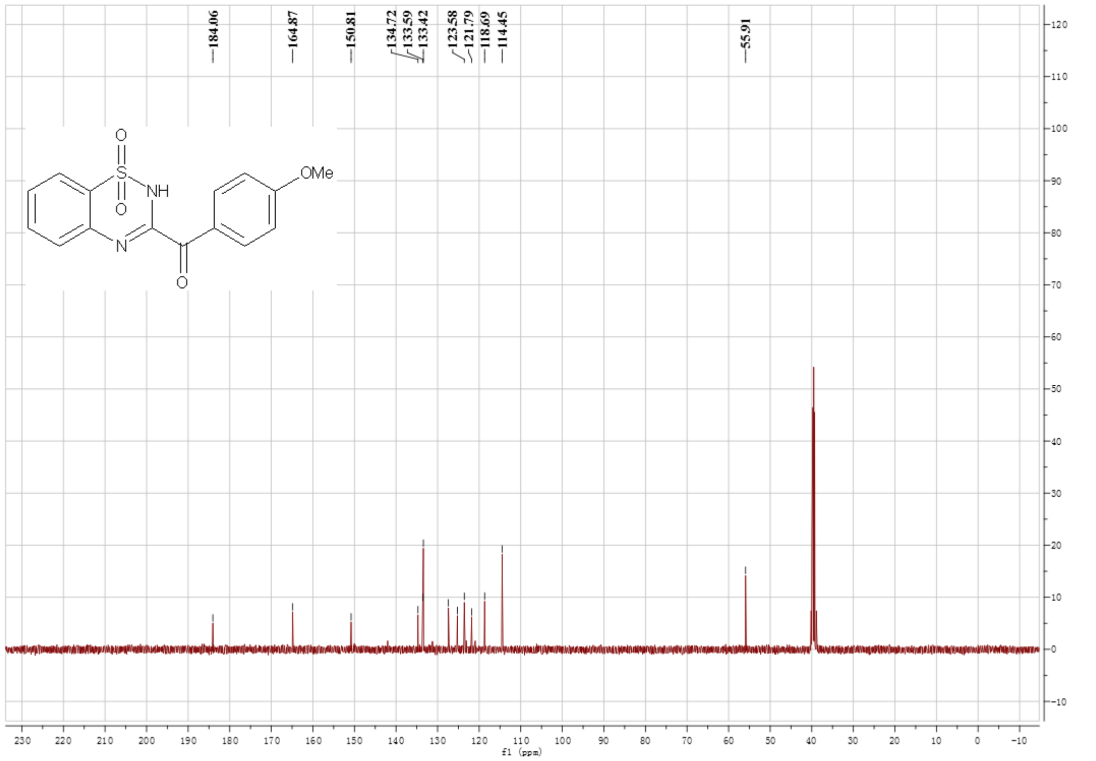

^{1}H NMR of compound 3h

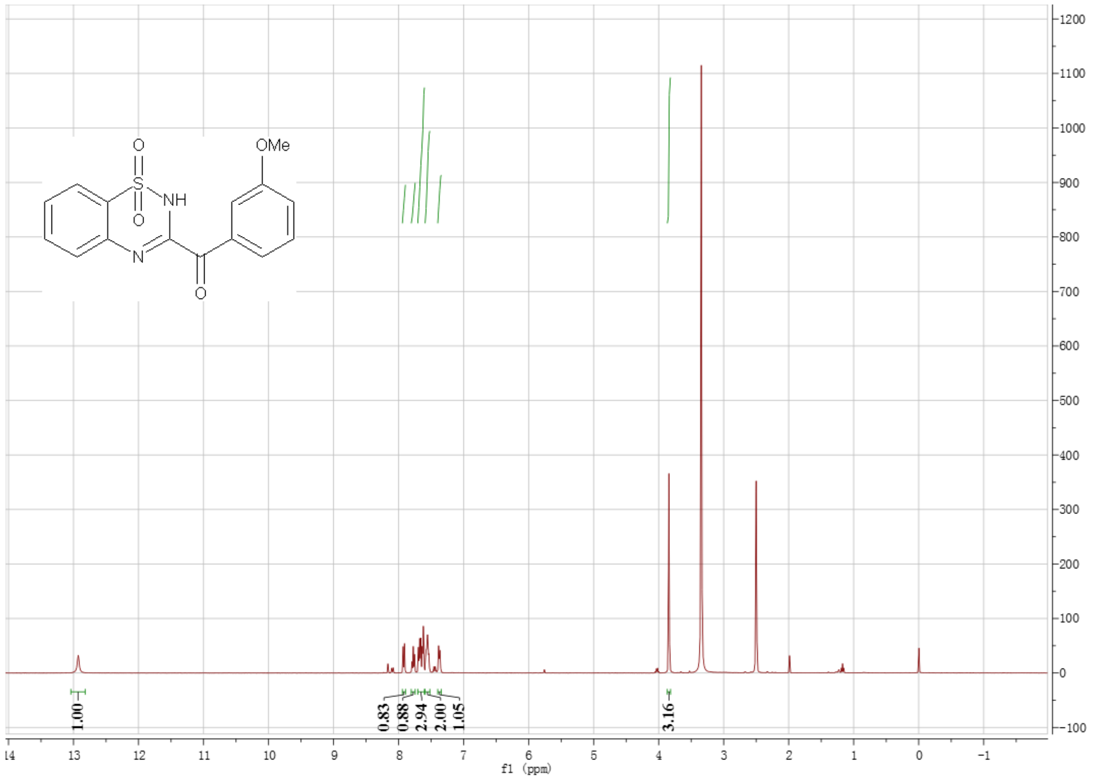

^{13}C NMR of compound 3h

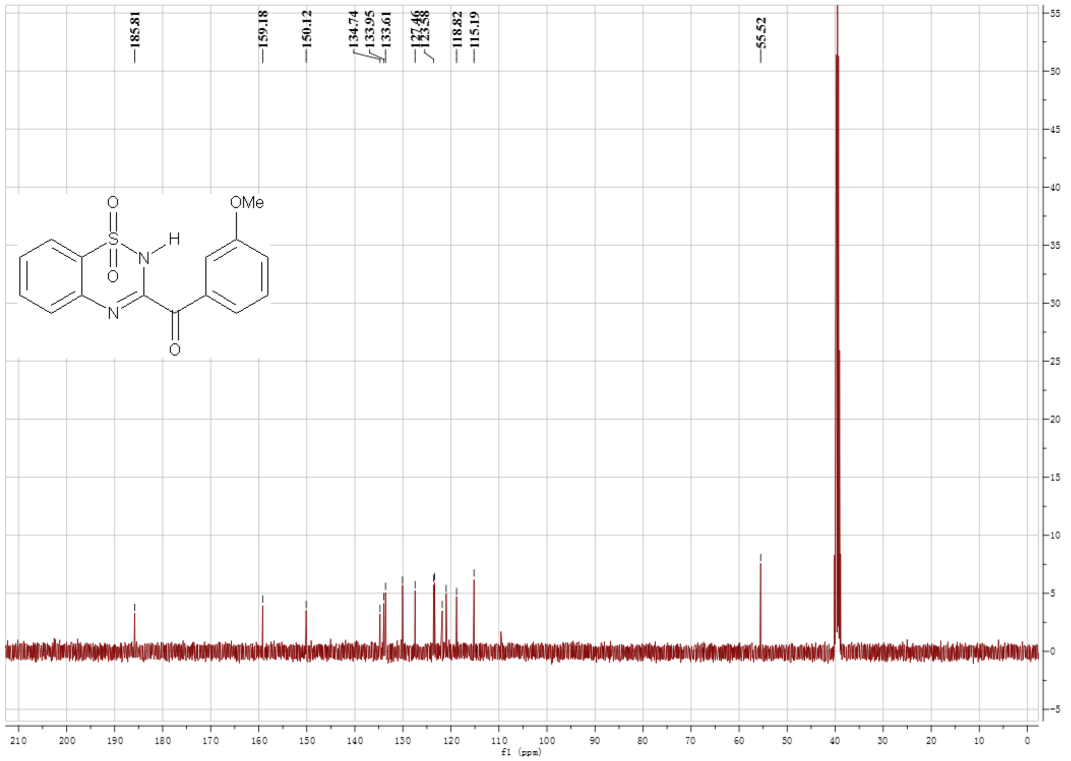

^{1}H NMR of compound 3i

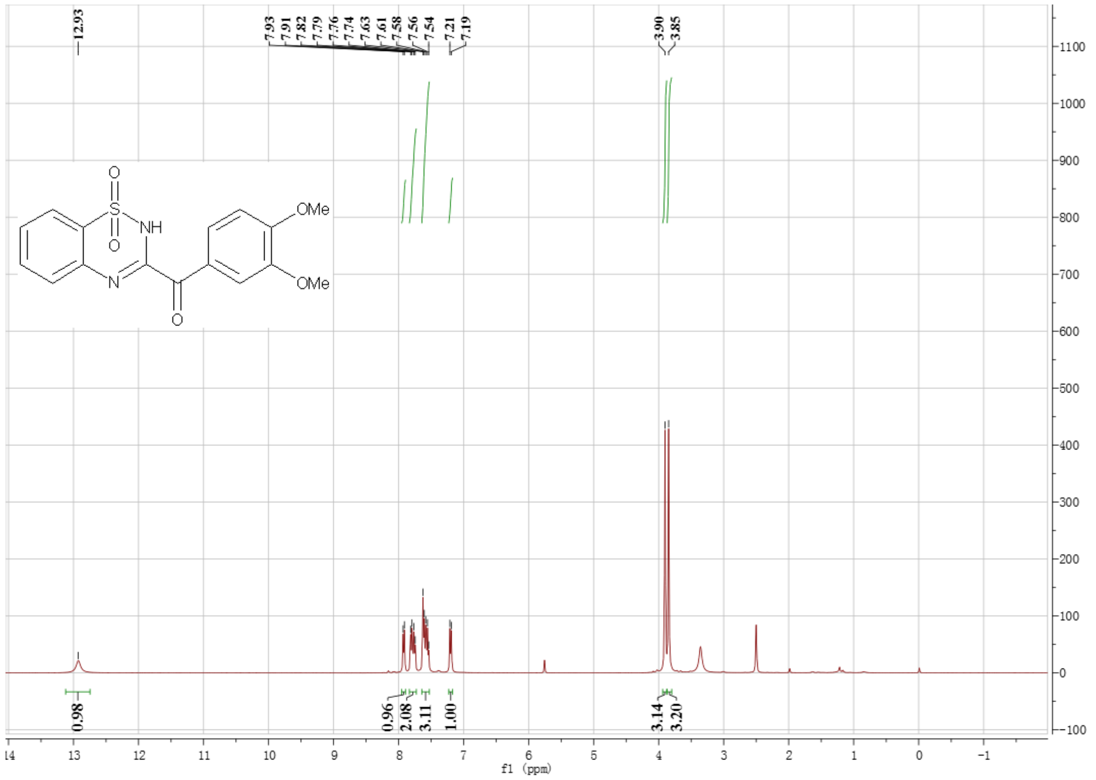

^{13}C NMR of compound 3i

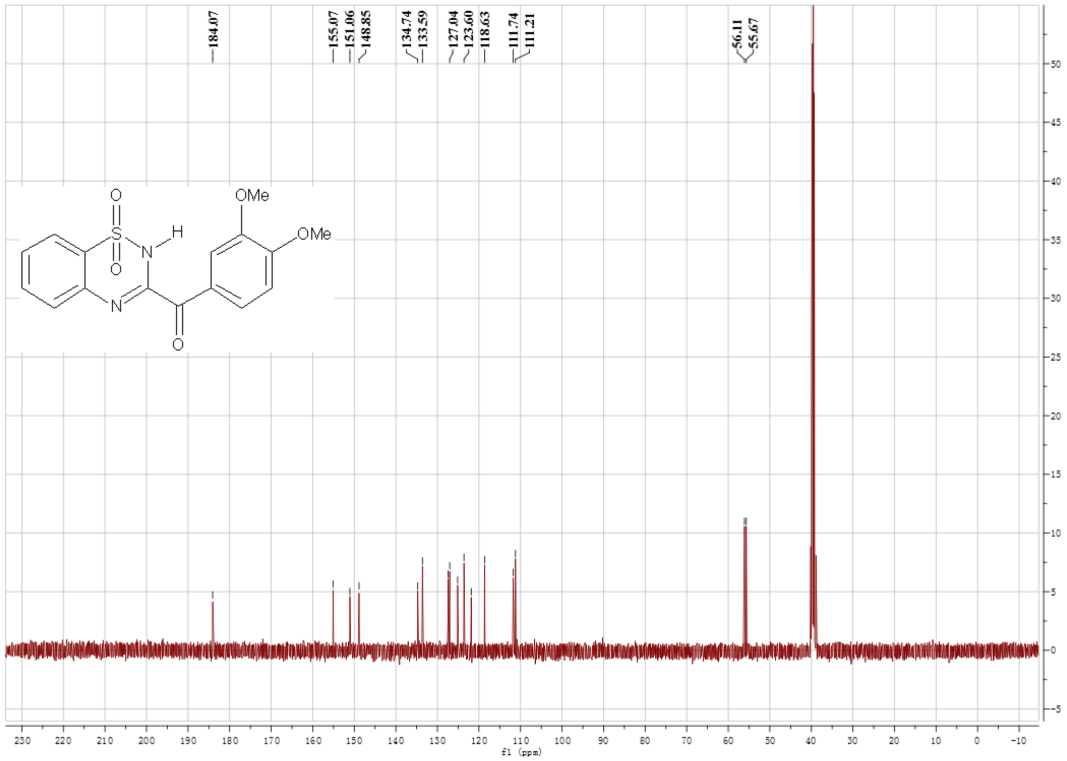

^{1}H NMR of compound 3j

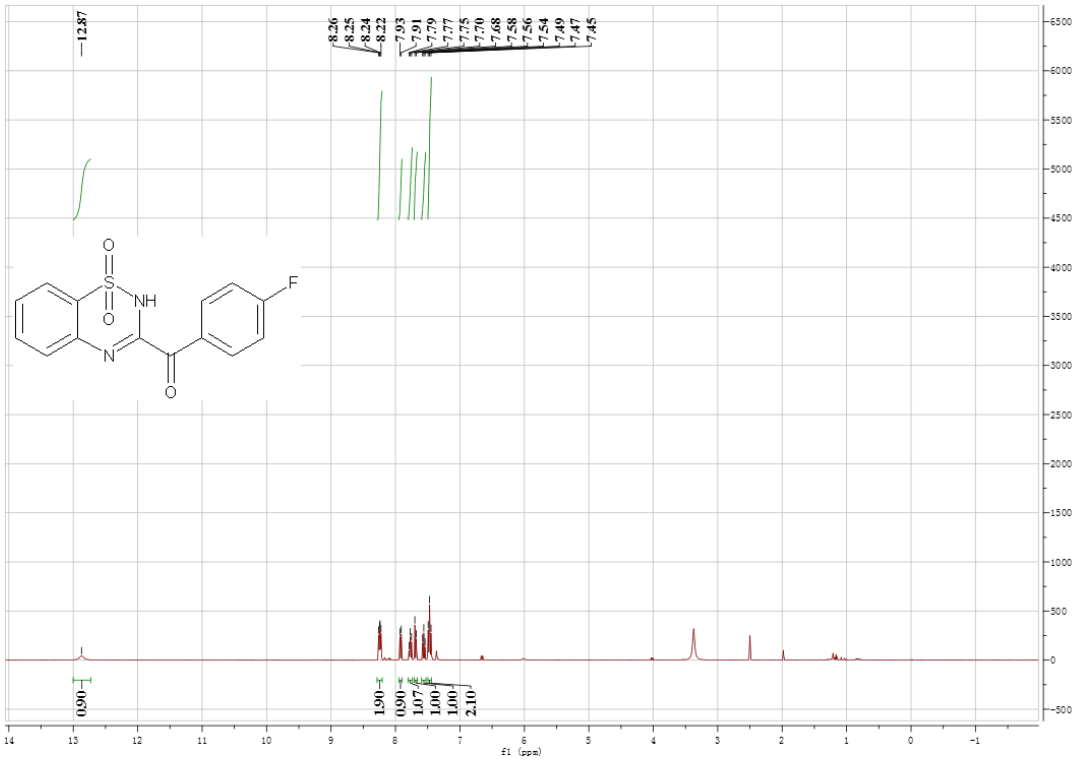

^{13}C NMR of compound 3j

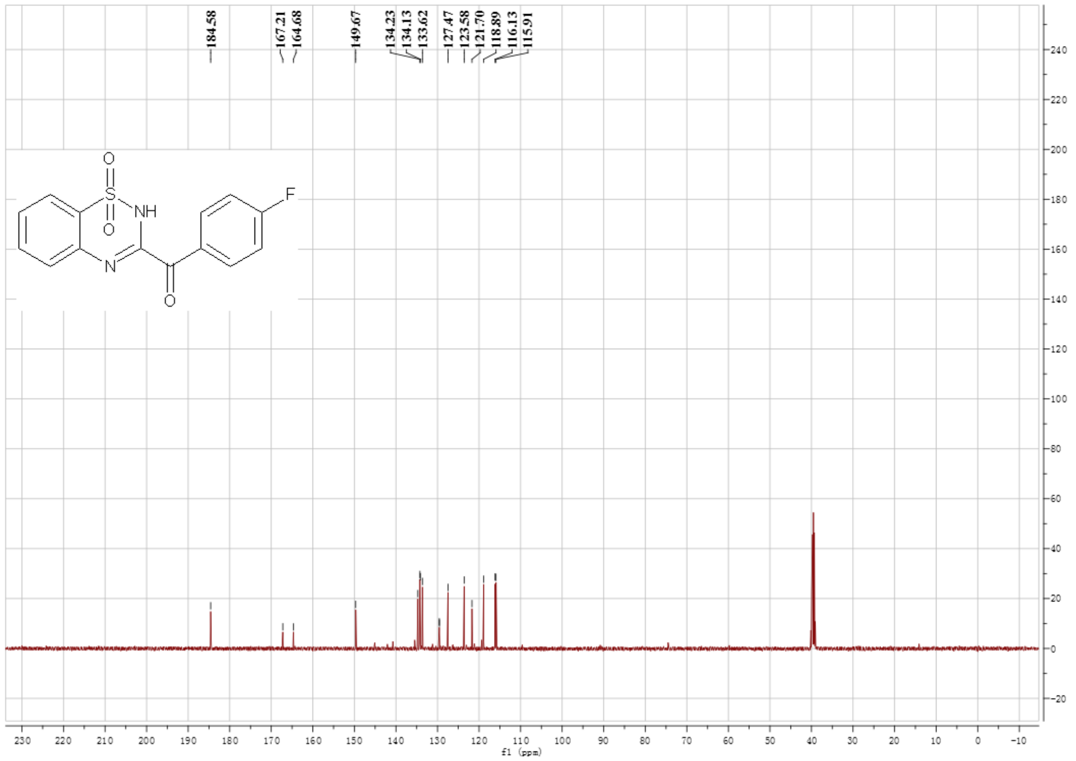

^{1}H NMR of compound 3k

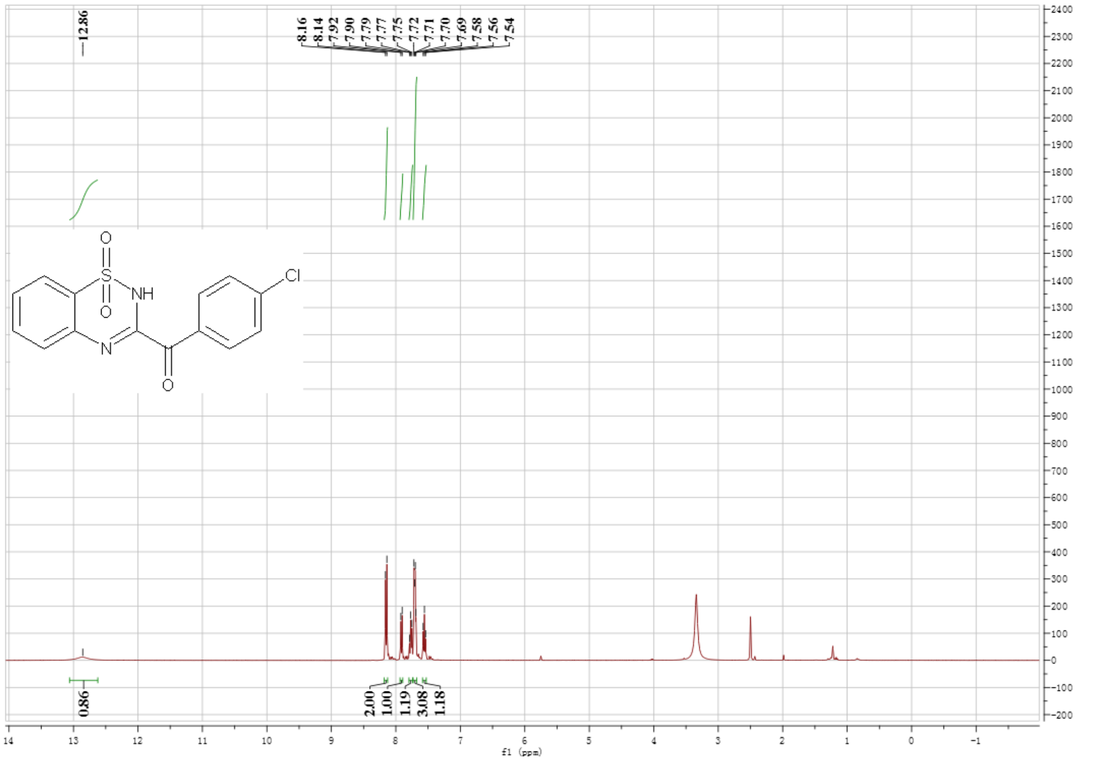

^{13}C NMR of compound 3k

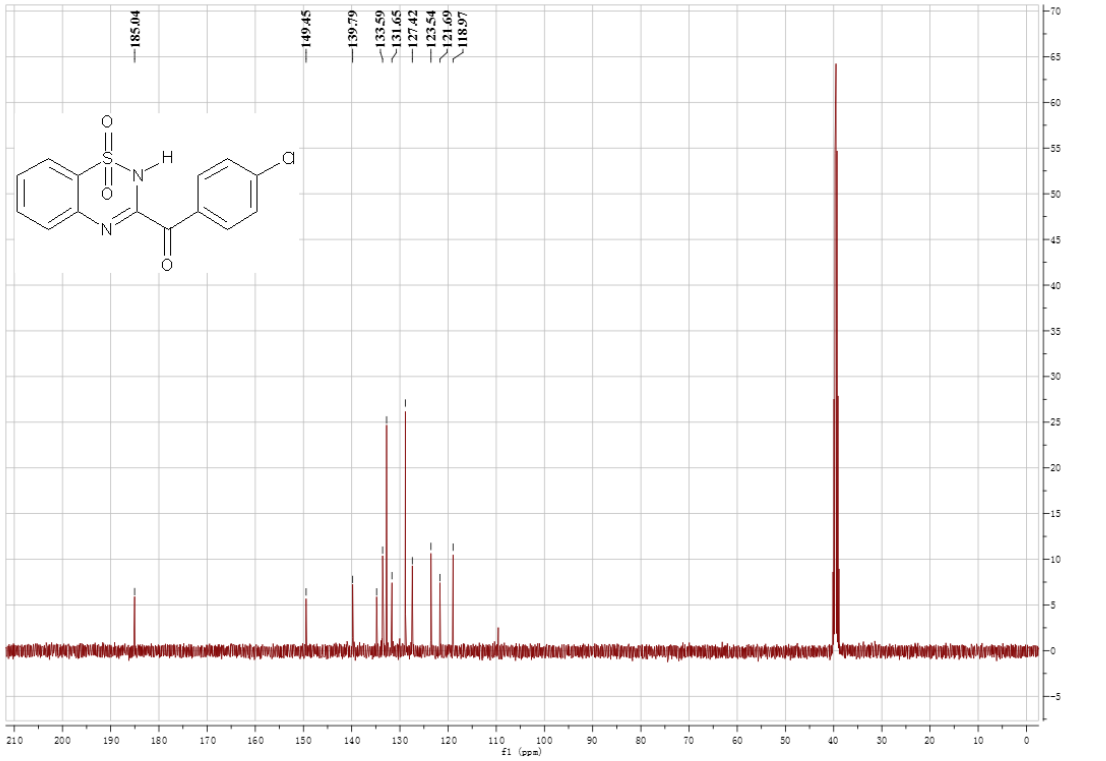

^{1}H NMR of compound 3l

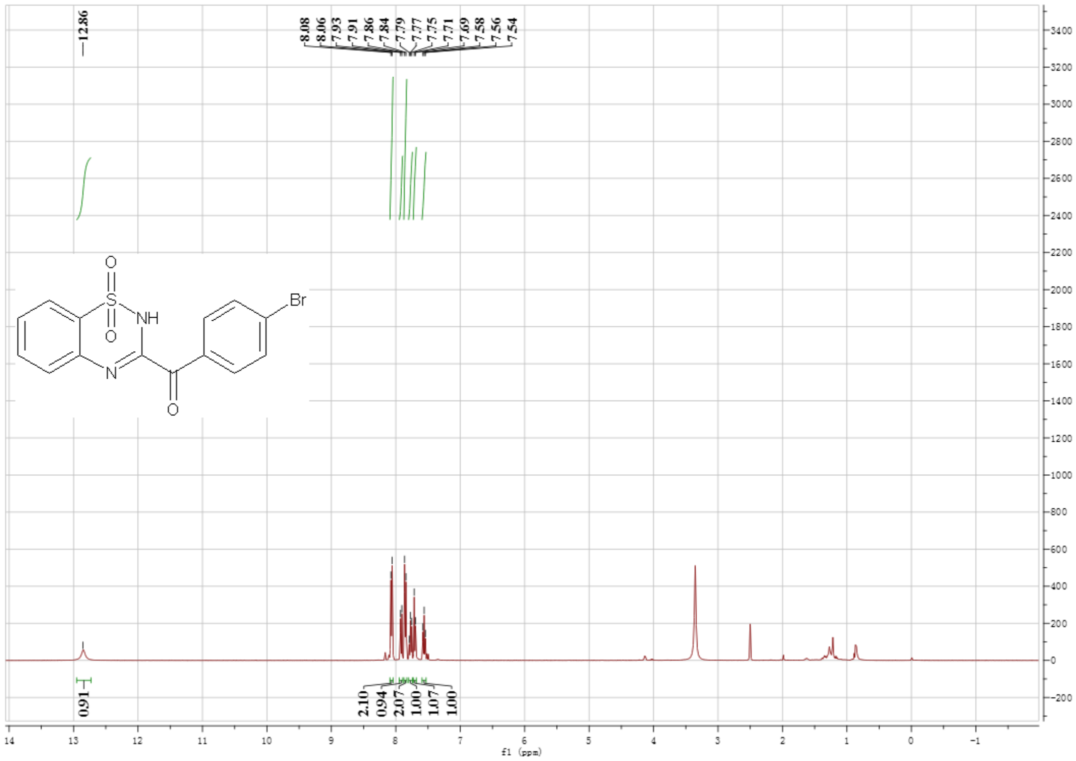

^{13}C NMR of compound 3l

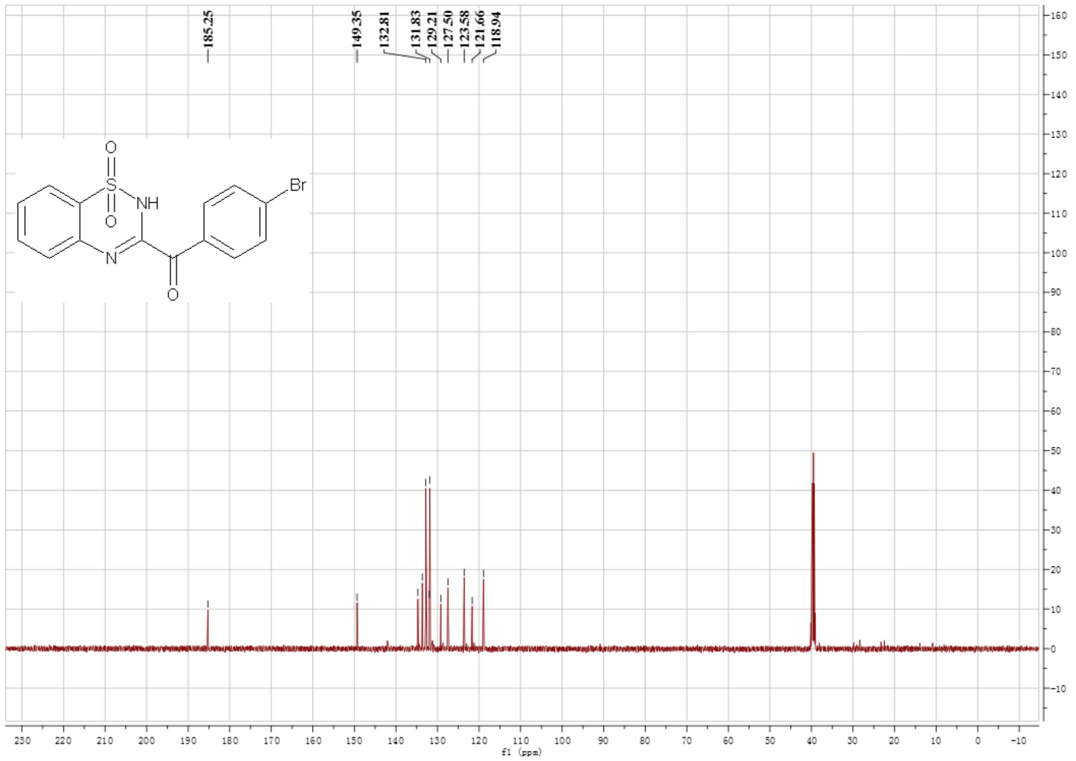

^{1}H NMR of compound 3m

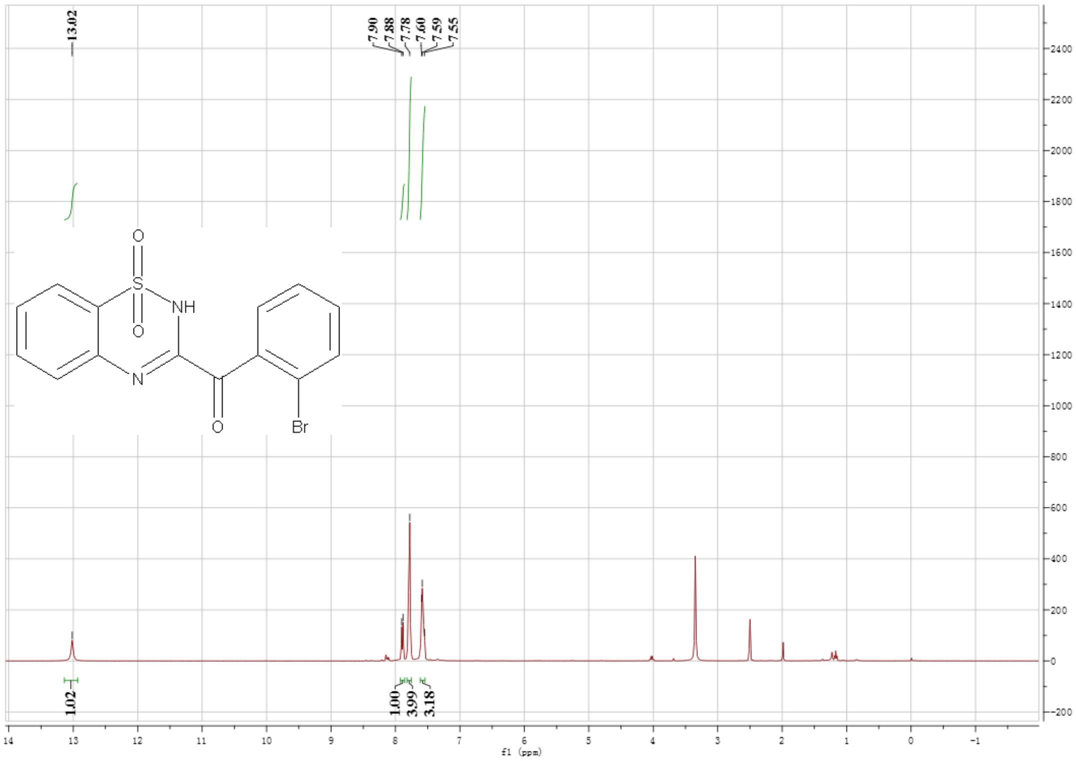

^{13}C NMR of compound 3m

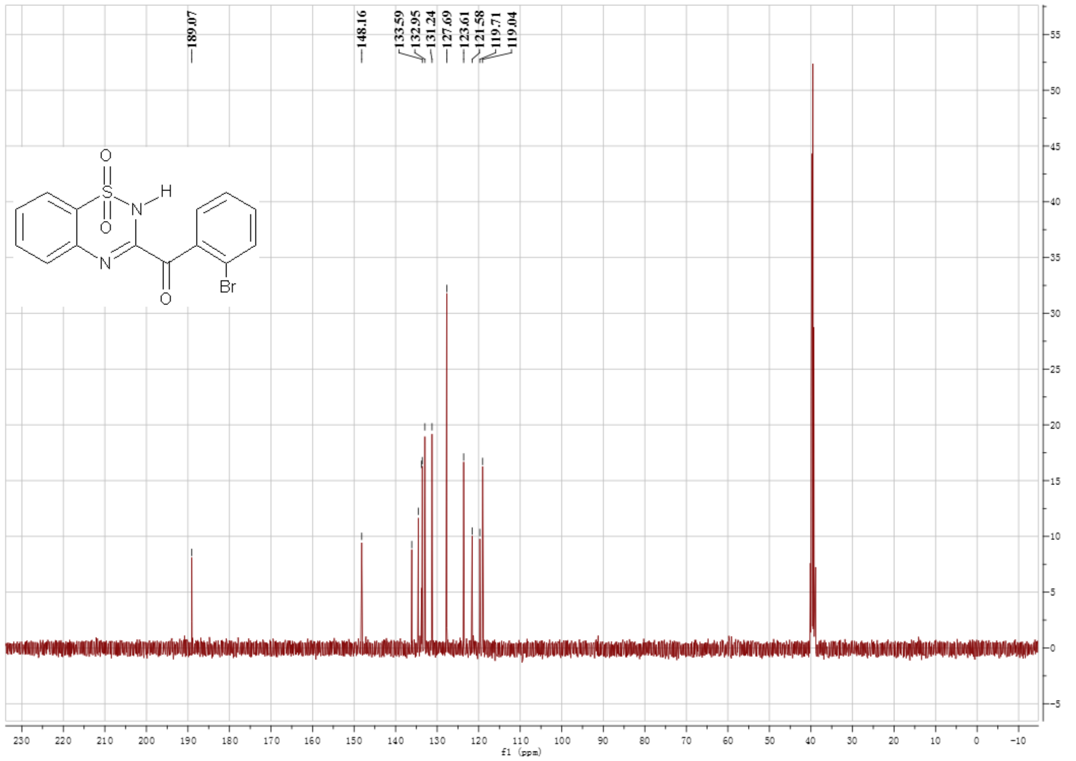

^{1}H NMR of compound 3n

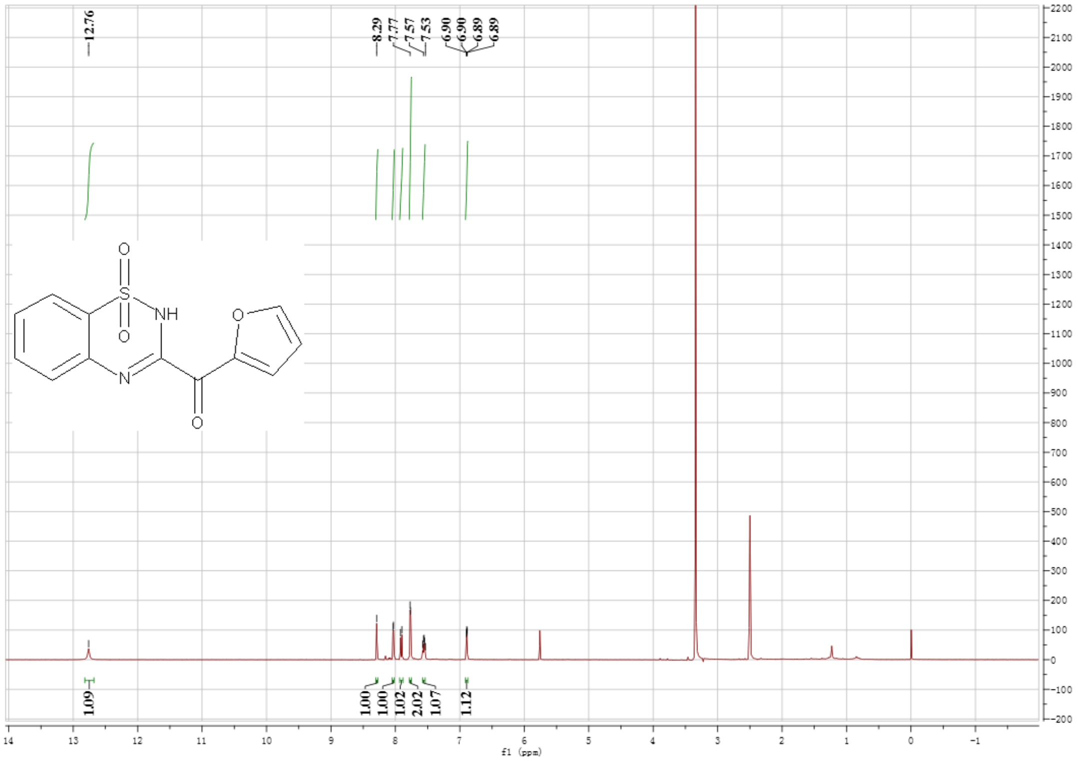

^{13}C NMR of compound 3n

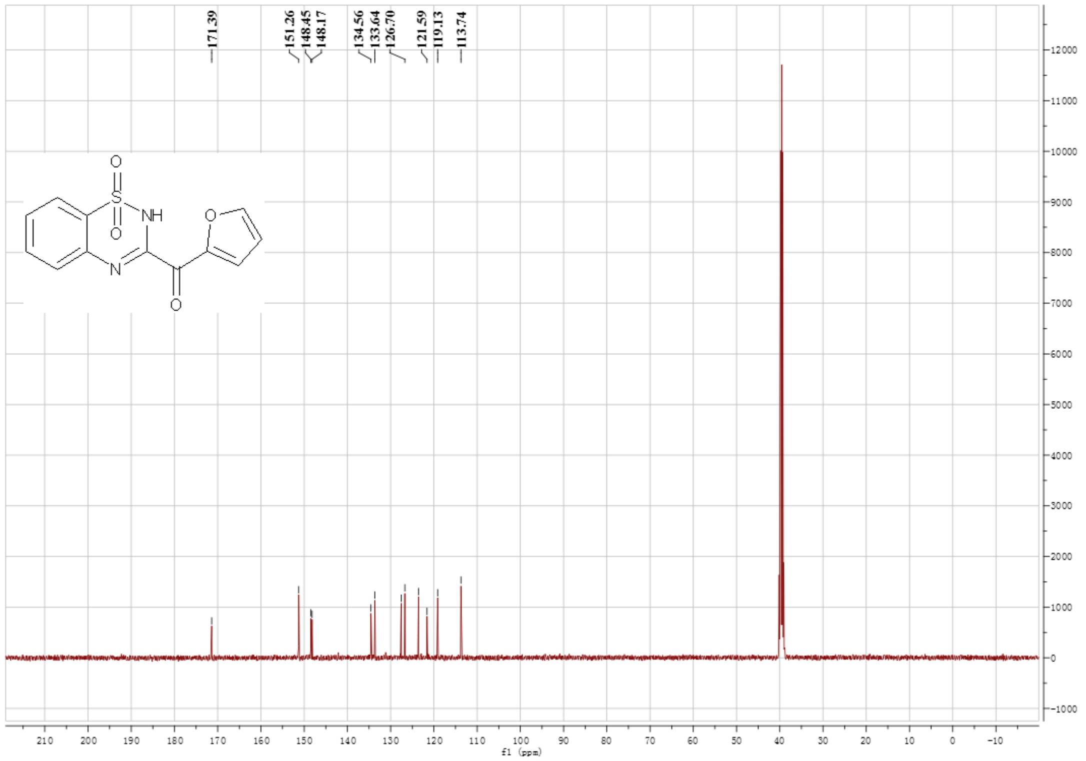

^{1}H NMR of compound 3o

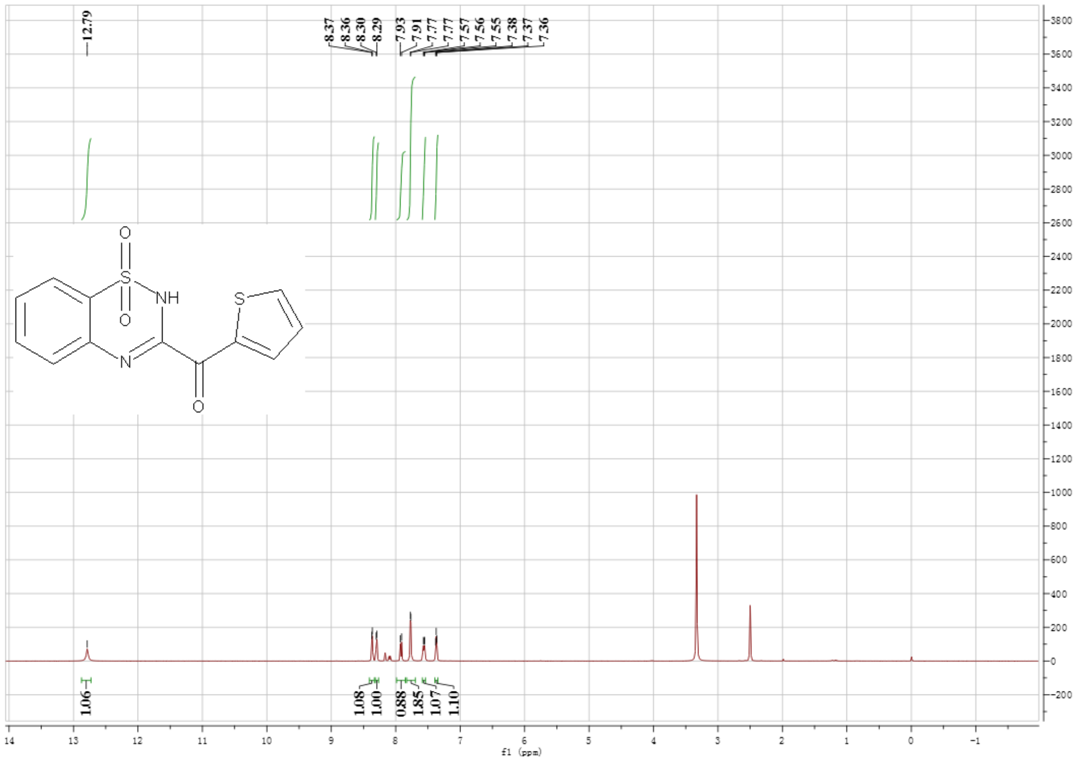

^{13}C NMR of compound 3o

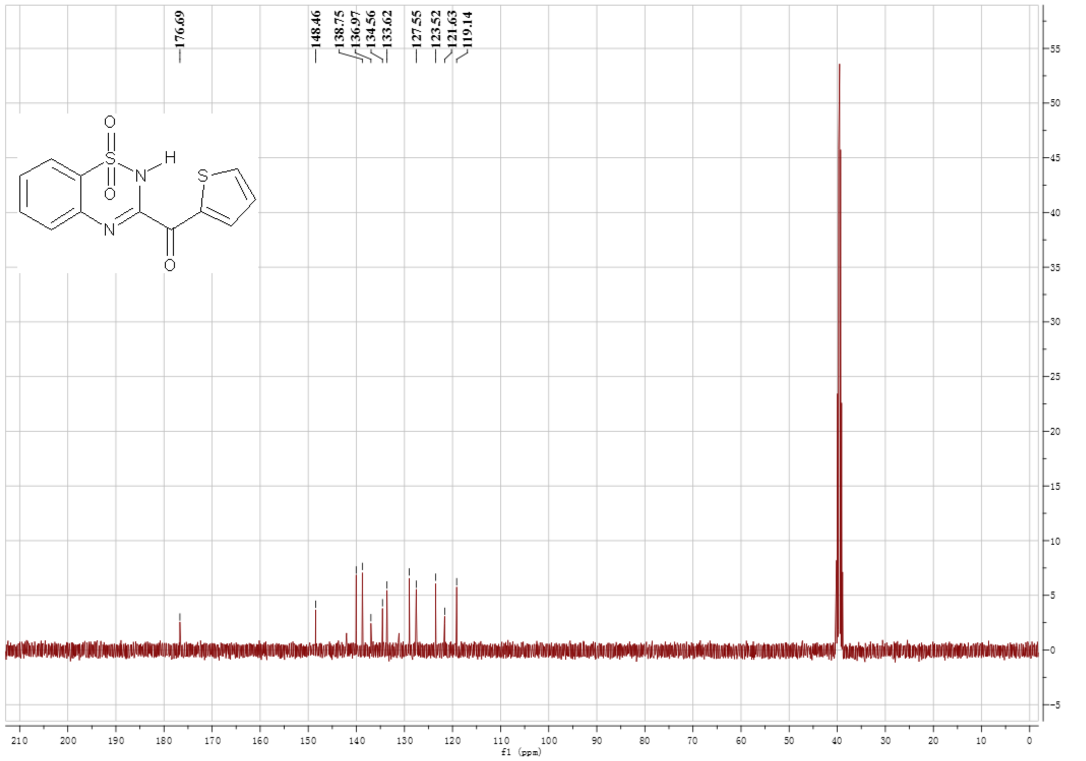

^{1}H NMR of compound 3p

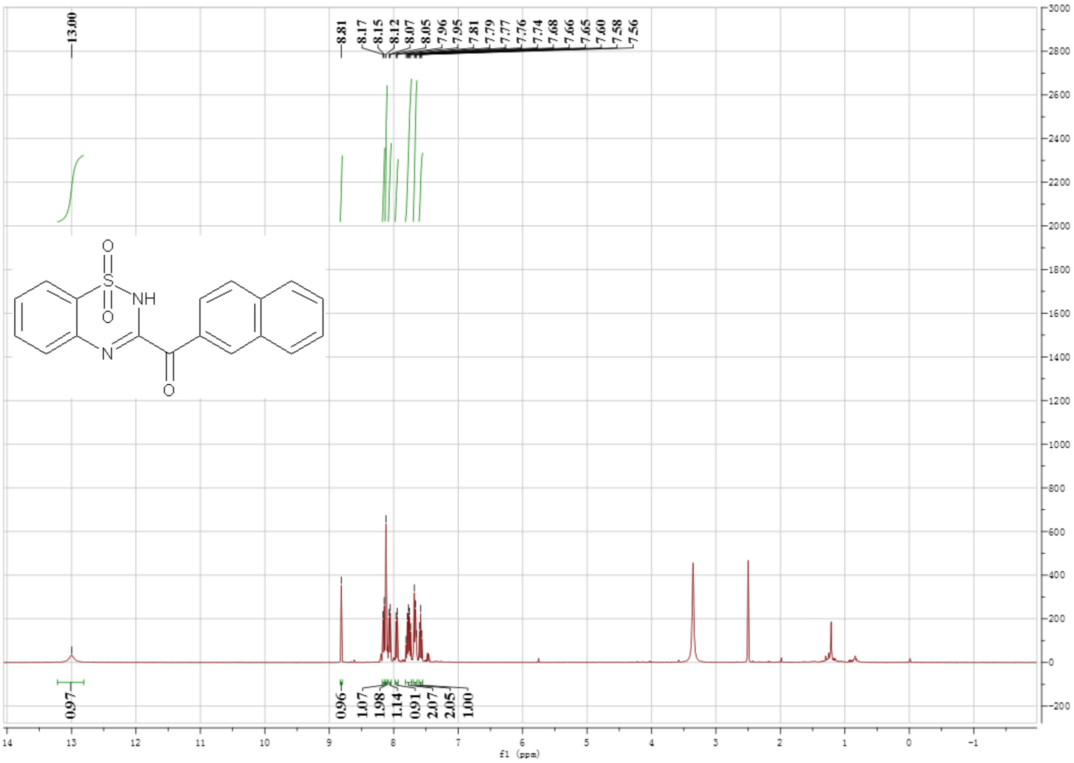

^{13}C NMR of compound 3p

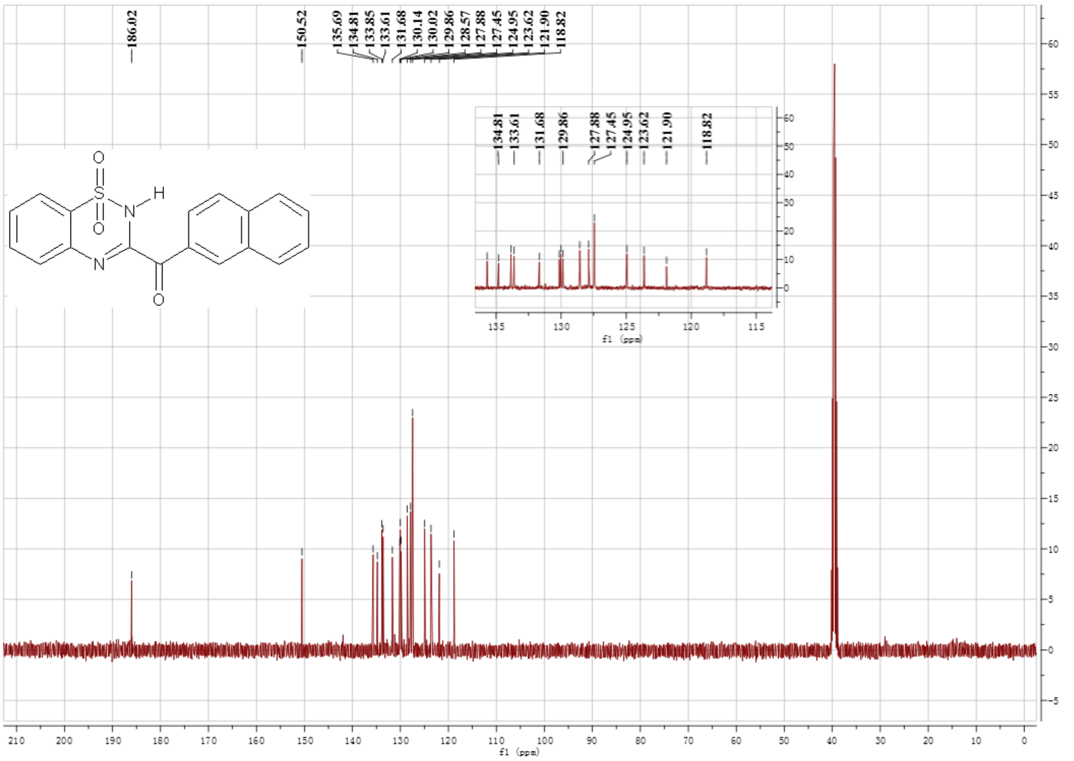

^{1}H NMR of compound 3q

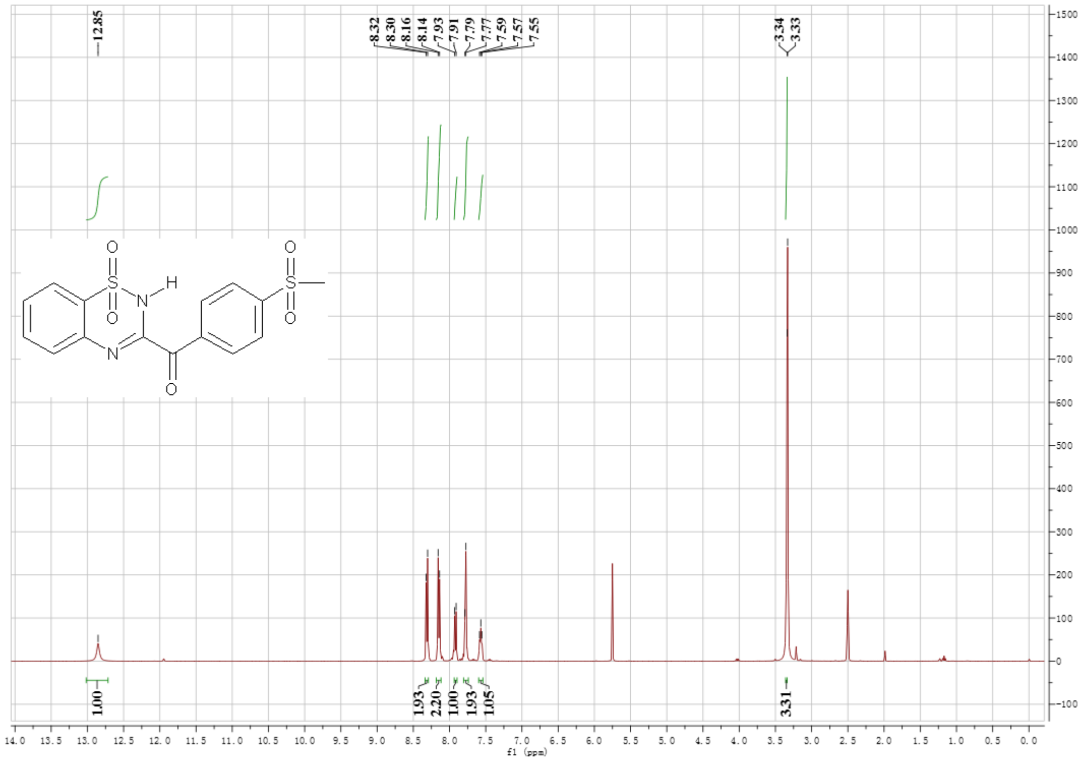

^{13}C NMR of compound 3q

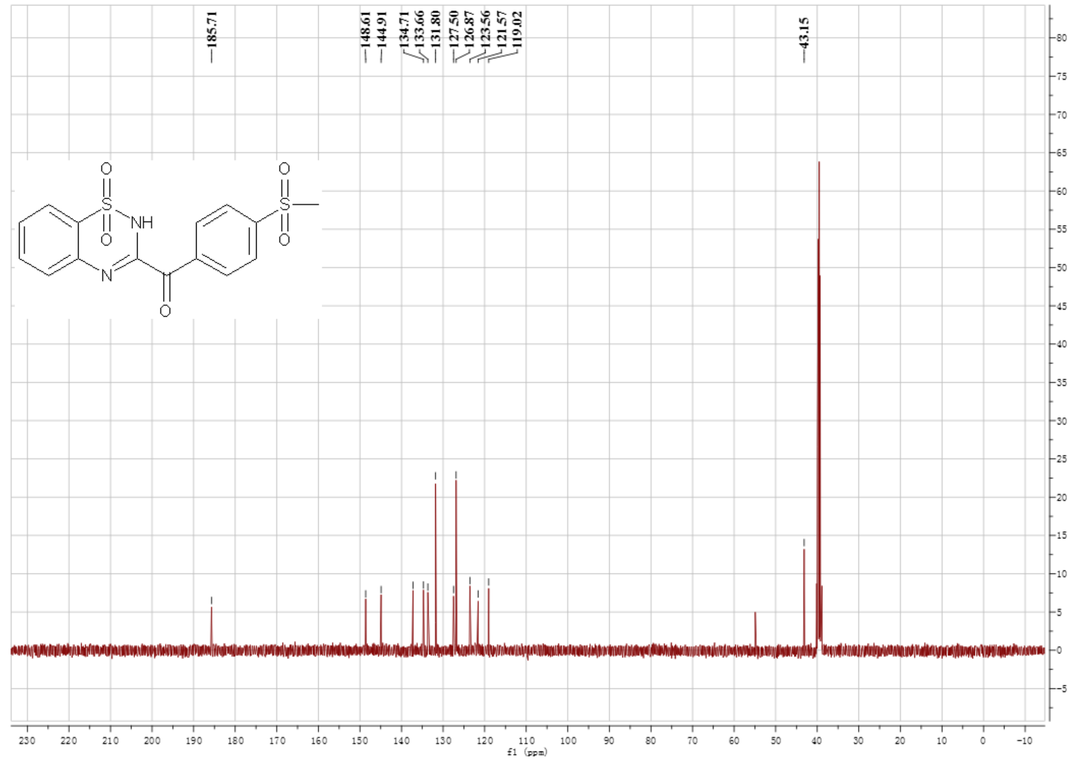

^{1}H NMR of compound 4a

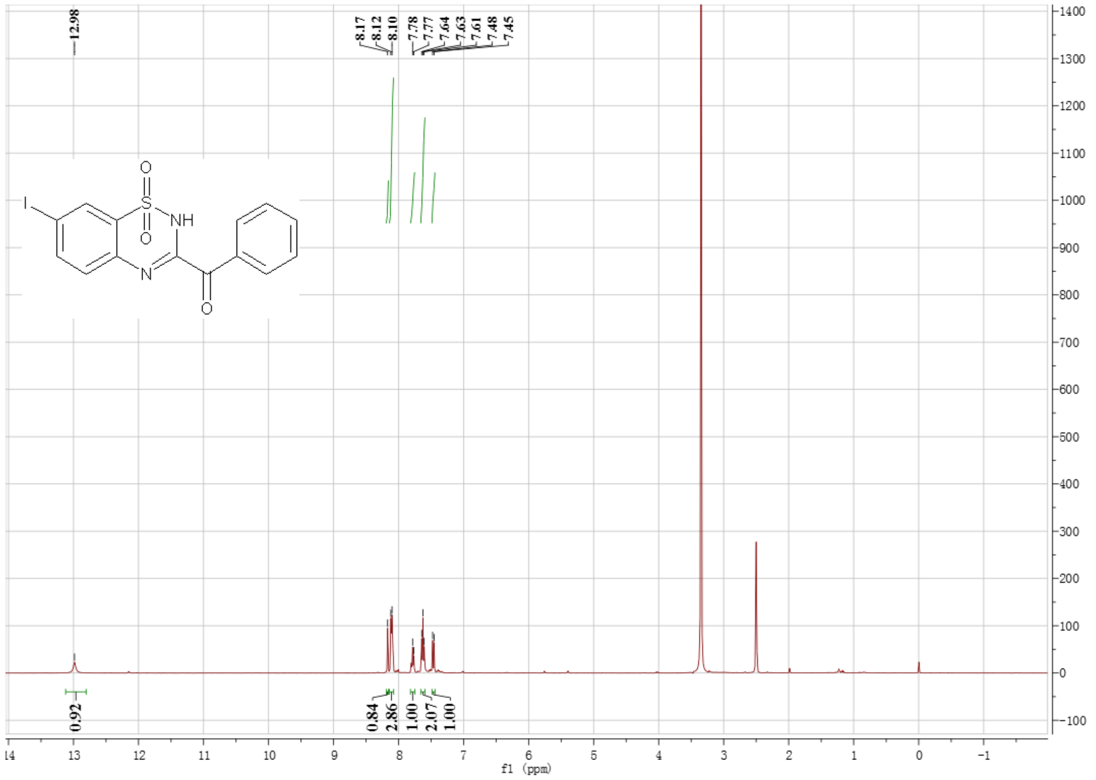

^{13}C NMR of compound 4a

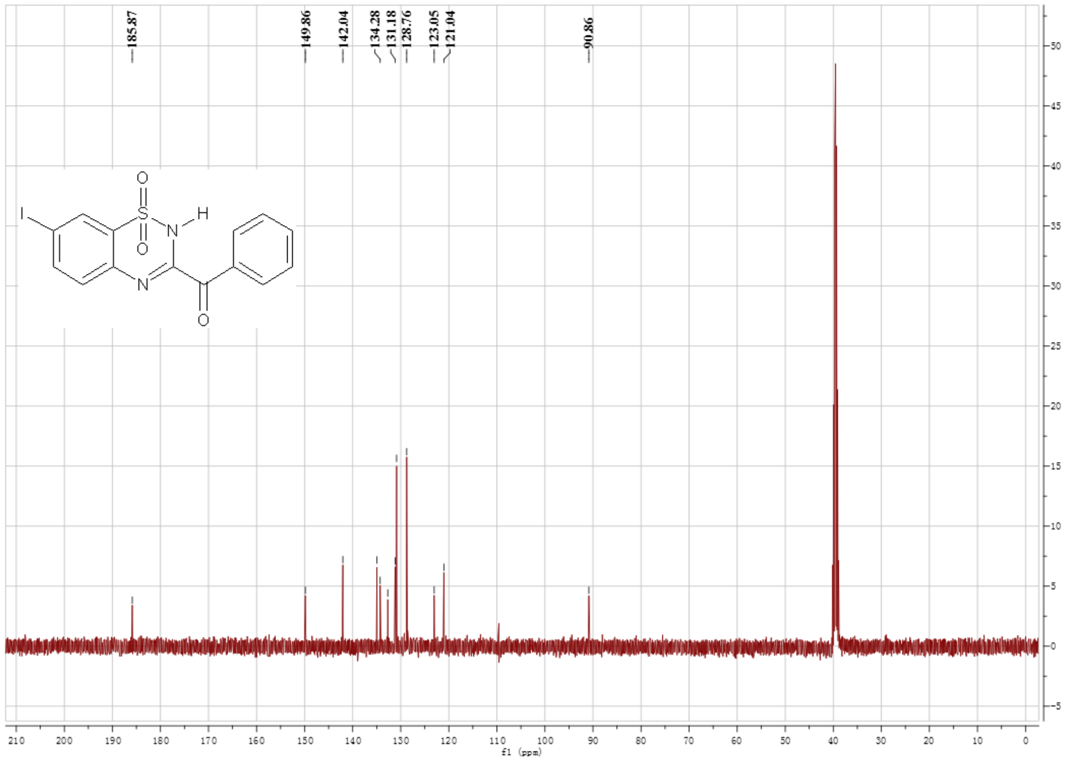

^{1}H NMR of compound 4b

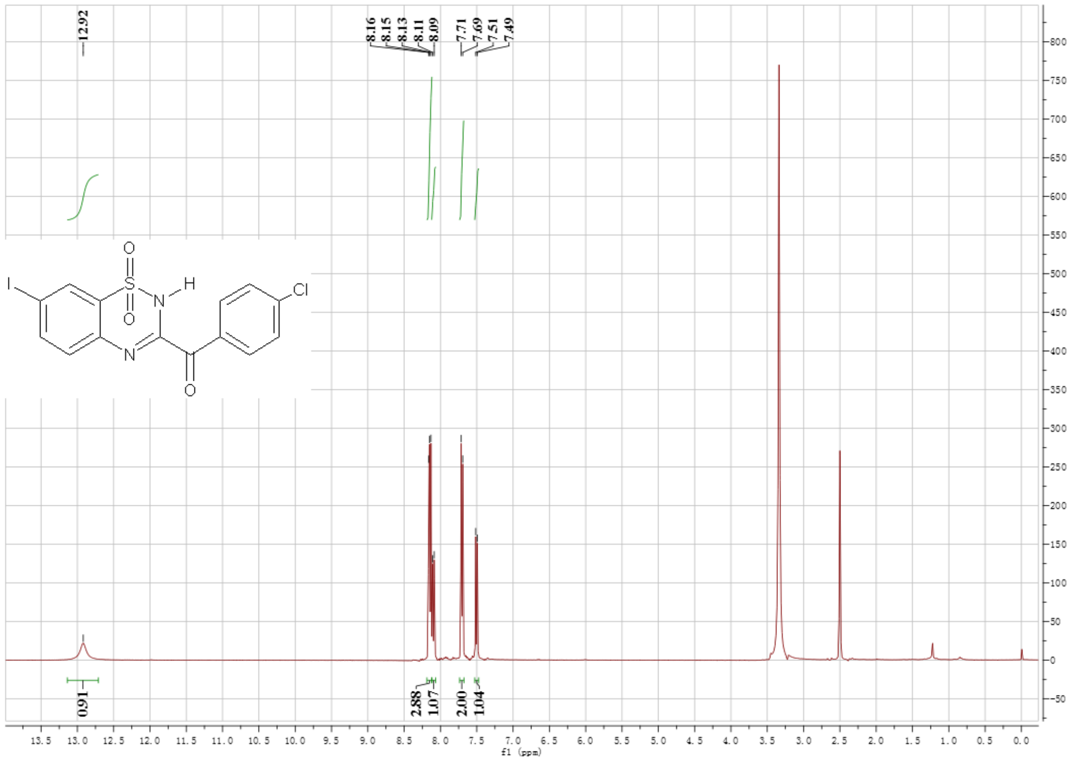

^{13}C NMR of compound 4b

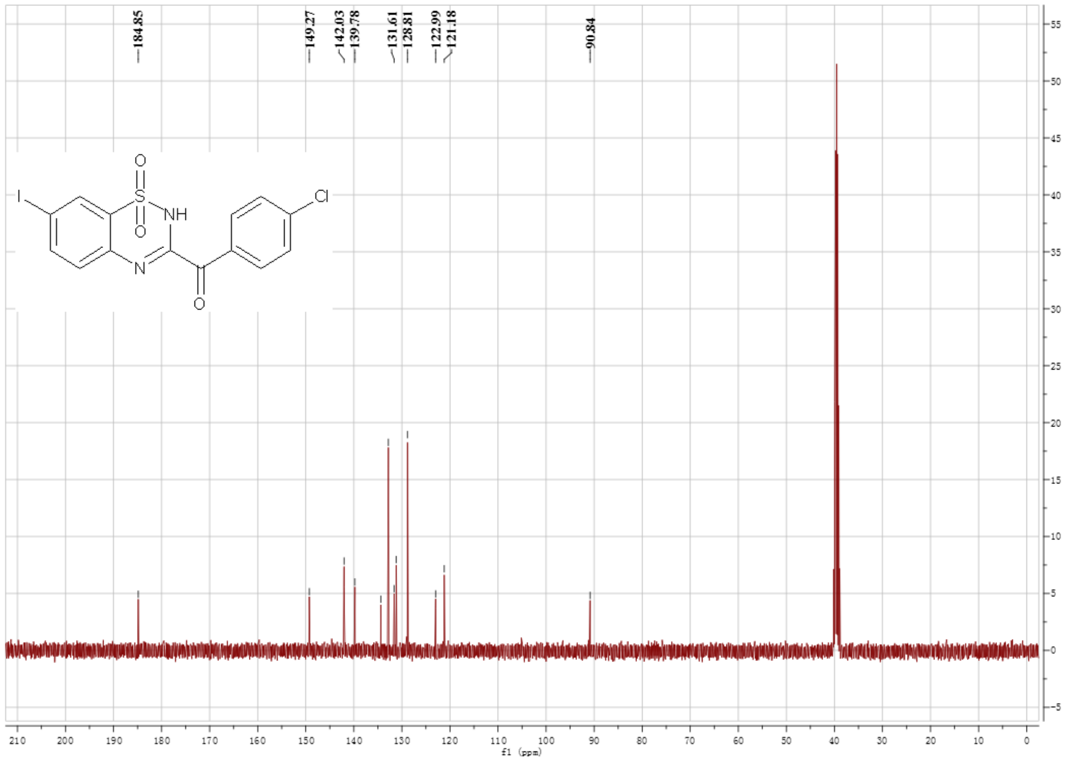

^{1}H NMR of compound 4c

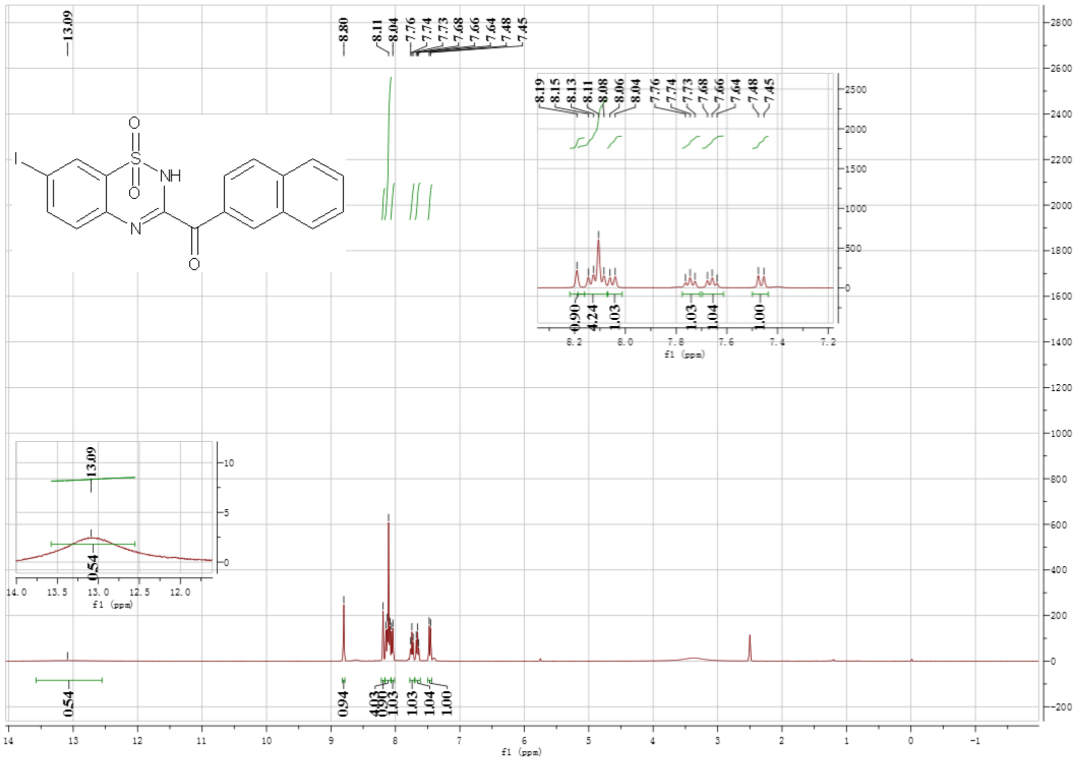

^{13}C NMR of compound 4c

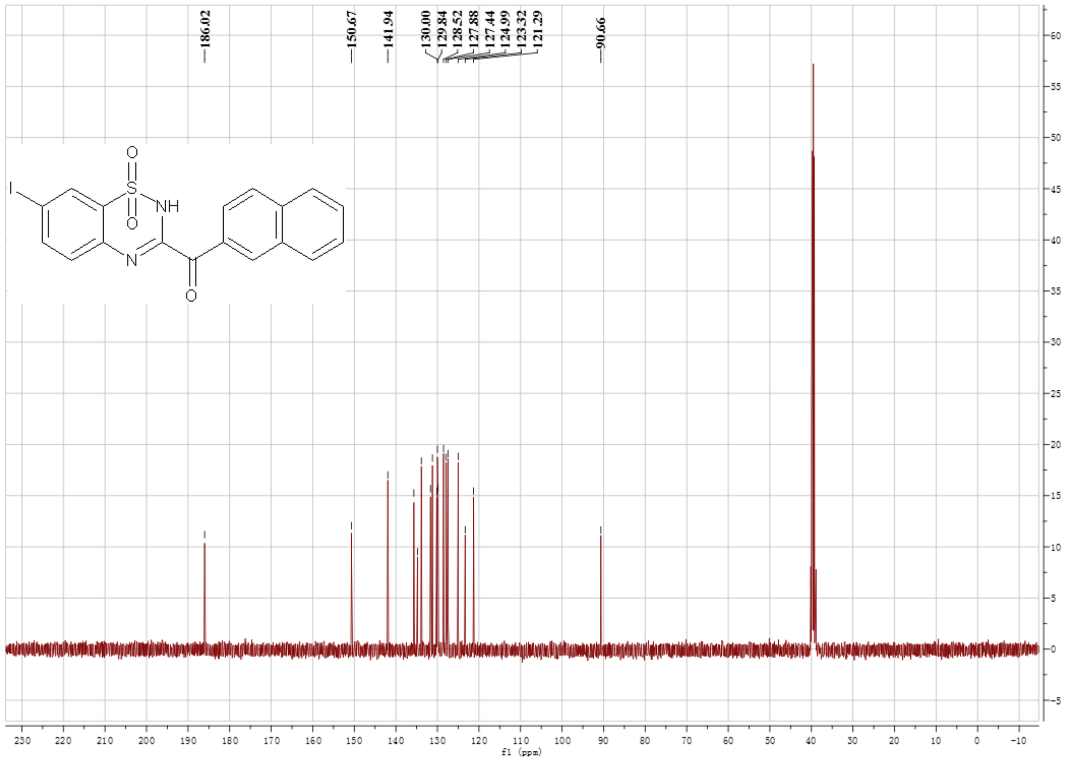

^{1}H NMR of compound 4d

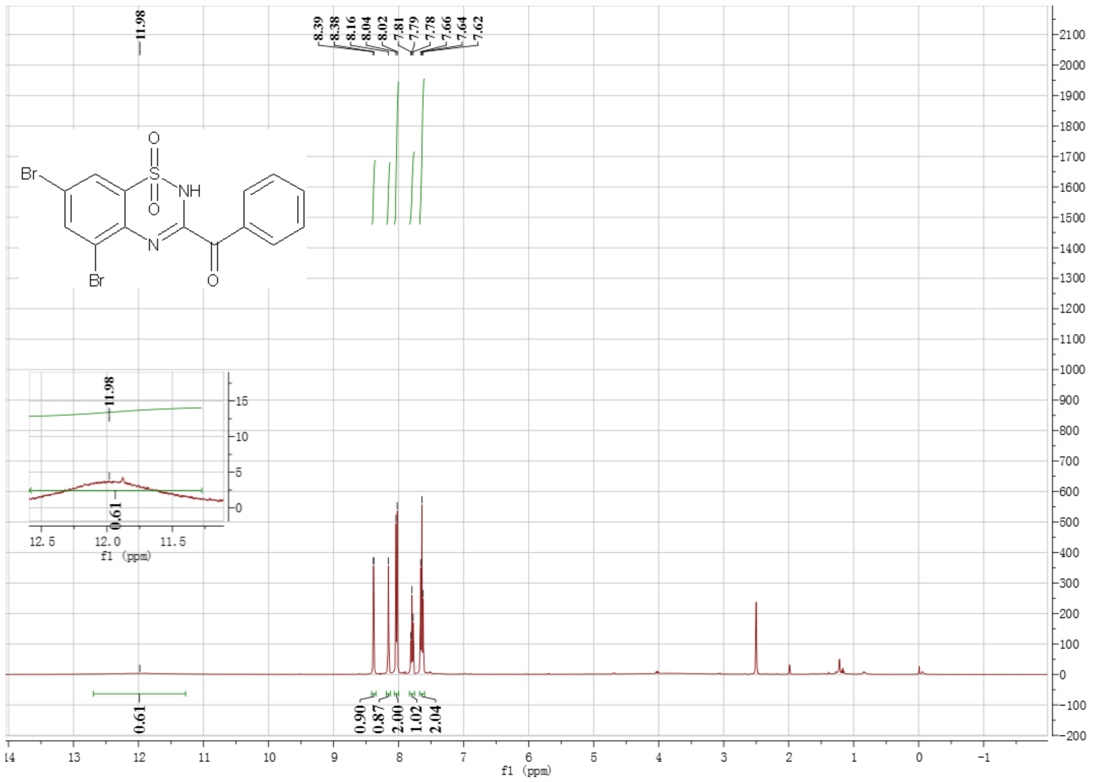

^{13}C NMR of compound 4d

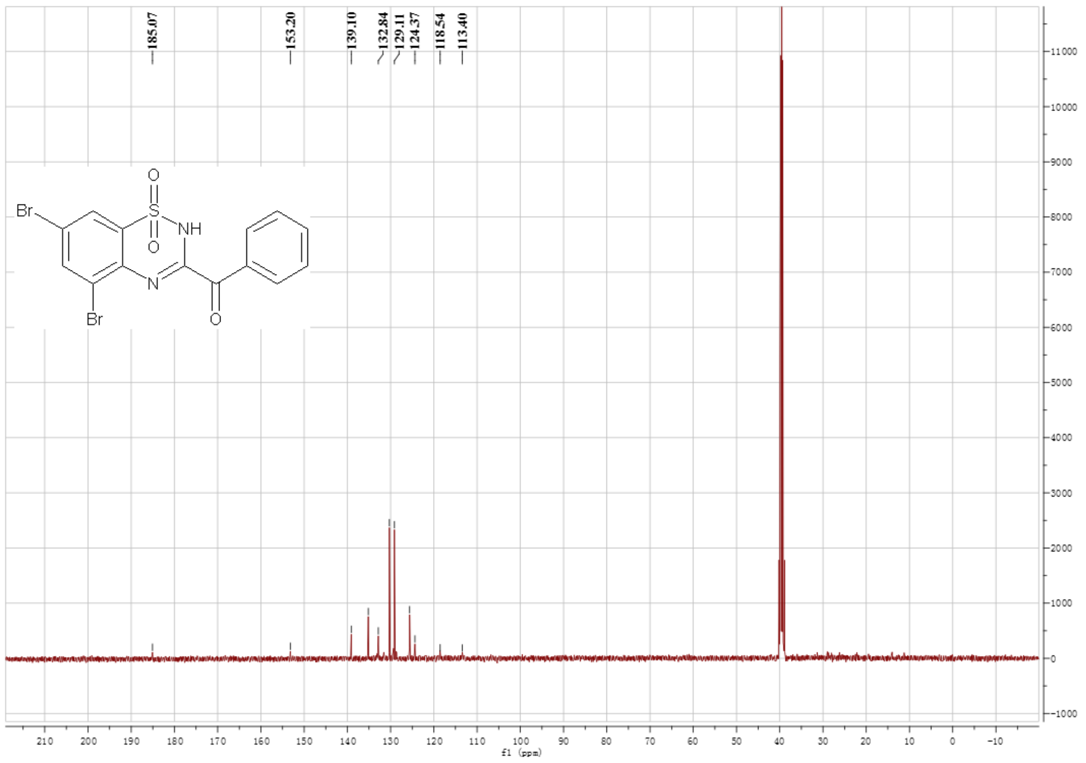

^{1}H NMR of compound 4e

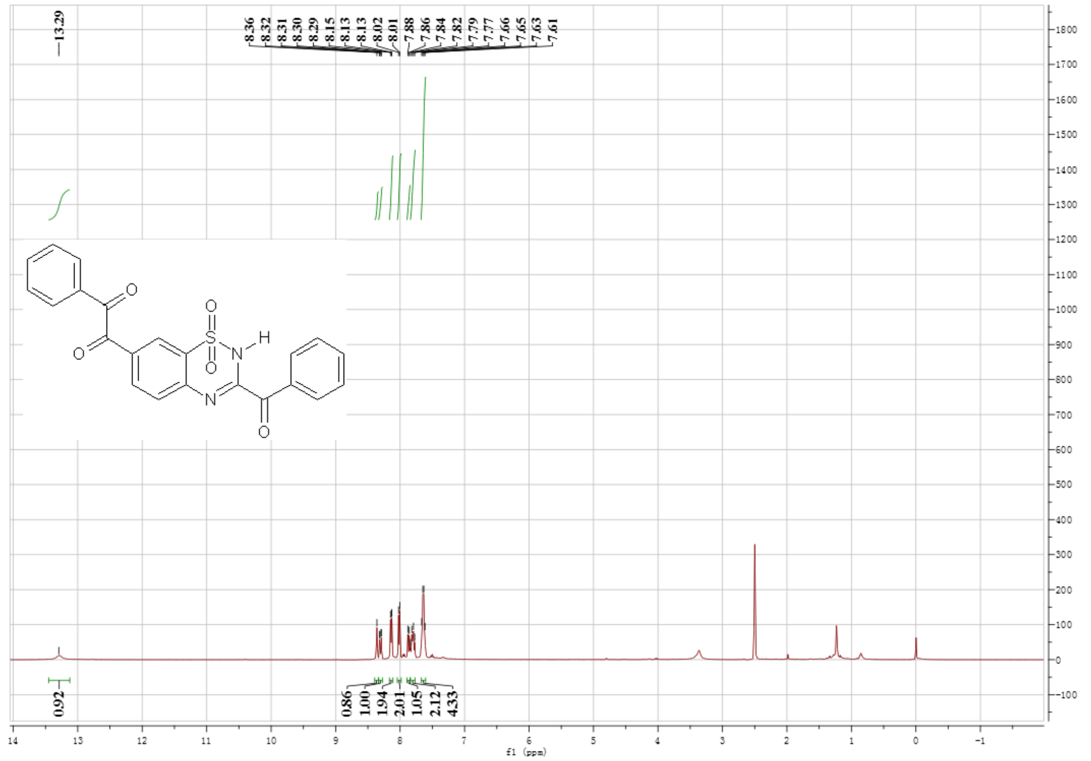

^{13}C NMR of compound 4e

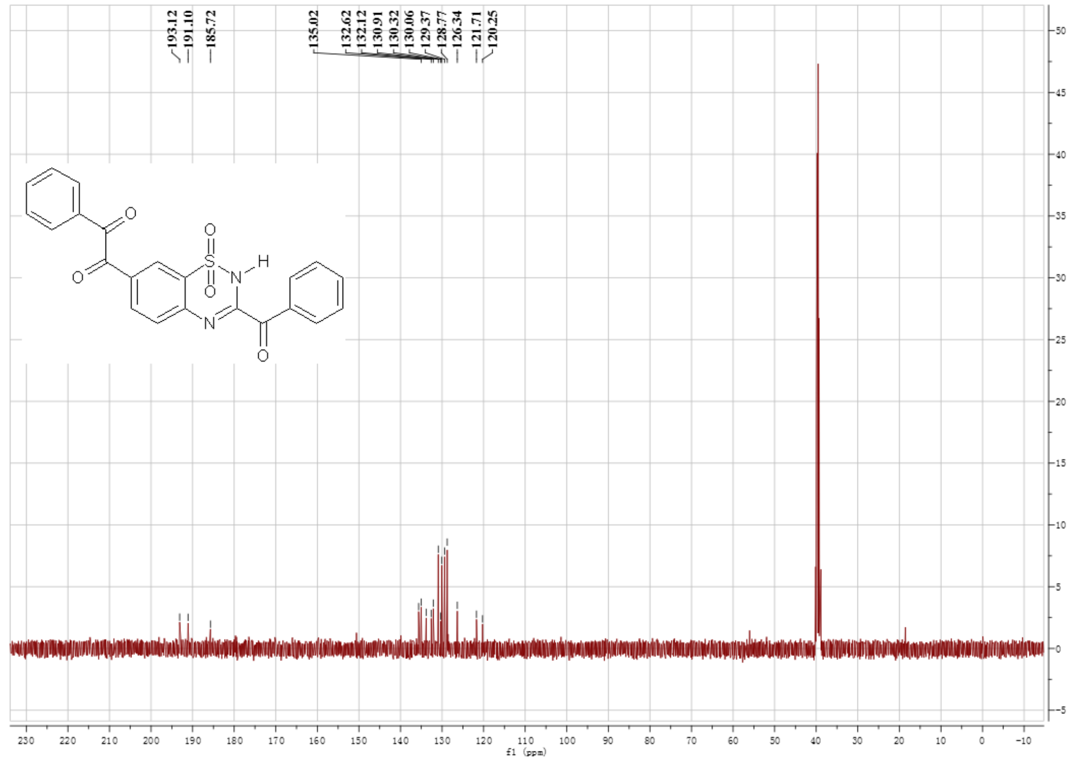

^{1}H NMR of compound 4f

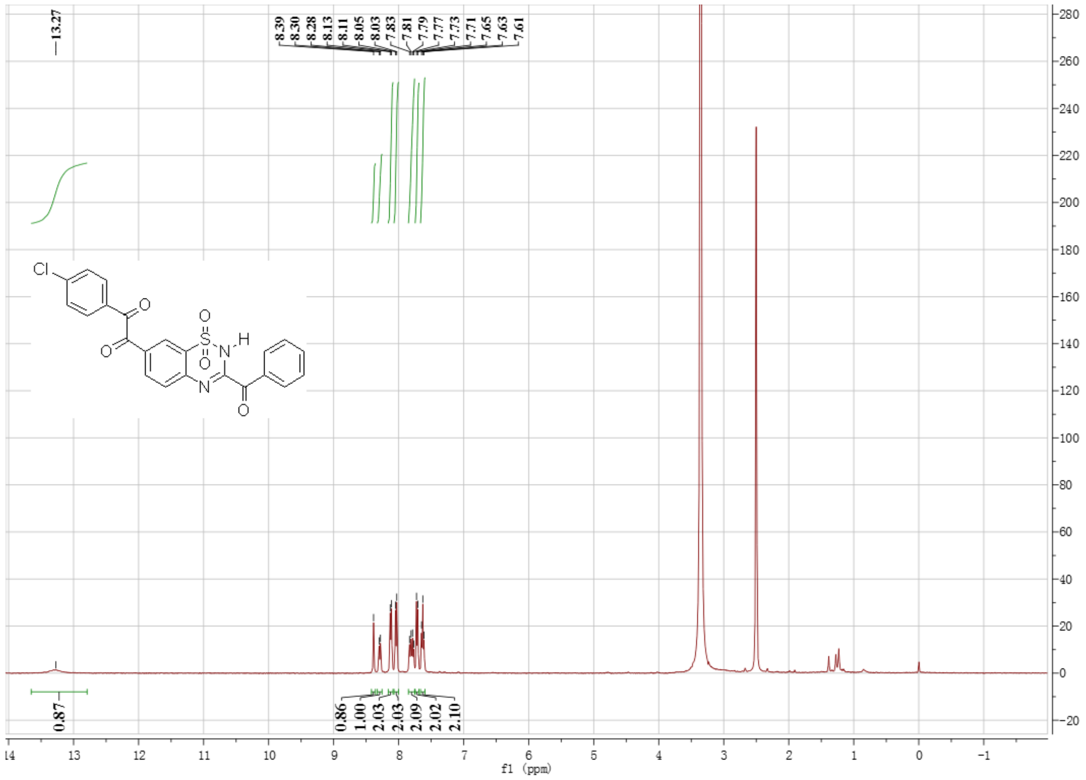

^{13}C NMR of compound 4f

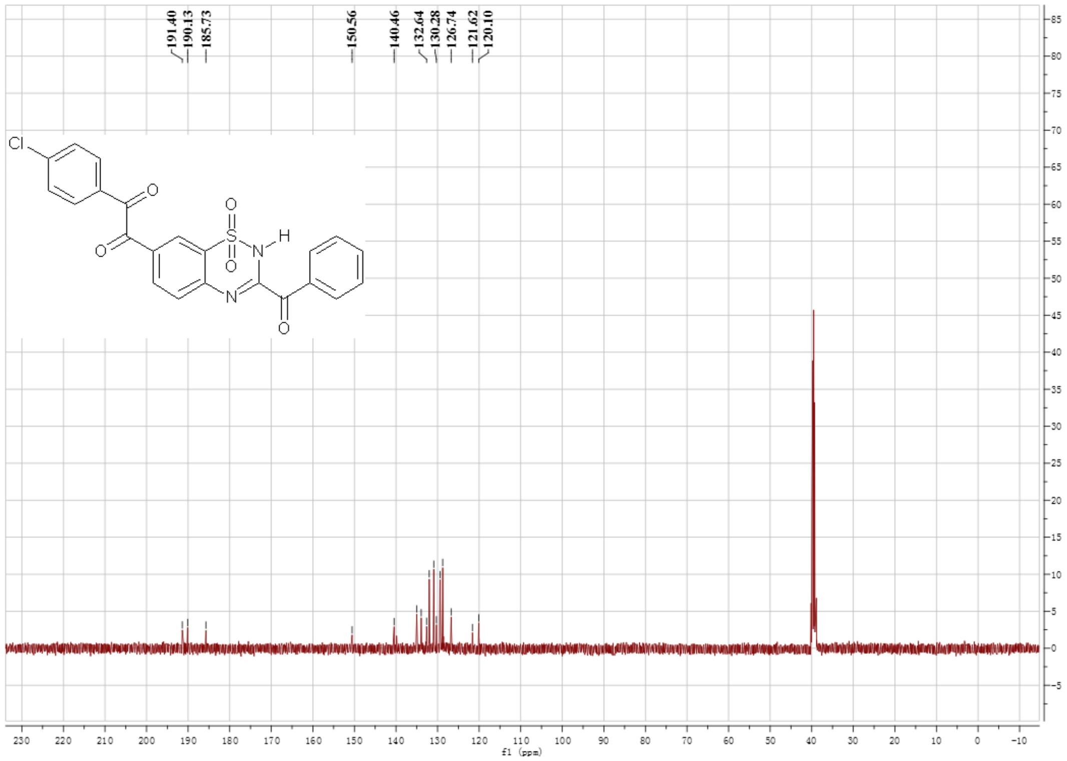

^{1}H NMR of compound 4g

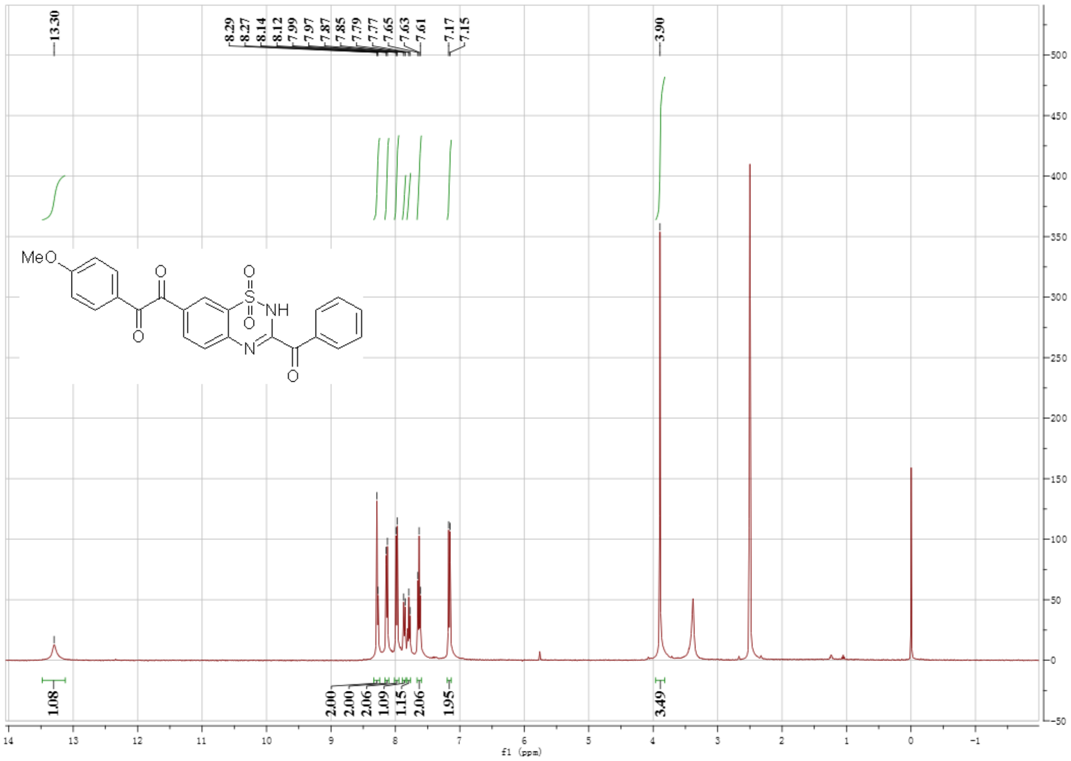

^{13}C NMR of compound 4g

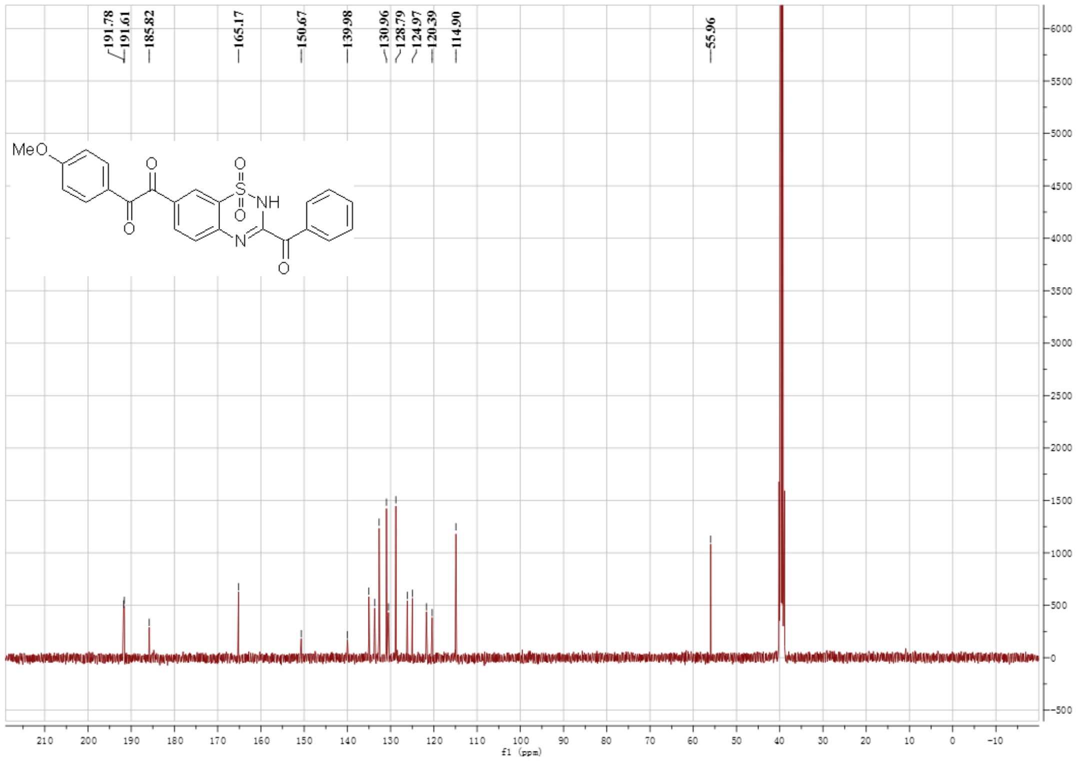

Supplement: File 1 — Experimental part and copies of NMR spectra. [file Beilstein_J_Org_Chem-12-1072-s001.pdf]
